# Drug-Resistance and Population Structure of *Plasmodium falciparum* Across the Democratic Republic of Congo Using High-Throughput Molecular Inversion Probes

**DOI:** 10.1093/infdis/jiy223

**Published:** 2018-04-28

**Authors:** Ozkan Aydemir, Mark Janko, Nick J Hathaway, Robert Verity, Melchior Kashamuka Mwandagalirwa, Antoinette K Tshefu, Sofonias K Tessema, Patrick W Marsh, Alice Tran, Thomas Reimonn, Azra C Ghani, Anita Ghansah, Jonathan J Juliano, Bryan R Greenhouse, Michael Emch, Steven R Meshnick, Jeffrey A Bailey

**Affiliations:** 1 Program in Bioinformatics and Integrative Biology, University of Massachusetts, Worcester; 2 Division of Transfusion Medicine, Department of Medicine, University of Massachusetts Medical School, Worcester; 3 Department of Geography, University of North Carolina, Chapel Hill; 4 Department of Epidemiology, Gillings School of Global Public Health, University of North Carolina, Chapel Hill; 5 Division of Infectious Diseases, University of North Carolina, Chapel Hill; 6 Curriculum in Genetics and Microbiology, University of North Carolina, Chapel Hill; 7 Institute for Global Health and Infectious Diseases, School of Medicine, University of North Carolina, Chapel Hill; 8 Medical Research Council Centre for Global Infectious Disease Analysis, Department of Infectious Disease Epidemiology, Imperial College London, United Kingdom; 9 Kinshasa School of Public Health, Hôpital General Provincial de Reference de Kinshasa; 10 Community Health, Kinshasa School of Public Health, School of Medicine, University of Kinshasa, Democratic Republic of Congo; 11 Division of Infectious Disease, University of California, San Francisco; 12 Department of Parasitology, Noguchi Memorial Institute of Medical Research, Ghana

**Keywords:** Democratic Republic of the Congo, malaria, drug resistance, molecular inversion probe, targeted sequencing

## Abstract

A better understanding of the drivers of the spread of malaria parasites and drug resistance across space and time is needed. These drivers can be elucidated using genetic tools. Here, a novel molecular inversion probe (MIP) panel targeting all major drug-resistance mutations and a set of microsatellites was used to genotype *Plasmodium falciparum* infections of 552 children from the 2013–2014 Demographic and Health Survey conducted in the Democratic Republic of the Congo (DRC). Microsatellite-based analysis of population structure suggests that parasites within the DRC form a homogeneous population. In contrast, sulfadoxine-resistance markers in dihydropteroate synthase show marked spatial structure with ongoing spread of double and triple mutants compared with 2007. These findings suggest that parasites in the DRC remain panmictic despite rapidly spreading antimalarial-resistance mutations. Moreover, highly multiplexed targeted sequencing using MIPs emerges as a cost-effective method for elucidating pathogen genetics in complex infections in large cohorts.

## Teaser

Probing all known drug-resistance loci using molecular inversion probes across the Democratic Republic of the Congo reveals prevalence, frequency, and geographical distribution of drug-resistant parasites, as well as spread of sulfadoxine-resistance mutations despite minimal official sulfadoxine use.

Malaria, particularly drug-resistant malaria, remains a global public health problem [1]. However, little is known about the drivers that modulate its spread over space and time [2]. Over short distances, parasite distribution depends on both human and mosquito movement among local populations, but over larger distances, human population movement is the main determinant [3]. Understanding such movement is important in the context of malaria control, especially with regard to the spread of drug-resistant parasites [4–7].

The Democratic Republic of the Congo (DRC) is the second largest country by area in Africa, and it has the second highest malaria burden [1]. It is centrally located, bordering 9 other countries. Because of this, the flow of parasites between the DRC and its neighbors and within the DRC needs to be better understood. Previously, using 5 neutral microsatellites (MSs), we were able to show that *Plasmodium falciparum* parasites in the DRC were different from those in other countries, but we could not find any differentiation within the country [8, 9]. However, we found notable geographic structure in more recently introduced genotypes, such as those resistant to sulfadoxine [10]. Parasites that harbor recently described deletions in the *pfhrp2* gene also showed spatial structure, clustering in a few sites in eastern DRC and in Kinshasa [11]. Based on these initial insights, information from more genetic markers and samples can likely further elucidate the population structure and flow of drug resistance.

Analyses of *P. falciparum* population genetics have been hampered by several factors. First, the AT richness of the genome makes sequencing difficult [12]. Second, individuals are often infected with multiple genotypes, making haplotype reconstruction challenging [13]. Third, balancing selection may independently lead to identical parasite populations in geographic regions that are not spatially connected, thereby giving the false impression of high connectivity [14]. Finally, a substantial portion of infections contain very low levels of circulating parasites, making it difficult to obtain enough parasite DNA relative to the human host for genotyping.

**Figure 1.**
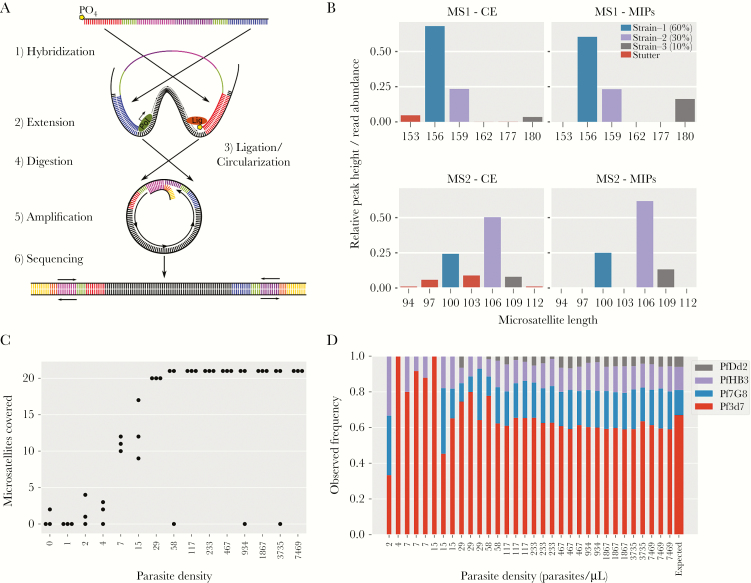
Molecular inversion probe (MIP) assay and performance on laboratory control mixtures. *A*, The MIP capture is illustrated showing the key steps of MIP arm hybridization, polymerase extension, and gap ligation to form a single-stranded circle. Exonuclease digestion removes linear template DNA, thereby relatively enriching for the circular captures, which are then amplified using universal primers along with a sample barcode. Important components are color coded: extension arm (blue), ligation arm (red) molecular identifiers (green), and backbone (pink+purple). *B*, An example of microsatellite (MS) stutter seen in standard capillary electrophoresis versus MIPs where stutter is detected and removed based on inconsistency within unique molecular identifiers. *C*, The coverage of the 21 assessed MSs, demonstrating that apart from a few failed reactions the vast majority of MSs are detected in every sample until dilutions of 29 parasites/uL. *D*, Frequency estimates of the 4-strain mixture compared with expected frequency (last bar on right, drawn wider for emphasis) based on relative amounts of DNA from each strain. Abbreviations: CE, capillary electrophoresis; MIP, molecular inversion probe; MS, microsatellite.

Here we introduce a panel of molecular inversion probes (MIPs) that can alleviate some of these problems through cost-effective targeted sequencing at multiple loci. Molecular inversion probe sequencing is a novel tool that has been used extensively for targeted capture and resequencing of human candidate genes, allowing tens of thousands of individual samples to be rapidly assessed at hundreds of loci (Figure 1A) [15–18]. Key strengths compared with other capture methods are scalability and minimal costs in terms of reagents and labor. Here, for the first time, we optimize MIPs for efficient capture from dried blood spots and use them to genotype and characterize malaria parasites across the DRC.

## METHODS

### Study Population

Dried blood spot samples were collected from children as part of cluster-based household surveys in the 2013–2014 DRC Demographic and Health Survey (DHS) from November 2013 to February 2014, as described previously [19]. DNA samples were extracted from 1622 dried blood spots and analyzed for *P. falciparum* by real-time quantitative polymerase chain reaction (qPCR). Each sample included global positioning system (GPS) coordinates that provide a location of the cluster of households from which they were collected [20]. The resulting 601 malaria-positive samples were selected and underwent MIP capture and sequencing at the University of Massachusetts. These positive samples came from 299 geographically distinct household cluster sites across all provinces within the country.

### Control Samples

The sensitivity and accuracy of the MIP panel was assessed using serial dilutions of a control mixture of DNA isolated from the laboratory strains 3D7, HB3, 7G8, and DD2 mixed at relative frequencies of 67%, 14%, 13%, and 6%, respectively (Supplementary Tables 1 and 2). Quantification was based on Quant-iT PicoGreen dsDNA Assay (Invitrogen, catalog no. P11496). These DNA mixtures were supplemented with 1 ng/µL of human DNA to better mimic DNA isolated from dried blood spots and whole blood and were included not only in initial testing but also as a positive control for every capture and sequencing run.

### *Plasmodium falciparum* Molecular Inversion Probe Design

Molecular inversion probes are approximately 100 nucleotide long, single-stranded oligos that have a shared backbone and specific sequence complementary to the target region in the arms (Figure 1A, Supplementary Figure 1*A*). *Plasmodium falciparum* MIPs were designed to capture known and candidate drug-resistance mutations (Table 1), as well as 11 previously described [21, 22] and 18 newly designed flanking MSs (Yaobao et al., unpublished data) (Table 2, Supplementary Table 3, Supplementary Methods). Nine of these MSs were removed from analysis for failing quality controls (Supplementary Methods).

**Table 1.** Summary of Drug-Resistance Molecular Inversion Probes

Gene	Targeted mutations	Number of MIPs
*PF3D7-1322700*	T236I	1
*PF3D7-1451200*	N71	1
*arps10*	V127M	1
*atp6*	L263E, E431K, A623E, S769N	7
*crt*	C72S, M74I, N75E, K76T, H97L, H97Y, A220S, N326S, I356T	7
*cytb*	M133I, Y268S, Y268C, V284K	2
*dhfr-ts*	A16V, N51I, C59R, S108T, I164L, T185	3
*dhps*	S436A, A437G, K540E, A581G, A613S	2
*fd*	D193Y	1
*k13*	M476I, Y493H, R539T, I543T, C580Y	9
*mdr1*	N86Y, Y184F, S1034C, N1042D, D1246Y	5
*mdr2*	T484I	1
*pib7*	C1484F	1
*pph*	V1157L	1

Abbreviation: MIP, molecular inversion probe.

**Table 2.** Summary of Microsatellite Molecular Inversion Probes

Region	Chromosome	Begin	End	Repeat unit	Repeat type	No. of MIPs	No. of MIPs passing QC
AS1	chr11	416541	416572	AAT	Trinucleotide	2	1
AS11	chr6	377496	377514	COMPLEX	Trinucleotide	2	1
AS12	chr6	372592	372612	AAT	Trinucleotide	2	1
AS13	chr6	372579	372621	ATA	Trinucleotide	1	1
AS14	chr5	1218960	1218986	AAC, AAT, ATTATGATA	Trinucleotide	2	2
AS15	chr13	2587730	2587758	ATA	Trinucleotide	2	1
AS19	chr4	533507	533554	ATA, ATT	Trinucleotide	2	2
AS2	chr11	416832	416890	AAT	Trinucleotide	2	1
AS20	chr4	536917	536949	TAA	Trinucleotide	1	0
AS21	chr4	528577	528599	TTA	Trinucleotide	2	0
AS25	chr10	1324819	1324890	ATT,ACT	Trinucleotide	1	0
AS3	chr11	417708	417739	TAA	Trinucleotide	2	0
AS31	chr6	806342	806371	ATT	Trinucleotide	1	1
AS32	chr12	1623232	1623297	ATT	Trinucleotide	1	1
AS34	chr12	2034957	2034977	ATT,ACT	Trinucleotide	2	1
AS4	chr11	418074	418095	TAA	Trinucleotide	2	2
AS7	chr6	899277	899296	ATT	Trinucleotide	1	1
AS8	chr6	894235	894269	ATT	Trinucleotide	2	2
Ara2	chr11	416315	416359	TAA	Trinucleotide	2	2
B7M19	chr10	1356173	1356265	T	Mononucleotide	2	1
PFG377	chr12	2045854	2045894	TAT	Trinucleotide	2	1
PfPK2	chr12	1611244	1611352	TAA	Trinucleotide	1	0
PolyAlpha	chr4	532213	532302	ATT	Trinucleotide	1	0
TA1	chr6	899867	900004	TAT	Trinucleotide	1	0
TA109	chr6	801053	801073	ACT, AATAATGATAAT	Trinucleotide	2	1
TA40	chr10	1322613	1322772	AAT	Trinucleotide	1	1
TA60	chr13	2588764	2588796	AAT	Trinucleotide	2	1
TA81	chr5	1214362	1214391	ATA	Trinucleotide	2	1
TA87	chr6	374755	374808	AAC, AAT	Trinucleotide	1	0

Abbreviations: MIC, molecular inversion probe; QC, quality control.

### Molecular Inversion Probe Capture, Amplification, and Sequencing

Our molecular inversion probe library sequencing is a multistep protocol modified from published protocols [23] to improve *P. falciparum* captures (Supplementary Methods, Supplementary Figures 1–4) with the following key steps. First a panel is created by pooling all desired MIPs (43 for MS MIP panel and 42 for drug-resistance MIP panel; Supplementary Table 3, probe sets MS1 and DR1, respectively), followed by 5’ end phosphorylation. Each panel can be used separately or combined to create a larger panel if desired. Each capture reaction is carried out as a single reaction per MIP panel per sample, combining sample DNA, MIP panel, polymerase, and ligase. With isothermal incubation, MIPs bind to their targets, followed by polymerase extension and single-stranded circle formation by ligase. After capture, all remaining linear DNA (unbound probes, original template DNA) is removed by exonuclease treatment. All captured products are then amplified by 1 forward and 1 reverse primer binding to the universal priming site on each circle. Polymerase chain reaction (PCR) primers also include Illumina sequencing adapters and 8-nucleotide-long sample barcodes. Once barcoded, samples are pooled into a single tube to create a sequence-ready library that is further cleaned prior to sequencing using solid phase reversible immobilization (SPRI) beads and agarose gel purification (Supplementary Figure 1*A*).

### Molecular Inversion Probe Data Processing

Sequencing data was processed using MIPWrangler software (Hathaway, unpublished data) in combination with other software. Briefly, sequences were demultiplexed by their dual sample barcode using bcl2fastq software (Illumina). Paired-end reads were then stitched together using FLASH [24] and filtered on expected length and on per base quality scores by discarding a sequence if the fraction of quality scores >30 was <70% (Q30 < 70%). Quality filtered stitched sequences were then further demultiplexed by target using the extension and ligation arm sequences to produce a file for each target for each sample (Supplementary Figure 1*B*).

Target sequences for each sample were then corrected using their unique molecular identifiers (UMIs). This was done by clustering sequences on their UMIs and then creating a consensus sequence for each specific UMI. This UMI redundancy removes a substantial proportion of PCR errors that occur in late cycles, including polymerase stutter and subsequent sequencing errors (Supplementary Figure 1*B*). The UMI corrected sequences were then further clustered by using the qluster algorithm from SeekDeep, allowing accurate detection of single base differences and indels at levels ≤1% [25]. Given the variable depth, we set minimum threshold defaults at ≥2 UMIs and ≥0.5% relative abundance for a cluster to be included in final analysis.

Differences between the observed sequence and the reference sequence for each probe were obtained by pairwise alignment using LastZ software [26]. Single nucleotide variants and indels from the LastZ output were annotated using Annovar software [27].

### Population Genetic Analyses of Microsatellites

Quality checks were carried out on the distribution of MS lengths, and all 20 loci were tested for independence through pairwise correlation tests using a Bonferroni-adjusted significance threshold of α = 0.05/190 to account for multiple testing. Principal component analysis (PCA) was conducted to look for any signal of population structure, using the dominant allele only in each individual and imputing missing values using the mean. Population structure was also assessed using the program *MavericK* [28], which builds on the Bayesian mixture model approach developed by Pritchard and colleagues [29] but provides more accurate estimates of the number of clusters. *MavericK* was run under the nonadmixture model with 500 burn-in iterations, 10000 sampling iterations, and 20 thermodynamic rungs. Finally, we looked for a signal of isolation by distance by regressing the absolute difference in MS lengths against geographic distance between sample GPS locations; under isolation by distance we would expect a positive relationship between these quantities.

### Spatial Analysis of Drug-Resistant Mutations

All drug-resistance loci were analysed separately using the R package PrevMap [30], which implements model fitting and spatial prediction under a range of geostatistical models. First, allele frequencies within each cluster were transformed to the real line using the transformation $y_{i}=log\left(\left(p_{i}+\varepsilon \right)/\left(1-p_{i}+\varepsilon \right)\right)$, where *p_i_* is the mutant allele frequency of cluster *i*, *y_i_* is the transformed value, and *ε* is a small value that ensures that *y_i_* values are always finite even when allele frequencies are 0 or 1 (we used *ε* = 0.0001, although results were not highly sensitive to this parameter). A geostatistical model of the form $y_{i}=S\left(x_{i}\right)+Z_{i}$ was then fit to the transformed data, where S(x) is a stationary isotropic Gaussian process (GP) with variance $\sigma^{2}$ and Matérn correlation function with scale ϕ and shape parameter κ=2, and $Z_{i}$ is an independent Gaussian noise term with variance $\tau^{2}$. Hence, the 3 free parameters of the model were $\left\{\sigma^{2},\phi ,\tau^{2}\right\}$. These parameters were jointly estimated in PrevMap using maximum likelihood, and fitted values were used to produce spatially continuous estimates of the underlying allele frequency distribution through 10000 simulations. These simulations capture the predictive error that occurs due to the stochastic nature of the underlying model. Standard errors of prediction were calculated and used to measure confidence at each point in space.

## RESULTS

### Molecular Inversion Probe Performance Controls

The accuracy and sensitivity of the designed MIP assay were tested using 15 serial dilutions of a DNA mixture of 4 laboratory strains (Supplementary Table 1) containing from 7469 down to 0.5 of *Plasmodium* haploid genome copies and 1 ng of human DNA (~1650 haploid genome copies) per microliter. Unique molecular identifiers associated with each arm allowed correction of errors introduced during PCR amplification (Supplementary Figure 1*B*), including MS stutter (Figure 1B). Based on these control mixtures, MIPs captured the vast majority of targets down to inputs of approximately 29 parasites per microliter (Figure 1C). The frequency estimates were consistent down to approximately 29 parasites per microliter, closely following the expected frequencies based on picogreen quantification of input DNA (Figure 1D).

### Sequencing the Parasitemic Demographic and Health Survey Blood Spots

**Figure 2.**
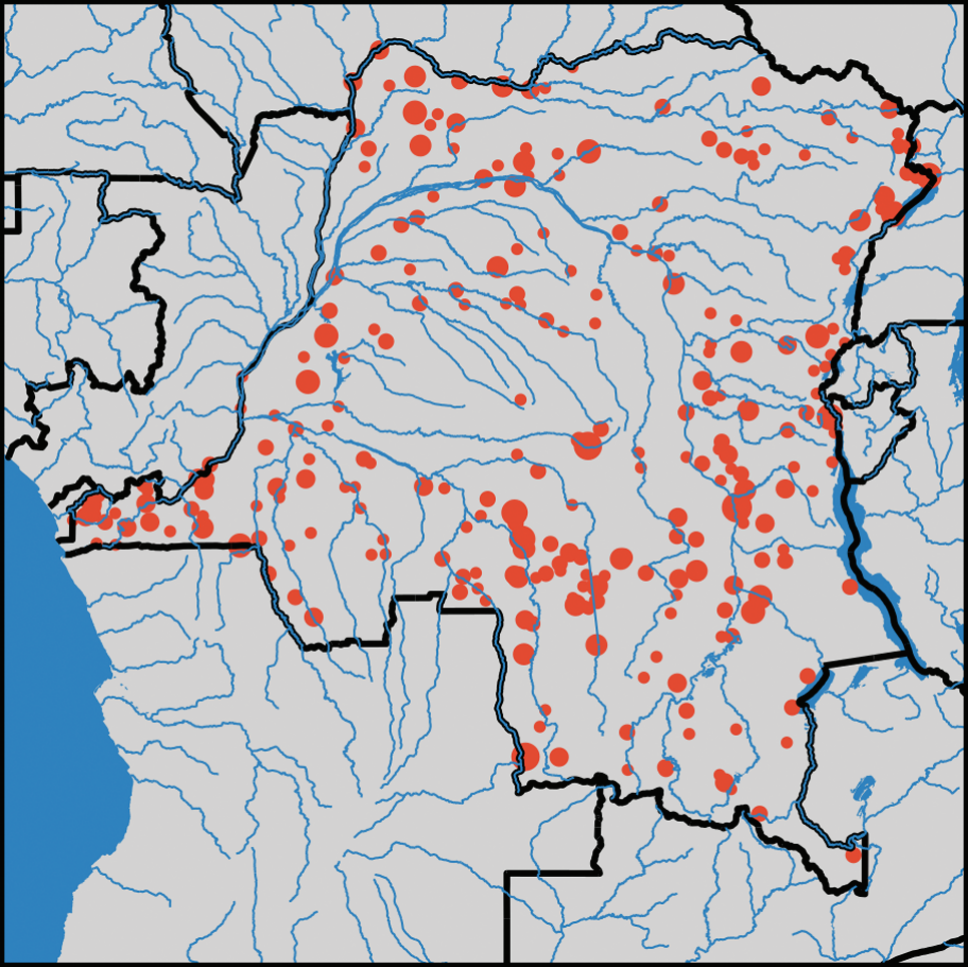
Distribution of 552 children with captured sequence. These samples were distributed across the Democratic Republic of the Congo without significant differences in the spatial distribution compared with the overall sample set or to the overall Demographic and Health Survey. The circle diameter is proportional to the number of samples from a given GPS location.

Among these 601 samples submitted for analyses, after a single MIP capture and sequencing run each for the drug-resistance and MS panels, 552 samples yielded a usable sequence. The sequenced individuals were well distributed across the country (Figure 2). Overall, 293 and 154 samples showed good coverage of 50% and 80%, respectively, across all of the targeted loci. In terms of density of infection, we found that MIP coverage began to drop off at qPCR cycle threshold (Ct) values of 31, which equates to a parasite level of approximately 100 parasites/uL (Supplementary Figure 5). This represents low parasitemia but may also be a consequence of the quality of the dried blood spots, as well as the large number of samples multiplexed. The median Ct value of the samples that yielded no MIP coverage was 34. The raw sequence data used in this study has been deposited to Sequence Read Archive of National Center for Biotechnology Information with the accession number SRP144456.

### Microsatellite Population Genetics

**Figure 3.**
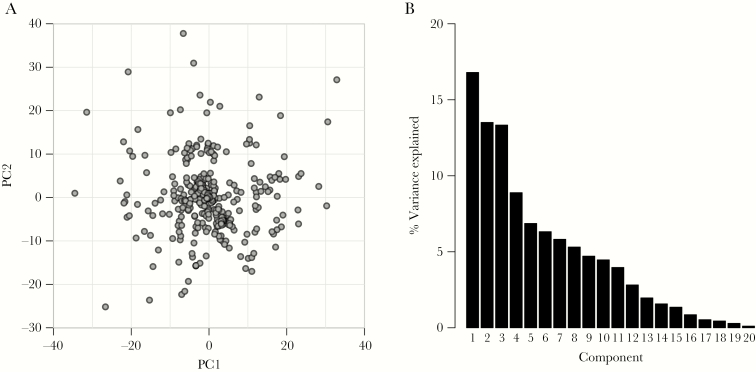
Principal component analysis across the Democratic Republic of the Congo. *A*, Scatterplot of the first and second principal components from the principal component analysis of the 20 microsatellites. *B*, Histogram of the percentage of variance accounted for by all 20 components. The first 2 components account for 30% of the total variation and show no clear population structure. Abbreviation: PC, principal component.

We first explored population structure using the 20 well-validated MSs from the MIP panel. Consistent with their known sequence, 19 MSs showed clear trinucleotide repeat distributions, with only MS B7M19 having a more complex repeat distribution (Supplementary Figure 6). Among the 19 trinucleotide MSs, just 2 of 7515 MS length calls fell outside the trinucleotide pattern, and these were removed from subsequent analyses. There was correlation in MS lengths in only 2 of the 190 pairwise comparisons—between Ara2 and AS1 and between AS12 and AS13. These MSs occur in close physical proximity, so this correlation indicates likely linkage disequilibrium. Principal component analysis indicated no clear signal of population structure, and the first 2 components accounted for a minimal amount of the total variation in the data (17% for PCA1, 14% for PCA2) (Figure 3, Supplementary Figure 7). Analysis in *MavericK* also failed to detect any population structure, with K = 1 clusters having the highest model evidence, indicating a single freely mixing population (Supplementary Figure 8). This was further supported by regression of genetic and geographic distance, which revealed no strong signal of isolation by distance (Supplementary Figure 9).

### Drug-Resistance Profiling

**Figure 4.**
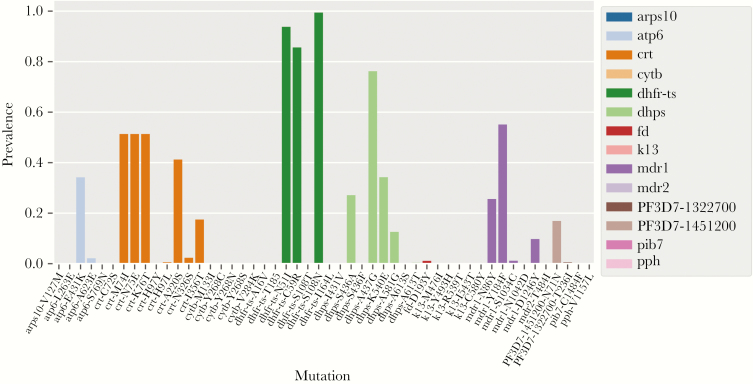
Countrywide prevalence of known drug-resistance mutations in infected individuals. Mutations are color-coded by gene showing the proportion of infections carrying known resistance-associated mutations. No known artemisinin-resistance mutations were observed.

Using the MIPs we designed to target known and putative drug-resistance mutations based on the literature. We first, quantified the prevalence of known drug-resistance mutations. The *pfdhfr-ts* gene mutations N51I, C59R, and S108N showed the highest prevalence (>80%) among all tested drug-resistance loci. Similar to the previous studies, the levels of evolutionarily older *pfdhps* mutations A437G and K540E showed higher prevalence compared with A581G [10, 31]. The mutations A613S/T and I431V seen in Western Africa [32] were not observed in the DRC. Mutations at the highest prevalences include those in *pfcrt* and *pfmdr1*. Importantly, known artemisinin-resistance mutations in *pfk13* (M476I, Y493H, R539T, I543T, C580Y) were not observed in any sample (Figure 4; Supplementary Table 6) consistent with previous studies [33, 34]. With deep sequencing, in addition to prevalence, we can also examine overall population frequencies of mutations. As expected, mutant allele frequencies were nearly identical to prevalences (Supplementary Figure 10) [35, 36].

### Spatial Assessment of Drug Resistance

**Figure 5.**
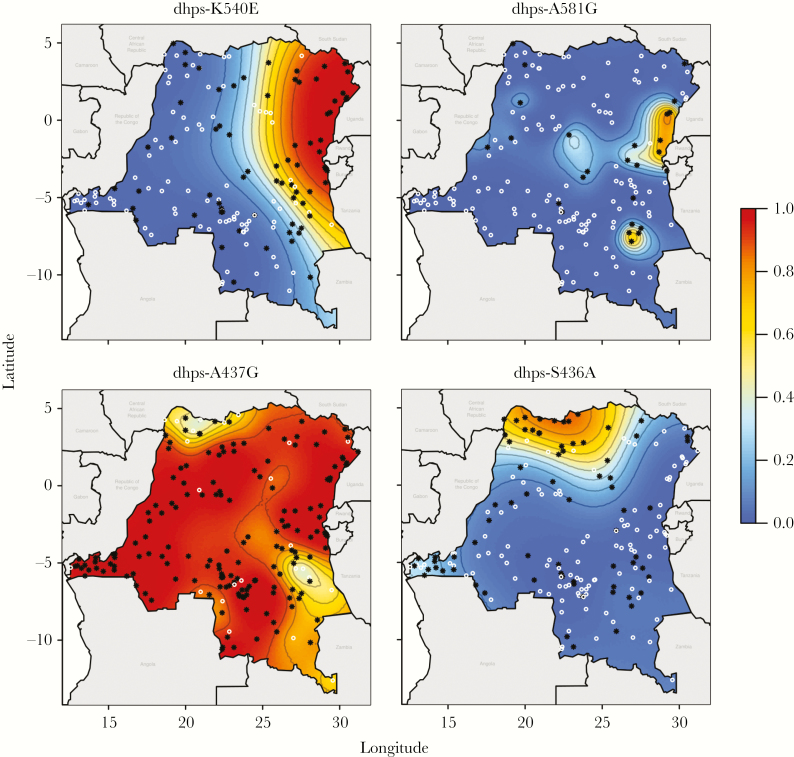
Spatial distribution of pfdhps mutations. Estimated prevalence of *pfdhps* K540E, A581G, A437G, and S436A mutations in the Democratic Republic of the Congo. White circles indicate clusters where only wild-type alleles were found; black stars indicate clusters where at least 1 mutation was found. Contours are at 10% prevalence levels.

Given the country-wide sampling scheme, we modeled the spatially referenced prevalence data using PrevMap to look for differences based on geographic location. For each mutation, we generated estimated prevalences and standard errors of prediction (Supplementary Figures 11 and 12). These predictions are generated using the maximum likelihood model parameters, so, although we present the most likely prevalence surfaces, it is important to emphasize there are alternative predictive surfaces that are plausible. Various spatial patterns were observed. Generally, older initial mutations or mutations that confer resistance to previous generations of drugs were relatively uniform in distribution. For instance, key mutations for chloroquine resistance and pyrimethamine resistance had relatively uniform prevalences across the DRC. Interestingly, what are thought to be relatively more recent adaptive mutations associated with sulfadoxine resistance showed strong spatial structure (Figure 5). The *pfdhps* K540E mutation was predominantly localized in the northeast, and, interestingly, the *pfdhps* A581G mutation was always found against a background of K540E. Conversely, the A437G mutation is seen in association with K540E and A581G mutations, as well as a single mutant *pfdhps* in the western side of the country. The S436A variant, not associated with drug resistance, occurs at high prevalence in the north of the country toward central Africa where it is most prevalent [37]. For *pfdhps*, the frequency of parasite mutations has significantly changed in comparison with our previous assessment of the 2007 DHS (*P* = 2.6 × 10^−5^, χ^2^ test, 3 df) [10]. Filtering our data to nonmixed *pfdhps* haplotypes equivalent to the 2007 analysis, the overall frequency of mutant parasites has increased markedly (43.5% in 2007 vs 72.1% in 2013; *P* = 1.5 × 10^−6^, Fisher exact test). The number of strains with sequential mutations, double *pfdhps* mutations (436S/437**G**/540**E**/581A—S**GE**A), and triple *pfdhps * mutations (S**GEG**) has increased significantly from 14.6% to 27.2% (*P* = .009). Overall, the single (S**G**KA and A**G**KA), double (S**GE**A), and triple (S**GEG**) mutants have increased 1.5-, 1.8-, and 2.2-fold, respectively, consistent with a greater selective advantage for triple mutant, fitting with its rapid spread where it was only observed approximating the northeast border in the 2007 DHS.

## DISCUSSION

Making statistically robust inferences from population genetics has been challenging for malaria. Many *P. falciparum* population genetic studies have used convenience samples, which may not be representative of the true parasite population and have the potential for substantial selection bias. Accordingly, we have used samples taken from the 2013–2014 DHS, which was a cluster-based household survey designed to be representative of the national population as a whole. To our knowledge, this is the first study to attempt to use a population-based sample of the malaria parasite to understand its subpopulation and drug-resistance structure and possible mechanisms of gene flow in the DRC.

Our results indicate that the parasite population is best characterized as a single population that exhibits weak isolation-by-distance. This is perhaps surprising given the country’s poor infrastructure and transportation network, which should lead to differentiation over relatively short spatial scales. Hence, it may be the case that transmission in the DRC is too high to measure differences in gene flow by traditional methods, with high levels of heterozygosity and the accompanying high genetic variability meaning that noise dominates any potential signal in the data.

In contrast, mutations in the *pfdhps* gene are very heterogeneously distributed and appear to be predominantly moving east to west with proportionally faster spread of the most resistant mutations. The *pfdhps* K540E mutation is concentrated in the eastern DRC, much more so than found previously in samples from the 2007 DHS [10]. Alarmingly, our data show that the *pfdhps* A581G mutation, observed in 2007, has further spread within the eastern DRC, now with a prevalence of 12.6% of infections with an allele frequency in the parasite population that has doubled from 3.3% to 7.4% frequency in a 6-year span. This combination of the A437G, K540E, and A581G triple mutations (GEG) is particularly ominous in that it is predictive of the failure of intermittent preventive therapy in pregnancy (IPTp) with sulfadoxine pyrimethamine (SP). The GEG triple mutant haplotype emerged in Tanzania in 2006, causing SP treatment failure when used for intermittent preventive treatment of malaria in infants [38]. It has since been reported in multiple countries in East Africa, including Rwanda, Uganda, Zambia, Malawi, Ethiopia, and the DRC [39–43], but is missing in West Africa [44–46]. Overall, the rates of increase in allele frequencies correlate with the number of mutations, consistent with the likely increased fitness conferred by sequential mutations. Although SP was no longer recommended as a first-line antimalarial in the DRC, the selective pressure may have been exerted by continued private sector use in addition to IPTp. A 2013 survey in Kinshasa showed that SP was the second most distributed treatment (31.1%) after non–quality-assured artemisinin combination therapy (38.5%) and indicated that SP may be being used as an inexpensive alternative to artemisinin combination threapy (ACT) outside of IPTp use [47]. Although more recent reports suggest that ACT availability has increased, the second most common partner drug in ACTs was SP (31.2%) in private pharmacies that provide the vast majority of antimalarials [48]. Providing maps, such as these, to national malaria control programs, should facilitate the appropriate choice of antimalarials at a local level.

The methods described here have a number of advantages over existing molecular surveillance platforms. First, MIPs minimize sequencing errors and can be used on small samples, such as dried blood spots. Second, the MIP platform we have developed is highly modular and scalable, so it can be expanded to include hundreds or thousands of additional targets and obtain much richer genetic data. Addition of new targets requires simply the design and synthesis of new oligos that can be added to existing panels. Molecular inversion probes also minimize effort and costs. For this study the work consumed 24 hours of technician time and $5000 in reagent and sequencing costs. Finally, all previous studies aggregate case reports or individual studies [31]; building on a nationally representative survey, such as the DHS, allows the calculation of local prevalences not only for malaria but also for drug-resistant malaria.

There are several other methods that have been used for measuring population structure in *P. falciparum*, including single nucleotide polymorphism (SNP) barcodes and whole genome analyses [49, 50]. Although useful, both of these methods have limitations. First, SNPs used in SNP barcodes are limited to those that are amenable to TaqMan genotyping assay and have limited ability to detect minority variants. Second, the assays are carried out individually for each genomic target, making the approach unscalable to large numbers of targets. Third, the information obtained from the SNP barcodes is limited to the known polymorphic SNPs and is insensitive to novel sequence variations. Whole genome sequencing, on the other hand, while addressing the limitations of SNP barcodes, is still too expensive to use for large cohorts. Furthermore, it is very difficult to assemble genomes from infections with a mixture of genotypes. Additionally, the host DNA contamination in most samples makes it hard to get good coverage of parasite DNA, especially in nonsymptomatic, low-parasite-density infections.

Importantly, this study has limitations. First, the low-density infections are difficult to sequence and are less represented compared with higher-density infections. Second, this study relied on data sampled from children rather than across all ages. Finally, the methods used for the DRC, a very high transmission country, may not be directly applicable to countries with lower malaria transmission. Nevertheless, the MIP protocol used here allows for genotyping of malaria parasites at scale, both in terms of number of samples and number of loci. As such, it should prove useful for other objectives in molecular surveillance—for example of known drug-resistant or other mutations of public health importance.

## Supplementary Data

Supplementary materials are available at *The Journal of Infectious Diseases* online. Consisting of data provided by the authors to benefit the reader, the posted materials are not copyedited and are the sole responsibility of the authors, so questions or comments should be addressed to the corresponding author.

Supplementary Table1

Supplementary Table2

Supplementary Table3

Supplementary Table4

Supplementary Table5

Supplementary Table6

Supplementary Table7

Supplementary Figure1

Supplementary Figure2

Supplementary Figure3

Supplementary Figure4

Supplementary Figure5

Supplementary Figure6

Supplementary Figure7

Supplementary Figure8

Supplementary Figure9

Supplementary Figure10

Supplementary Figure11

Supplementary Figure12

Supplementary Methods

## Notes

***Financial support.*** This work was supported by the National Institutes of Health (R01AI107949-04, R21AI121465, K24AI134990, R01AI099473, T32GM107000), the National Science Foundation (BCS-1339949), and UK Medical Research Council (MR/N01507X/1). M. M. J. received support from the Royster Society of Fellows at UNC-CH and from the Population Research Infrastructure Program awarded to the Carolina Population Center (P2C HD050924) by the Eunice Kennedy Shriver National Institute of Child Health and Development. R. V. is funded by a Skills Development Fellowship: this award is jointly funded by the UK Medical Research Council (MRC) and the UK Department for International Development (DFID) under the MRC/DFID Concordat agreement and is also part of the EDCTP2 program supported by the European Union.

***Potential conflicts of interest.*** All authors: No reported conflicts of interest. All authors have submitted the ICMJE Form for Disclosure of Potential Conflicts of Interest. Conflicts that the editors consider relevant to the content of the manuscript have been disclosed.

Presented in part: “The Future of Malaria” meeting, Baltimore, Maryland, November 2017. Abstract A-08.
