# Supplementary material for: Drug-Resistance and Population Structure of Plasmodium falciparum Across the Democratic Republic of Congo Using High-Throughput Molecular Inversion Probes
Source: J Infect Dis. 2018 Apr 28;218(6):946–55. doi: 10.1093/infdis/jiy223 (PMC6093412; doi:10.1093/infdis/jiy223)
Supplement: Supplementary Figure1 [file jiy223_suppl_supplementary_figure1.docx]

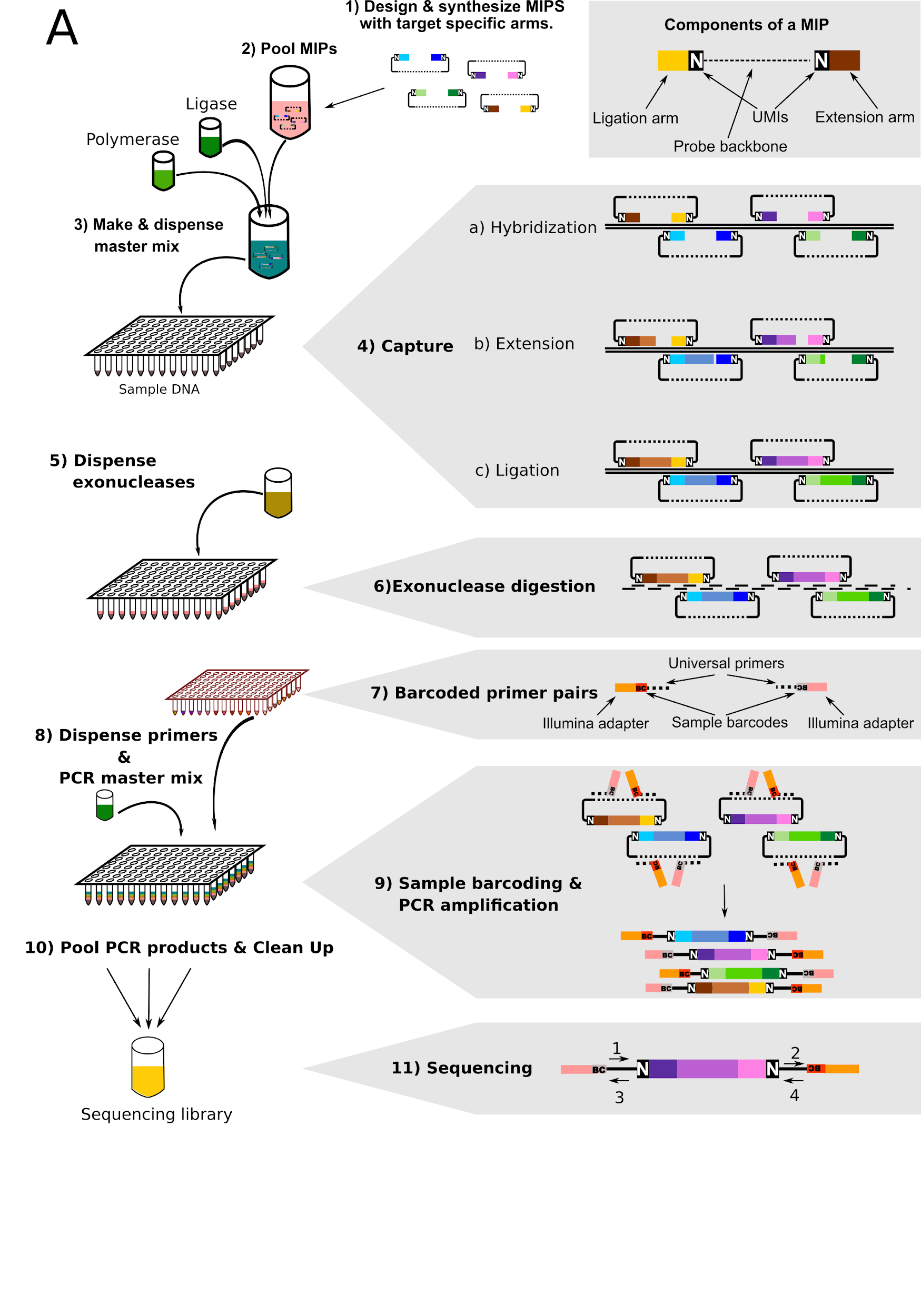


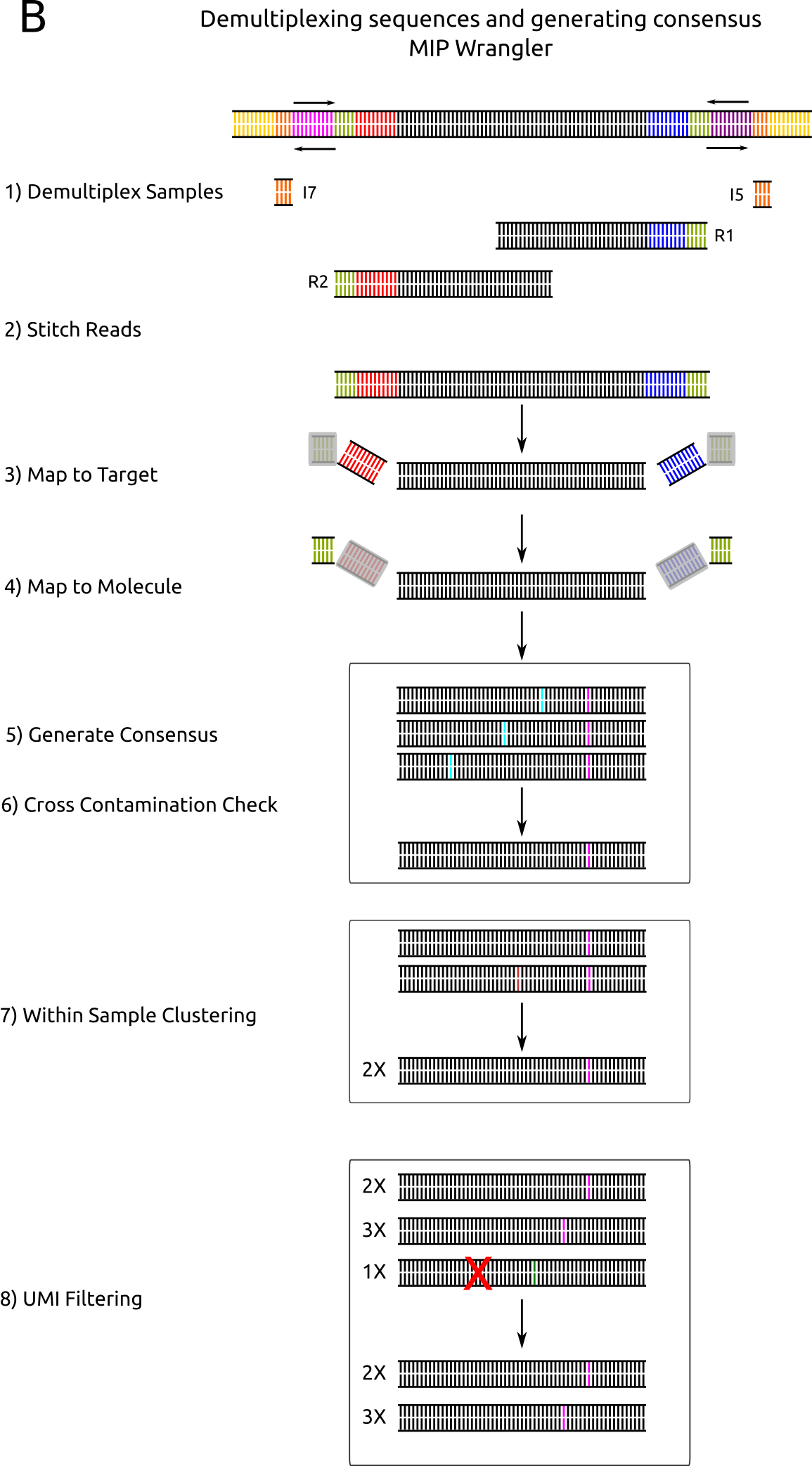


**Supplementary Figure 1. A) MIP workflow and experimental multiplexing.** A MIP is composed of two arms complementary to its target, connected in the middle by a 36 bases long backbone sequence that is the same for all MIPs. There are 14 random bases between the target specific arms and the backbone, forming the unique molecular identifiers (UMIs) (**Box: Components of a MIP**). MIPs are designed and synthesized for different targets (1) and pooled for use as a single panel of MIPs, massively parallelizing the capture process (2). A 96 well workflow is used to minimize human error and facilitate processing large number of samples (3-10). A master mix of Pooled MIPs, polymerase and ligase are dispensed into a plate containing sample DNA (3) and incubated to perform the capture reaction. In the capture reaction, MIPs hybridize to their specific targets via the target specific arms (4a). After binding, DNA polymerase fills in the gap starting from the “extension arm” (4b) and ligase closes the gap once the polymerase reaches he ligation arm (4c). After the capture reaction, addition of exonucleases to each well (5) removes all probes that have not been circularized as well as the template DNA, leaving only the circularized captured target DNA in the wells (6). A PCR reaction is set up by adding to each well one forward and one reverse primer each of which contain one of 384 possible 8-base barcodes so that each sample is uniquely identifiable, in addition to adapter sequences common to all forward or reverse primers which are required for Illumina sequencing (7-8). It is theoretically possible to multiplex over 100,000 samples with these barcoded primers . Each forward and reverse primer pair have a universal sequence that binds to the probe backbone which is shared by all capture products (7, 9). This helps reducing PCR bias due to primer efficiency. After the PCR reaction, all captured target sequences include a sample barcode (9) and are pooled together and DNA clean up is performed to remove adapter and primer dimers (10). Libraries are sequenced using Illumina’s MiSeq platform with dual indexing. Four sequencing primers used sequentially generate sequences for forward and reverse captured sequence and the forward and reverse sample barcodes (11)

**B) Bioinformatic demultiplexing.** Following illumina sequencing Samples are demultiplexed based on the forward and reverse sample barcodes (orange), (1). Paired end reads are stitched using FLASH software to generate a single sequence for each read pair (2). Using extension and ligation arms (blue and red, respectively) each sequence target is identified (3). Using UMIs (green) on each side of capture, sequences that are PCR duplicates which originate from the same template DNA molecule are identified (4). Sequence reads sharing the same UMIs were collapsed to generate a single consensus. This consensus allows removal of , errors (aqua) caused in PCR cycles and sequencing, leaving only true variants (pink) (5). A cross sample check for shared sequence reads with same UMIs detects and removes any cross contamination (6). UMI collapsed sequences are further clustered within sample and collapsed based on sequencing quality, removing likely sequencing errors (red), (7). A minimum UMI criteria (>=2) is used to remove rare errors occurring in the initial rounds of PCR (green)(8), assuming that it is unlikely to have the same PCR error in independent UMIs within one sample.
