# Supplementary material for: Drug-Resistance and Population Structure of Plasmodium falciparum Across the Democratic Republic of Congo Using High-Throughput Molecular Inversion Probes
Source: J Infect Dis. 2018 Apr 28;218(6):946–55. doi: 10.1093/infdis/jiy223 (PMC6093412; doi:10.1093/infdis/jiy223)
Supplement: Supplementary Figure2 [file jiy223_suppl_supplementary_figure2.docx]

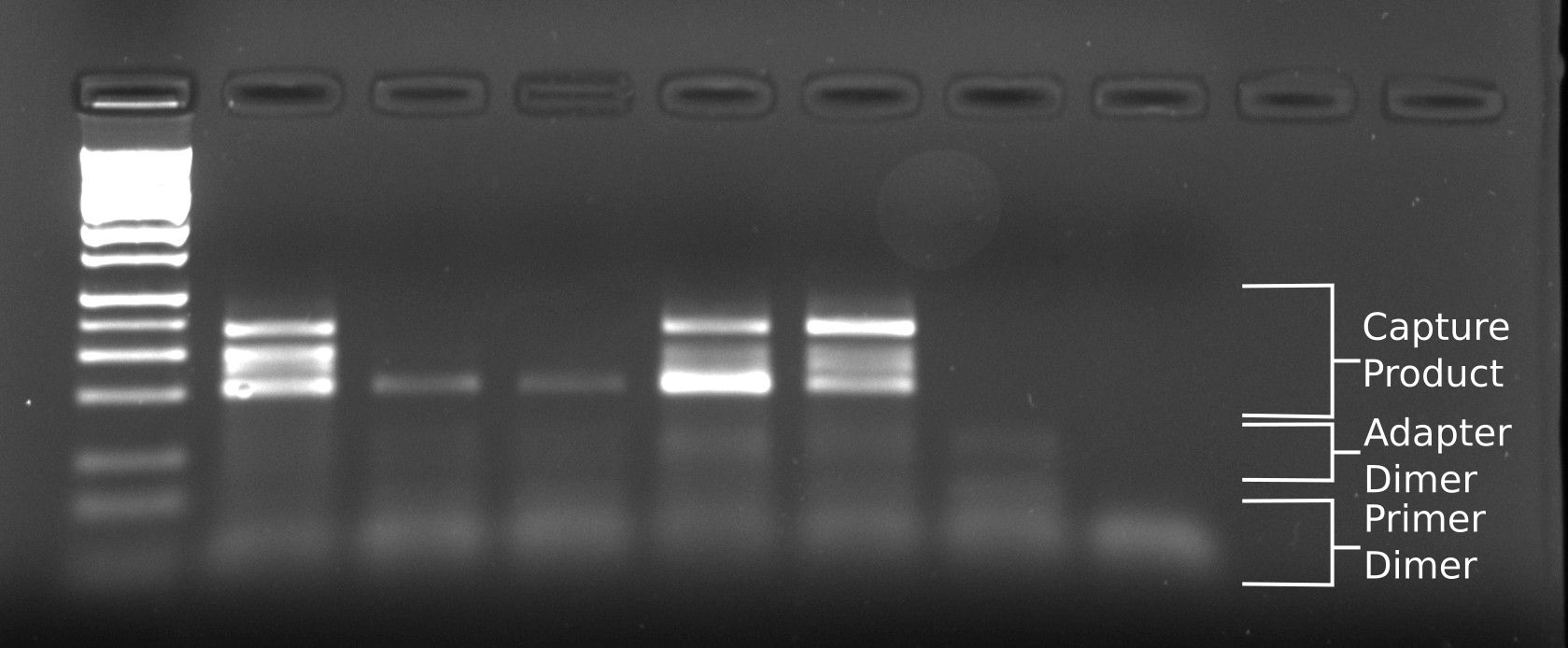


| Lane | 1 | 2 | 3 | 4 | 5 | 6 | 7 | 8 |
| --- | --- | --- | --- | --- | --- | --- | --- | --- |
| Enzyme |  | Phusion | Phusion | Phusion | Phusion | KlenTaq | Phusion | Phusion |
| Capture Time (h) |  | 1 | 1 | 1 | 24 | 24 | 24 | 24 |
| Buffer Included in Denaturation |  | + | - | - | + | + | + | + |
| Template DNA | Ladder | + | + | + | + | + | - | - |

### ***Supplementary Figure 2. Capture optimization for time and enzyme***

For optimization experiments, all parameters except the parameter being optimized was kept constant and the reaction efficiency was determined by visual inspection of the capture product on an agarose gel. Optimized parameters: capture time, denaturation buffer, polymerase enzyme. 1 h capture time worked as well as the published 24 h, lanes 2, 5 and 6, respectively. Excluding ampligase buffer at denaturation step reduced capture efficiency (lanes 3 and 4). Phusion polymerase enzyme works comparably to KlenTaq (compare lanes 1, 5 and 6).
