# Supplementary material for: Drug-Resistance and Population Structure of Plasmodium falciparum Across the Democratic Republic of Congo Using High-Throughput Molecular Inversion Probes
Source: J Infect Dis. 2018 Apr 28;218(6):946–55. doi: 10.1093/infdis/jiy223 (PMC6093412; doi:10.1093/infdis/jiy223)
Supplement: Supplementary Figure3 [file jiy223_suppl_supplementary_figure3.docx]

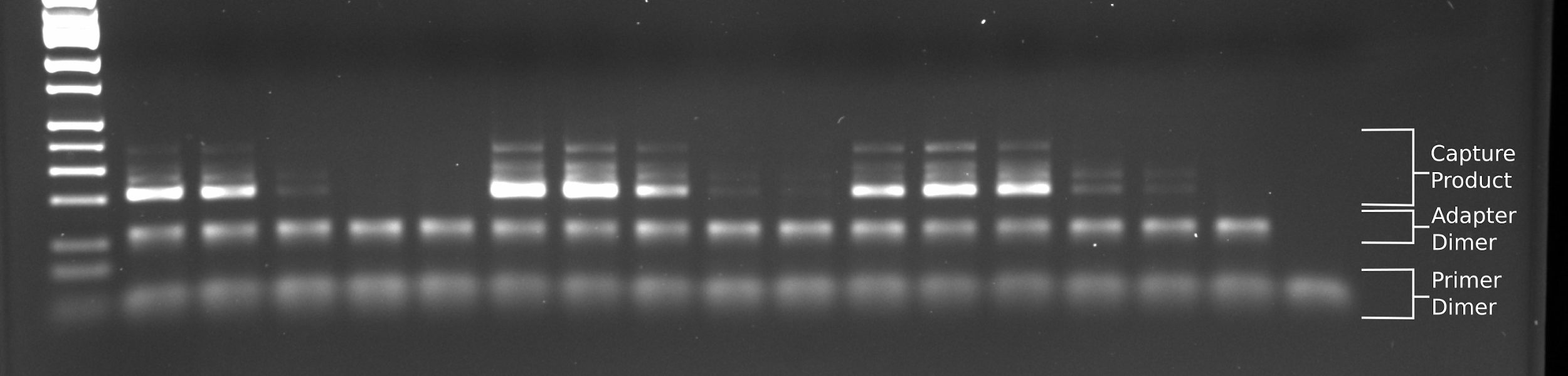


| Lane | 1 | 2 | 3 | 4 | 5 | 6 | 7 | 8 | 9 | 10 | 11 | 12 | 13 | 14 | 15 | 16 | 17 | 18 |
| --- | --- | --- | --- | --- | --- | --- | --- | --- | --- | --- | --- | --- | --- | --- | --- | --- | --- | --- |
| Enzyme Dilution |  | 100 | 200 | 1K | 5K | 10K | 100 | 200 | 1K | 5K | 10K | 100 | 200 | 1K | 5K | 10K | 100 | - |
| Capture Temp. |  | 55 | 55 | 55 | 55 | 55 | 60 | 60 | 60 | 60 | 60 | 65 | 65 | 65 | 65 | 65 | 60 | - |
| Template DNA | L | + | + | + | + | + | + | + | + | + | + | + | + | + | + | + | - | - |

### ***Supplementary Figure 3. Optimization of capture temperature***

Optimized parameters: capture temperature and polymerase dilution. Polymerase dilution of 1:200 and capture temperature of 60°C (lane 8) was chosen for use in following experiments due to having the highest yield.
