# Supplementary material for: Drug-Resistance and Population Structure of Plasmodium falciparum Across the Democratic Republic of Congo Using High-Throughput Molecular Inversion Probes
Source: J Infect Dis. 2018 Apr 28;218(6):946–55. doi: 10.1093/infdis/jiy223 (PMC6093412; doi:10.1093/infdis/jiy223)
Supplement: Supplementary Figure4 [file jiy223_suppl_supplementary_figure4.docx]

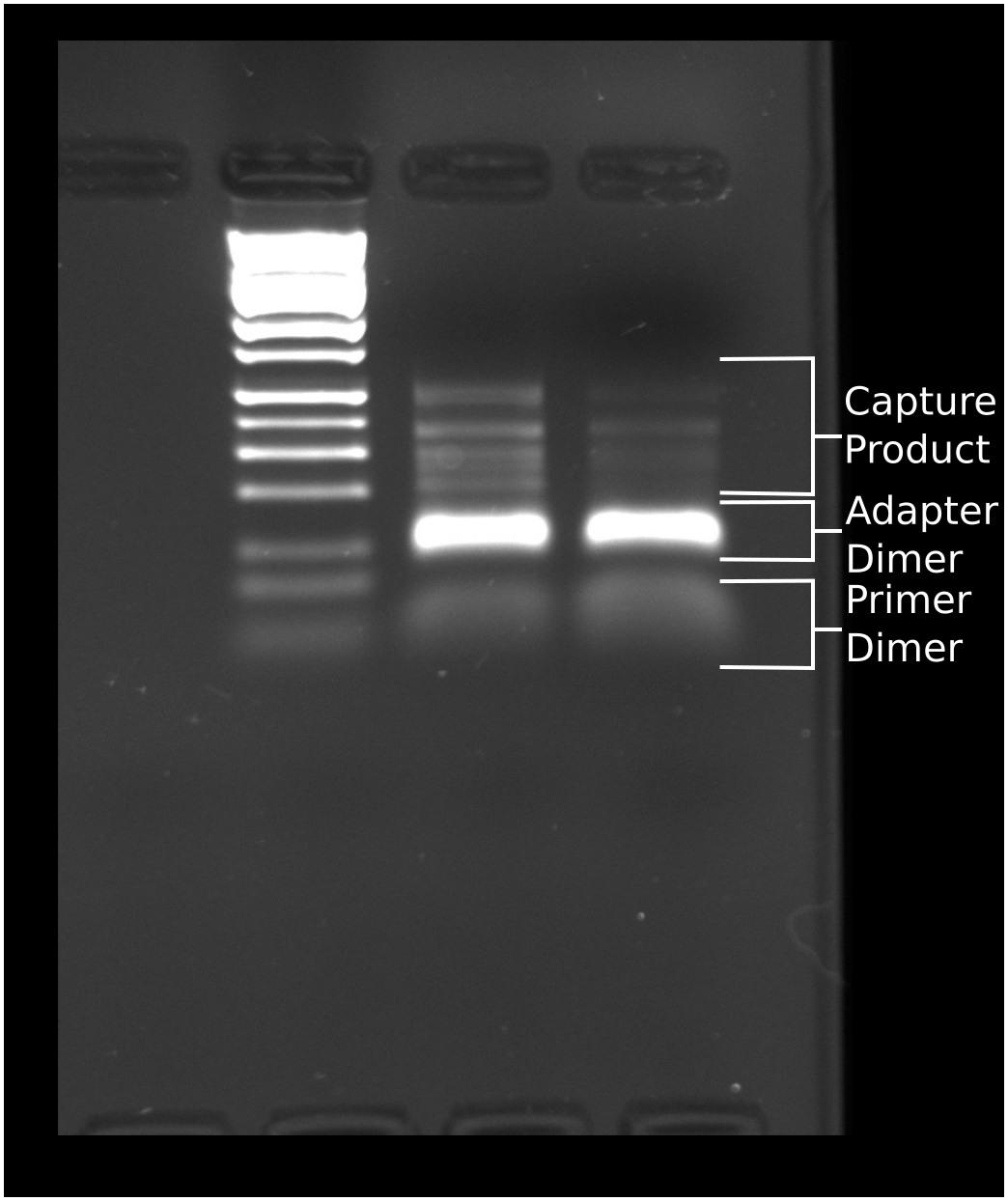


| Lane | 1 | 2 | 3 |
| --- | --- | --- | --- |
| Capture Volume (µl) | Ladder | 10 | 25 |

##

### ***Supplementary Figure 4. Optimization of capture reaction volume***

Optimized parameter: capture volume. Capture volume optimization. Smaller (10 µl) total capture reaction volume works better compared to larger (25 µl) volume.
