# Supplementary material for: Drug-Resistance and Population Structure of Plasmodium falciparum Across the Democratic Republic of Congo Using High-Throughput Molecular Inversion Probes
Source: J Infect Dis. 2018 Apr 28;218(6):946–55. doi: 10.1093/infdis/jiy223 (PMC6093412; doi:10.1093/infdis/jiy223)
Supplement: Supplementary Figure5 [file jiy223_suppl_supplementary_figure5.docx]

### ***
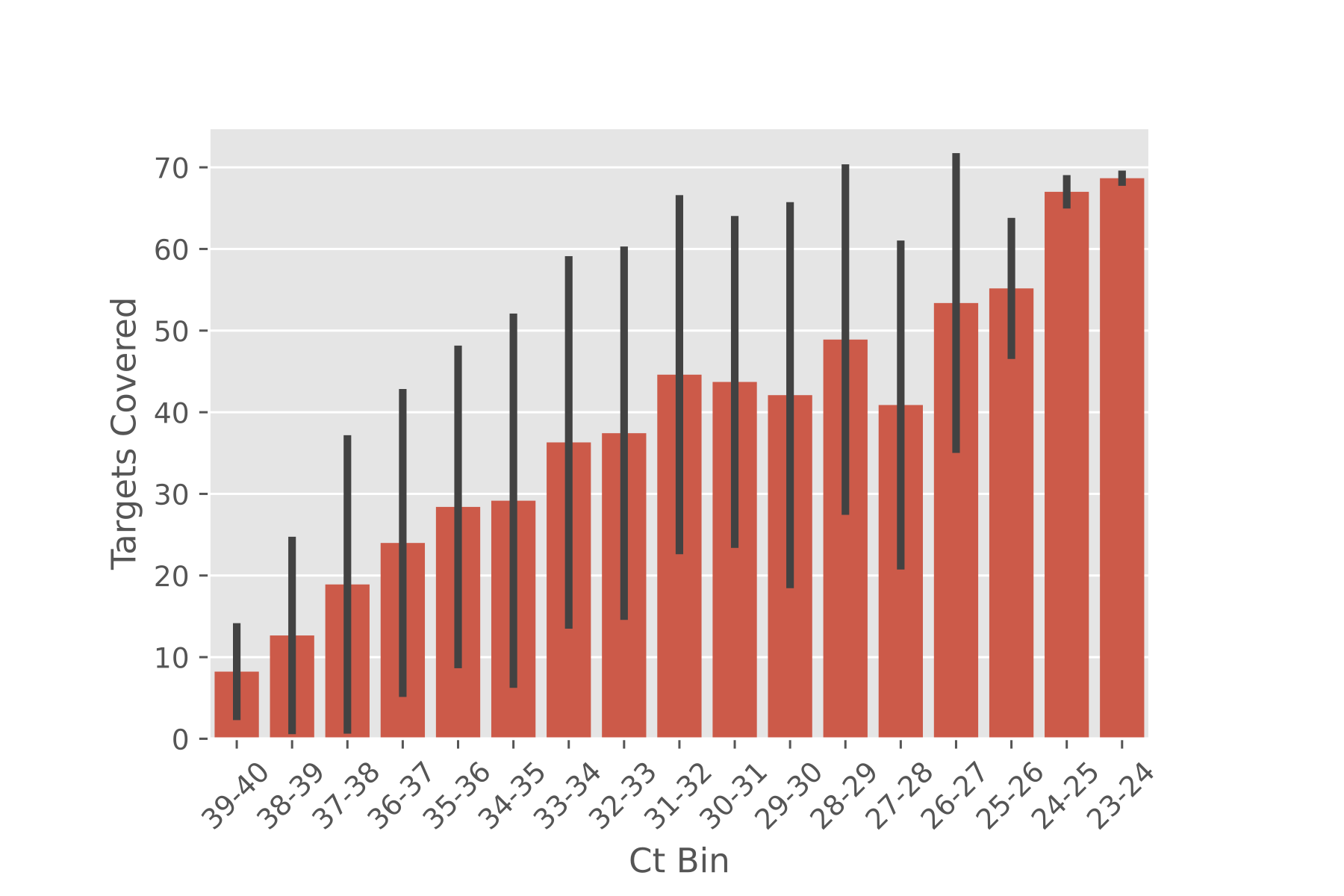
***

### ***Supplementary Figure 5. MIP coveraged compared to qPCR quantification.***

The amount of MIP targets covered correlated with the abundance of P. falciparum DNA as quantified by qPCR. Samples with high levels of parasite DNA (low Ct values) demonstrated good coverage while those with lower amounts demonstrated decreased coverage in the single pass sequencing performed on the samples. More variable coverage was consequence of imperfect balancing of each individual library. Based on redundancy of the molecular barcodes additional sequencing would have yield coverage on par with control sequences.
