# Supplementary material for: Drug-Resistance and Population Structure of Plasmodium falciparum Across the Democratic Republic of Congo Using High-Throughput Molecular Inversion Probes
Source: J Infect Dis. 2018 Apr 28;218(6):946–55. doi: 10.1093/infdis/jiy223 (PMC6093412; doi:10.1093/infdis/jiy223)
Supplement: Supplementary Figure6 [file jiy223_suppl_supplementary_figure6.docx]

###

| **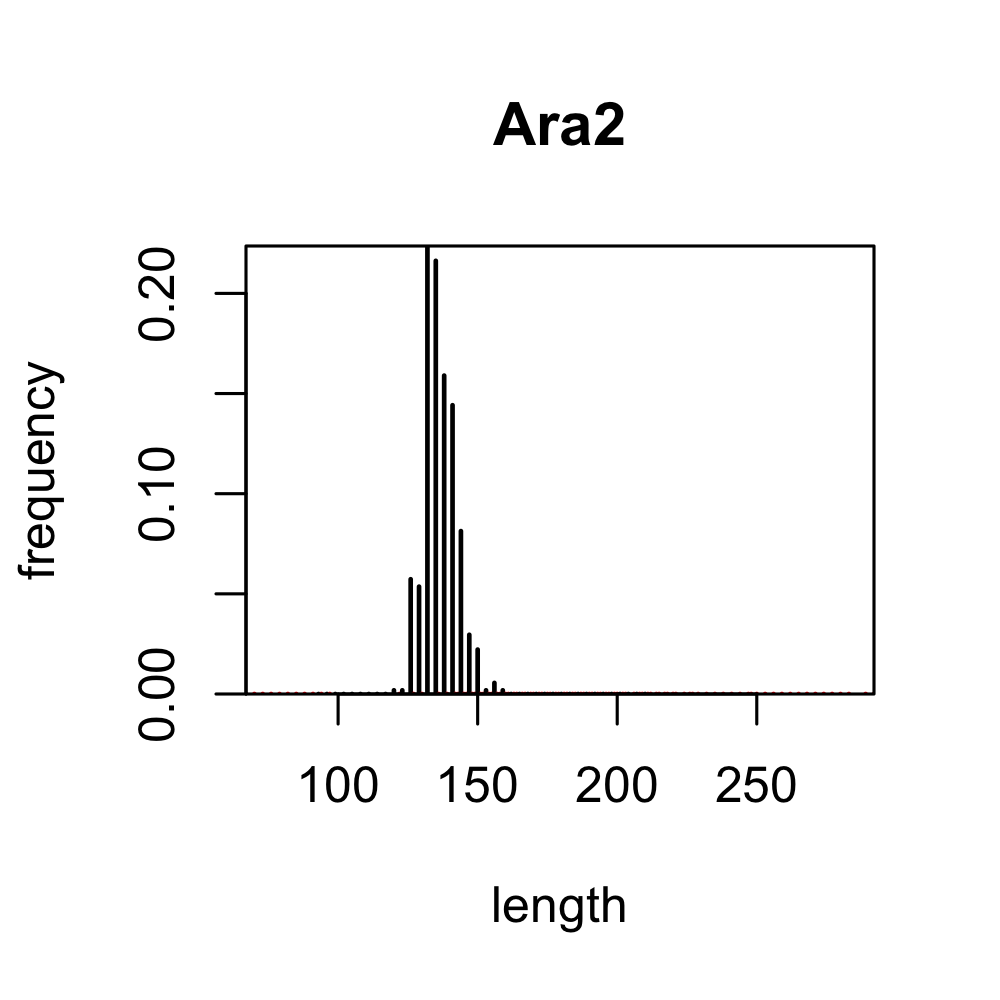** | **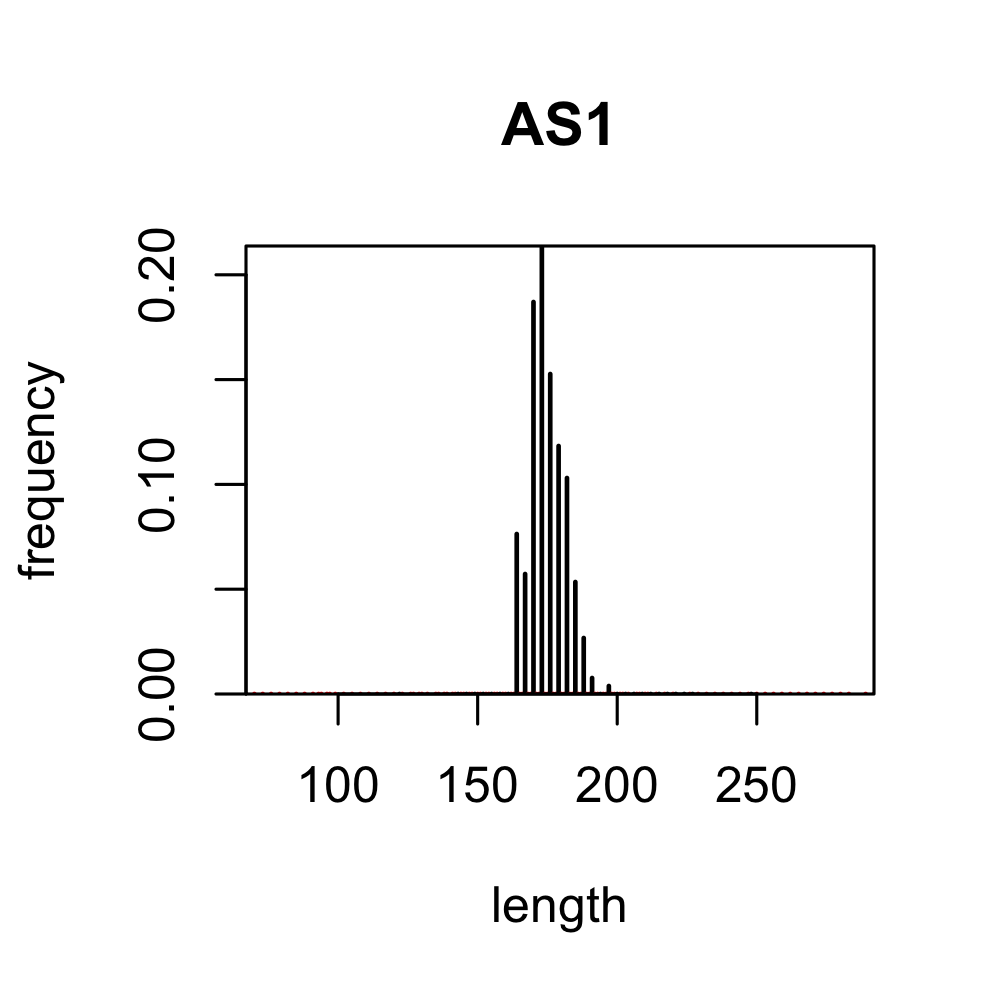** | **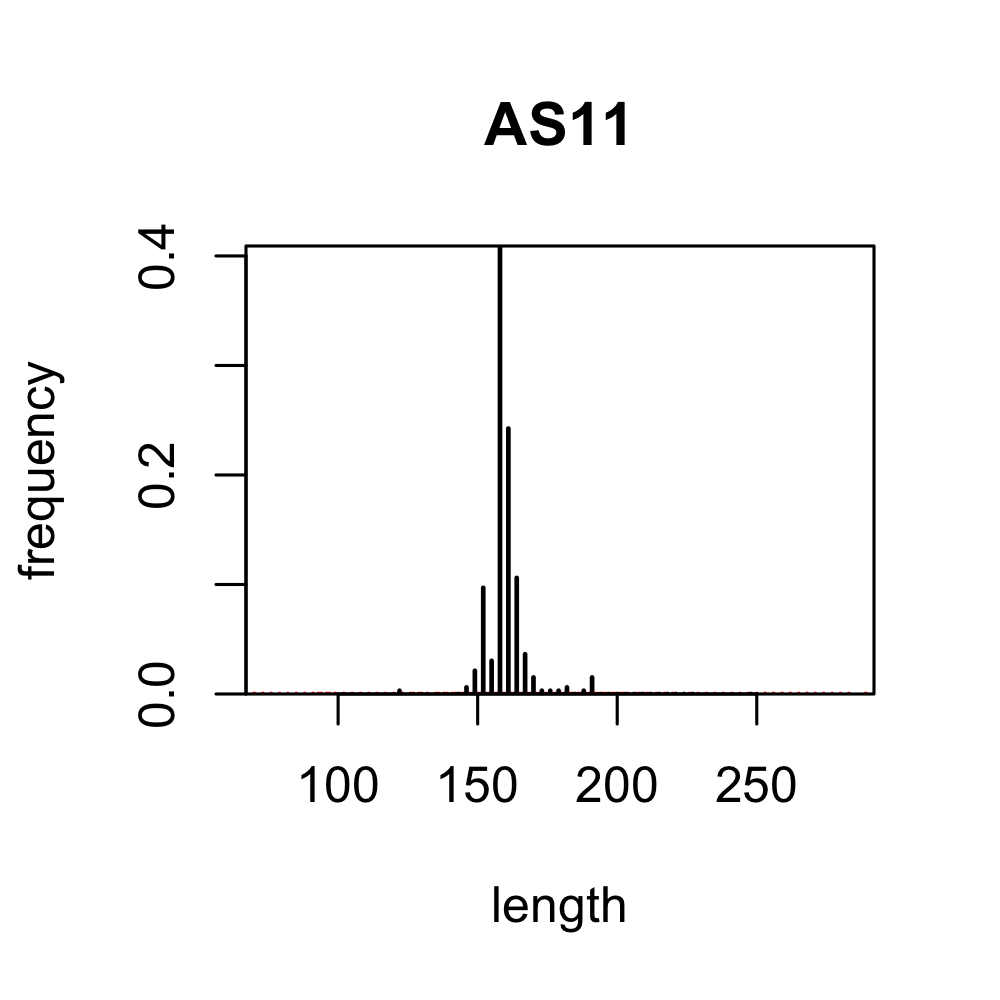** | **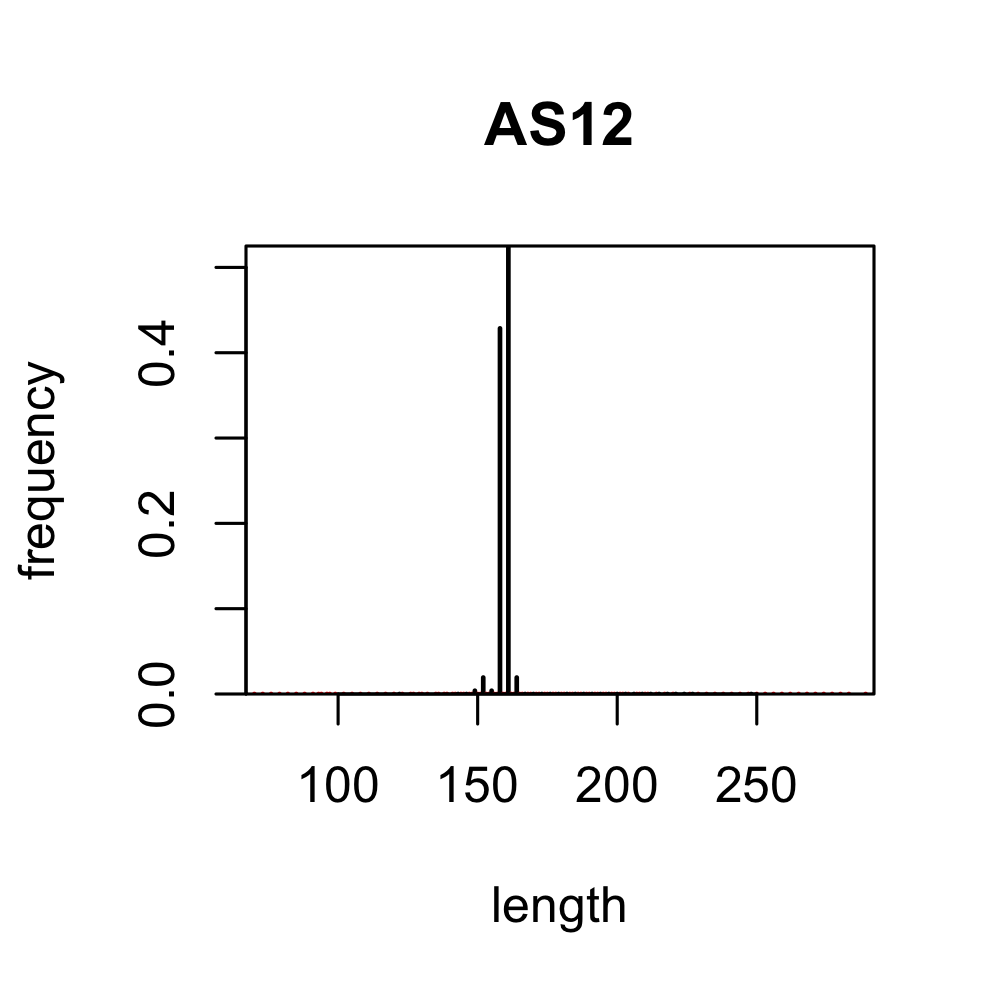** |
| --- | --- | --- | --- |
| **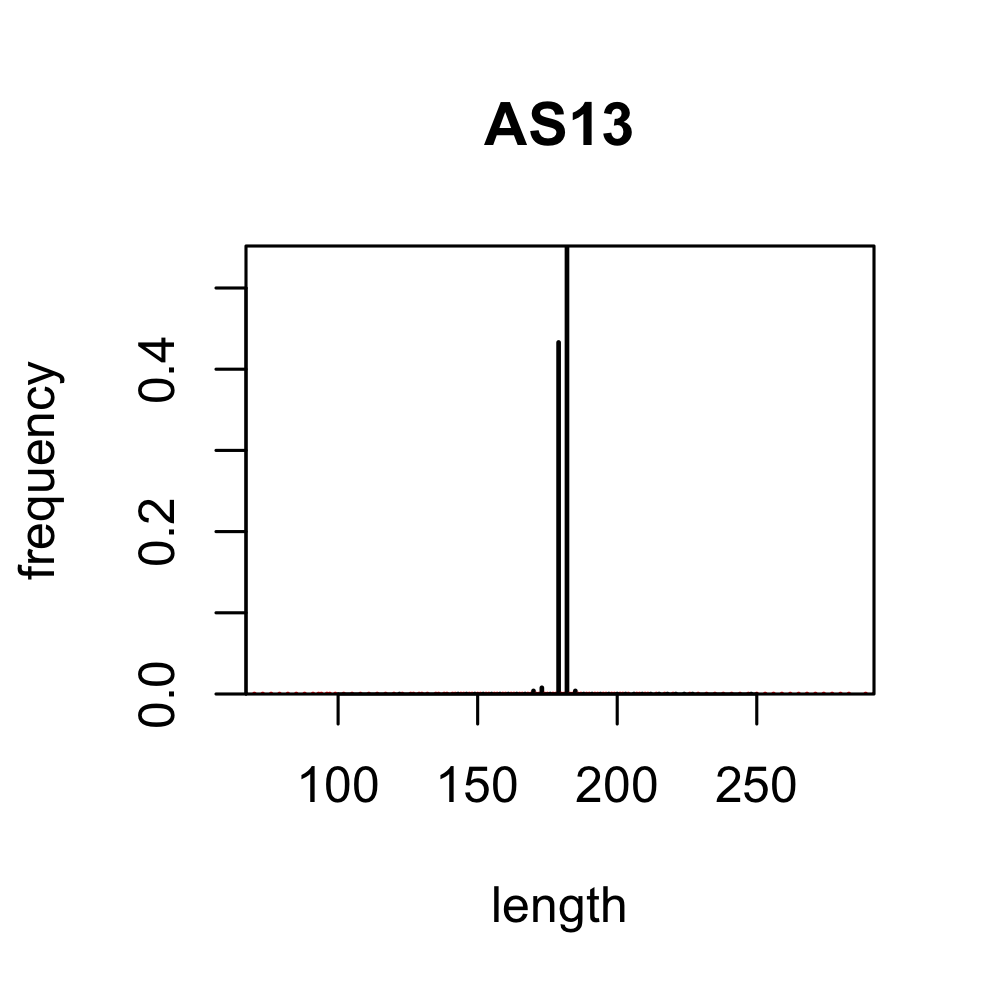** | **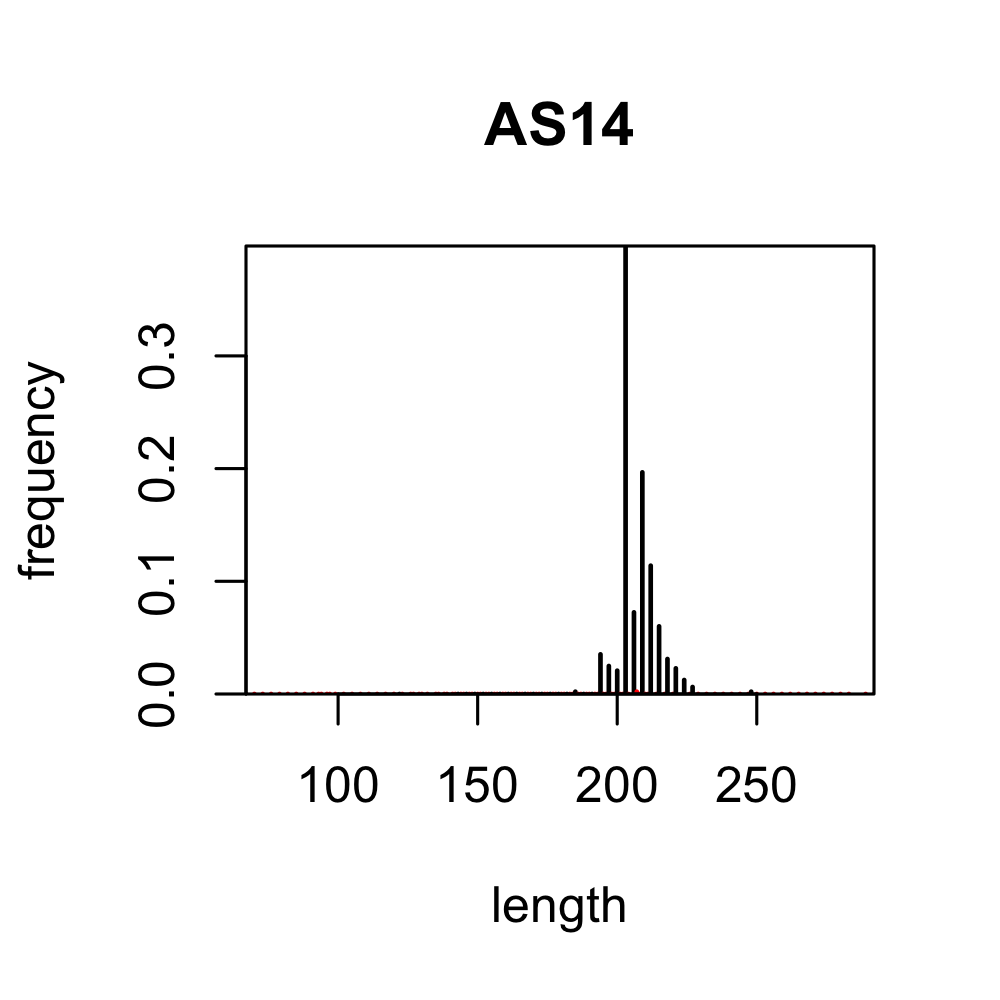** | **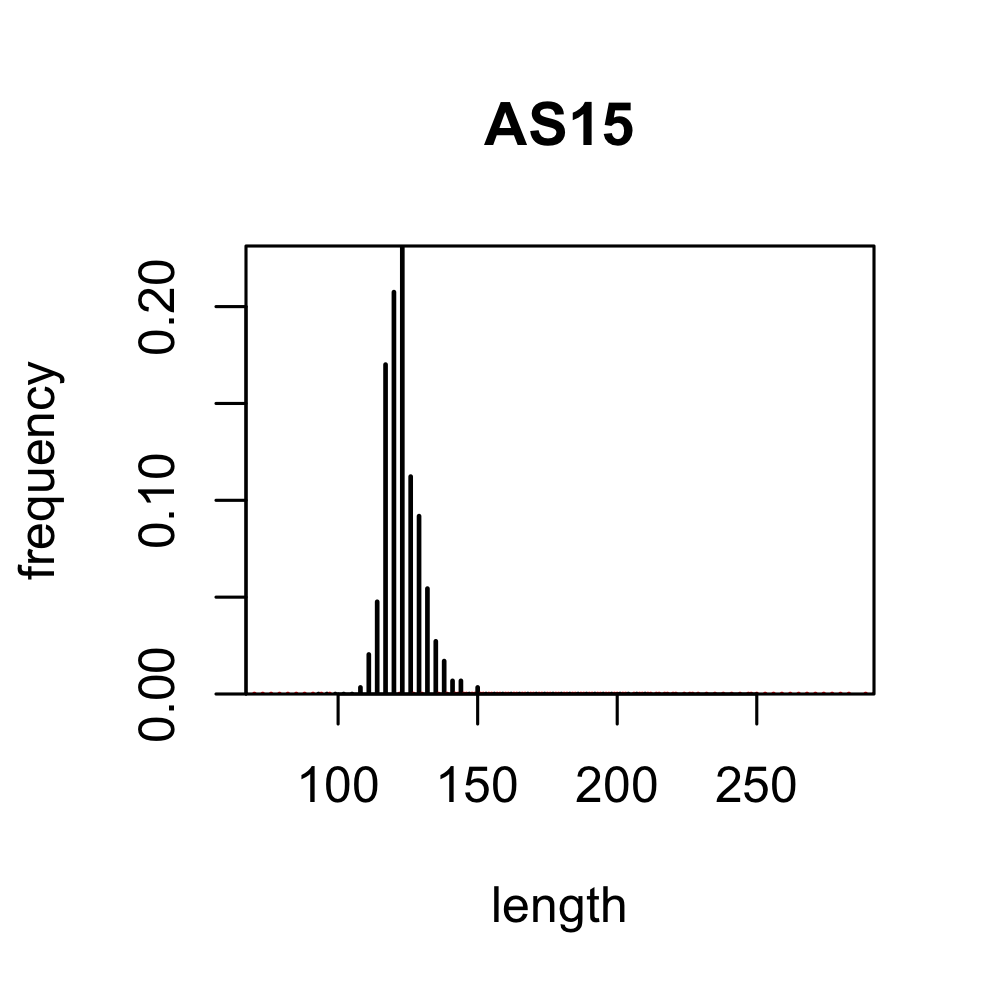** | **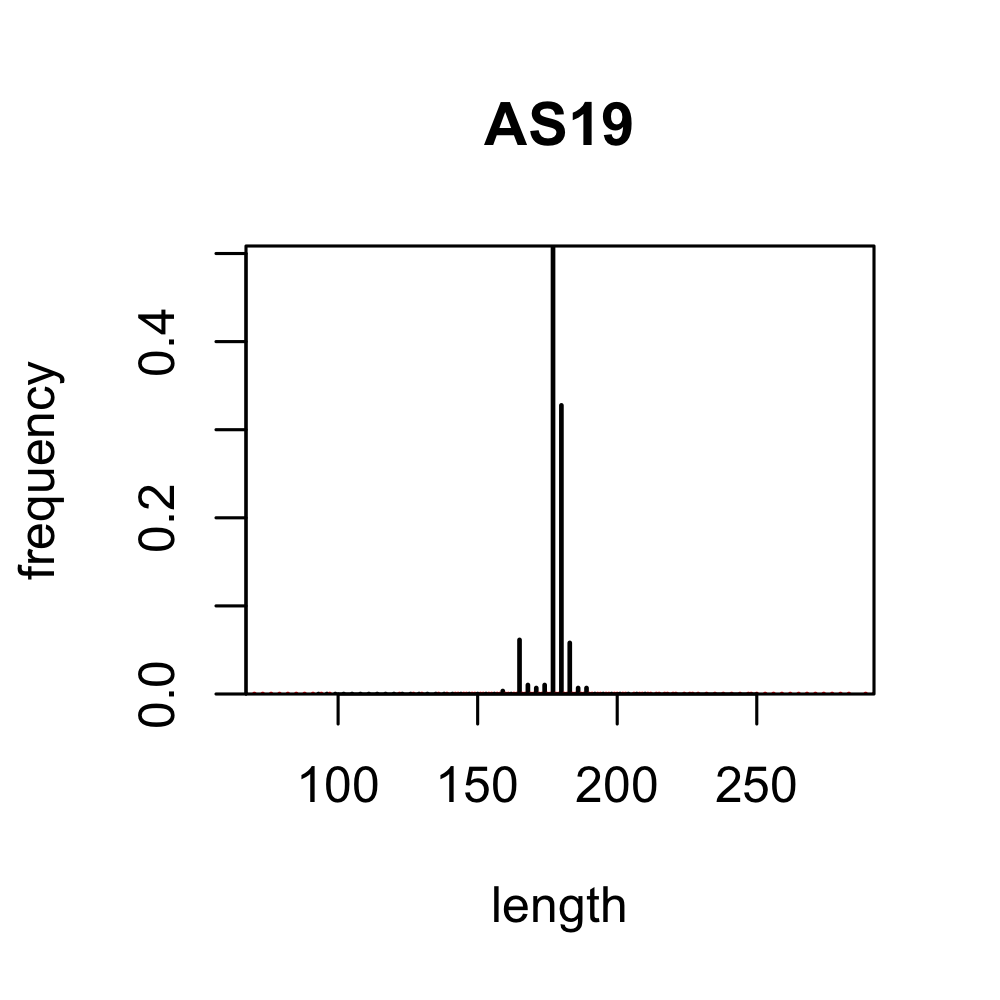** |
| **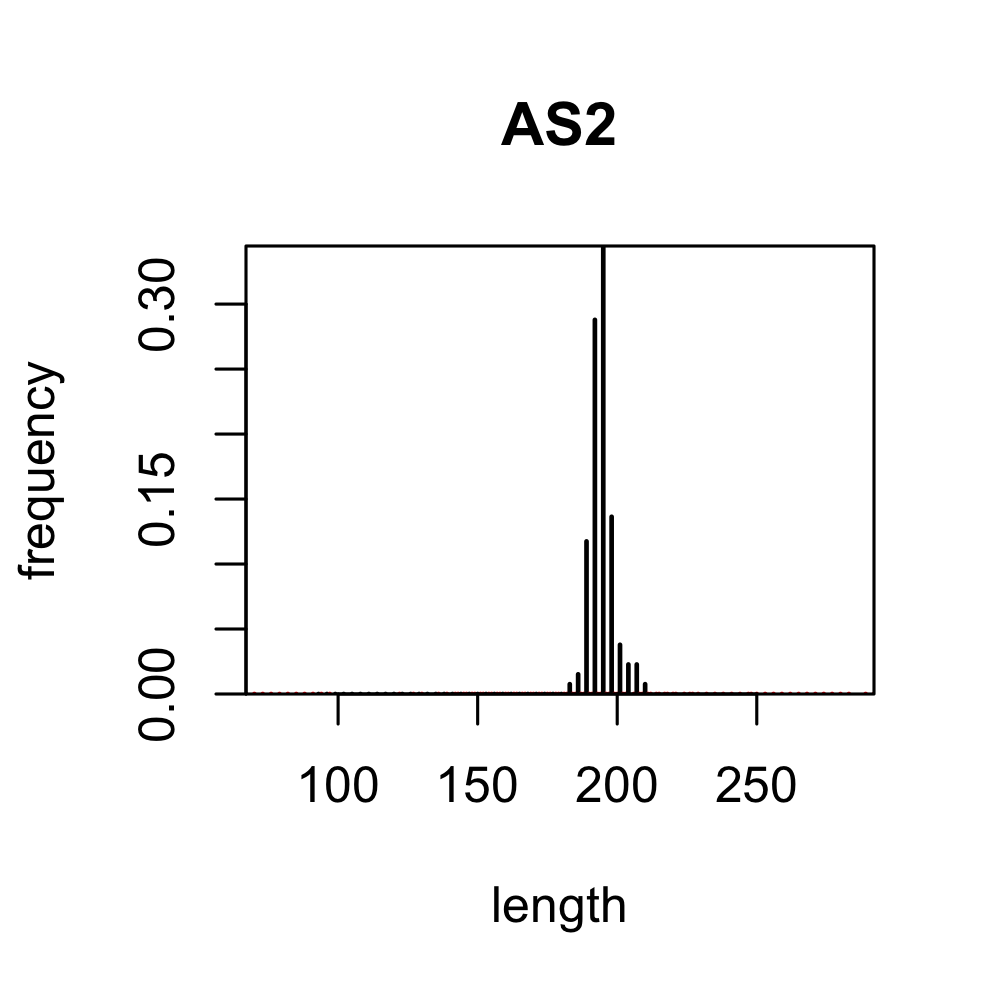** | **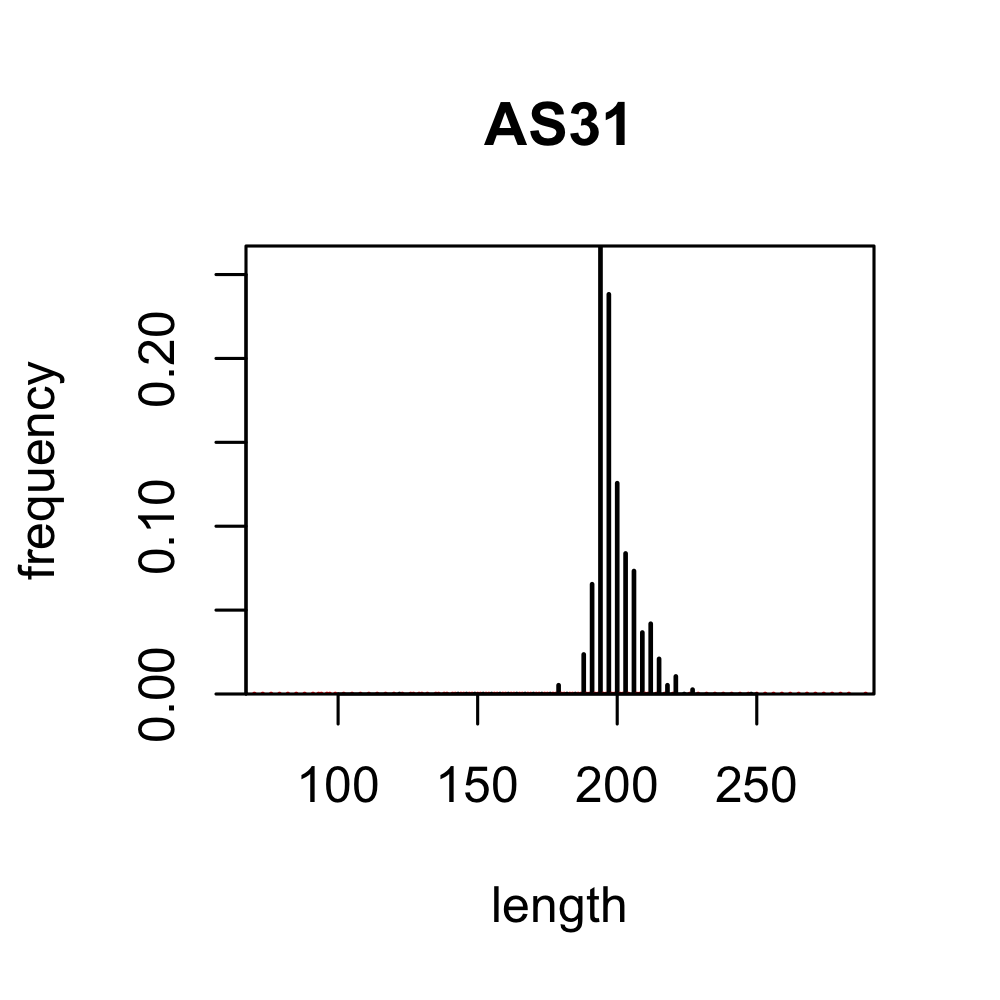** | **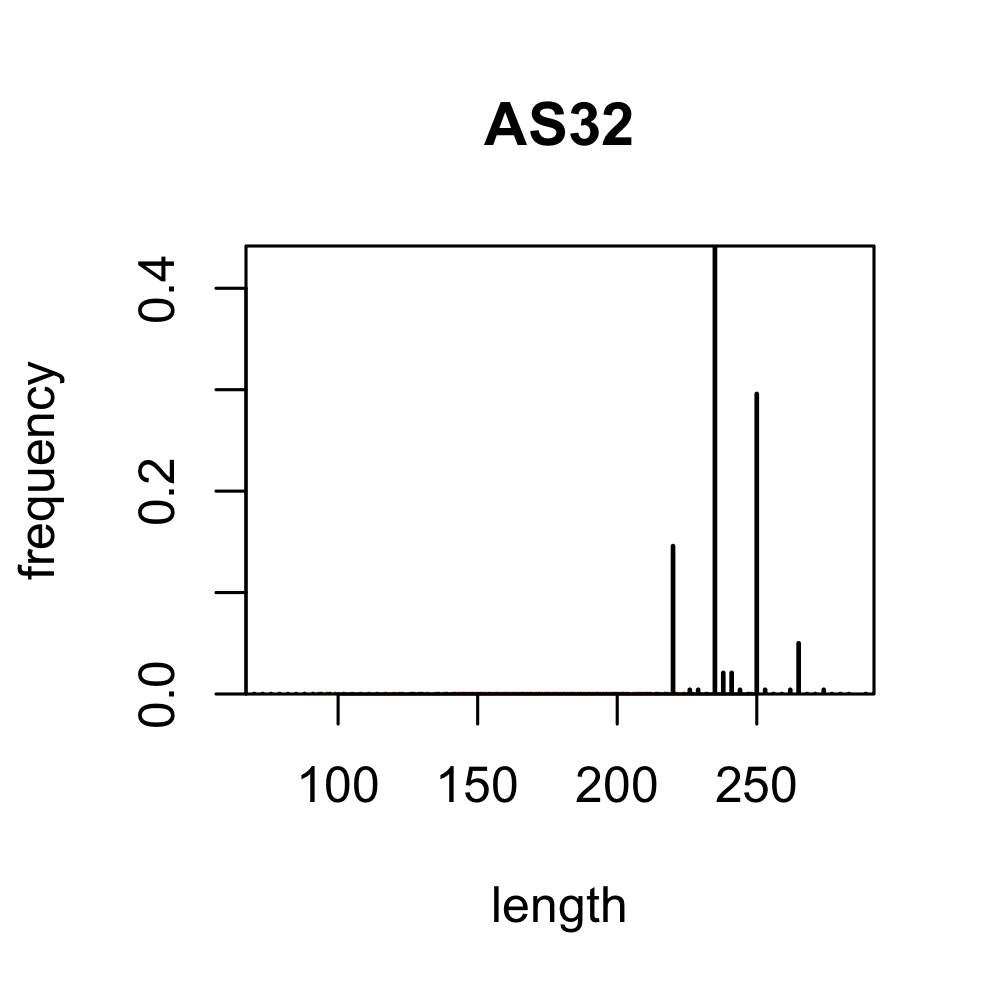** | **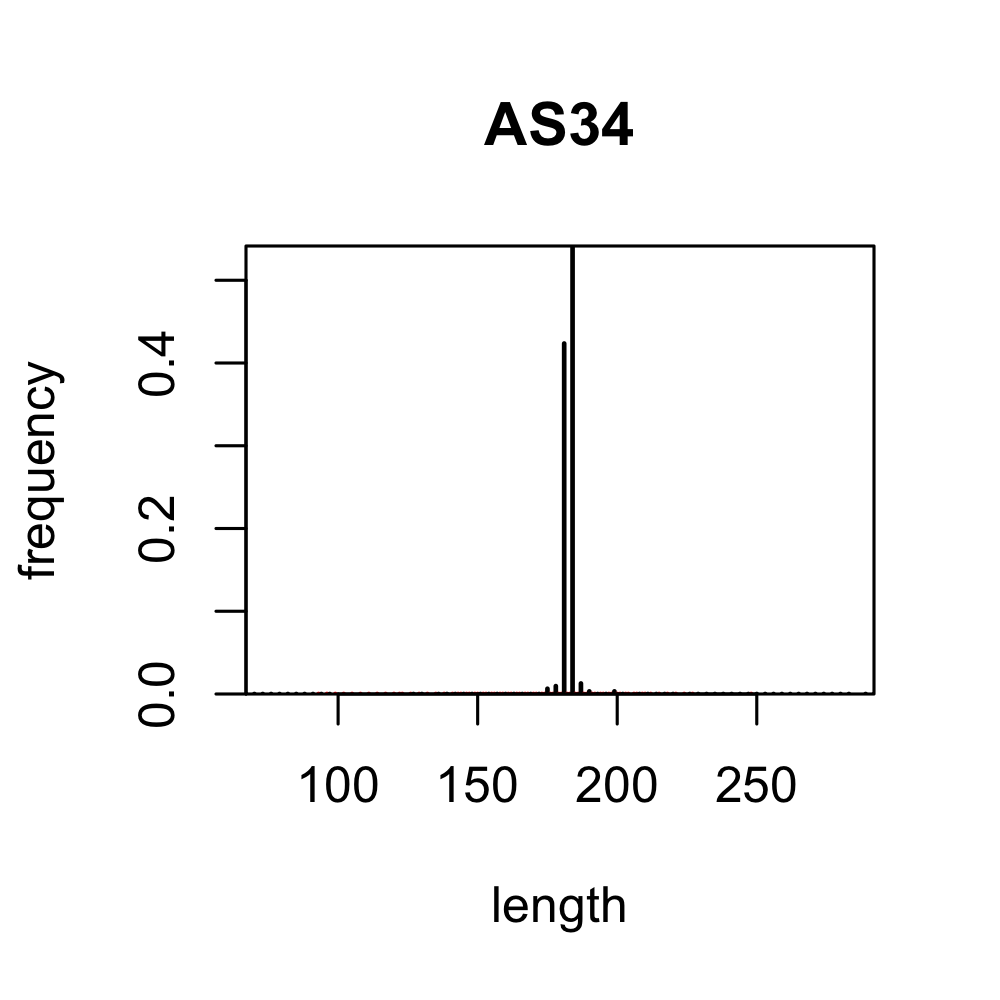** |
| **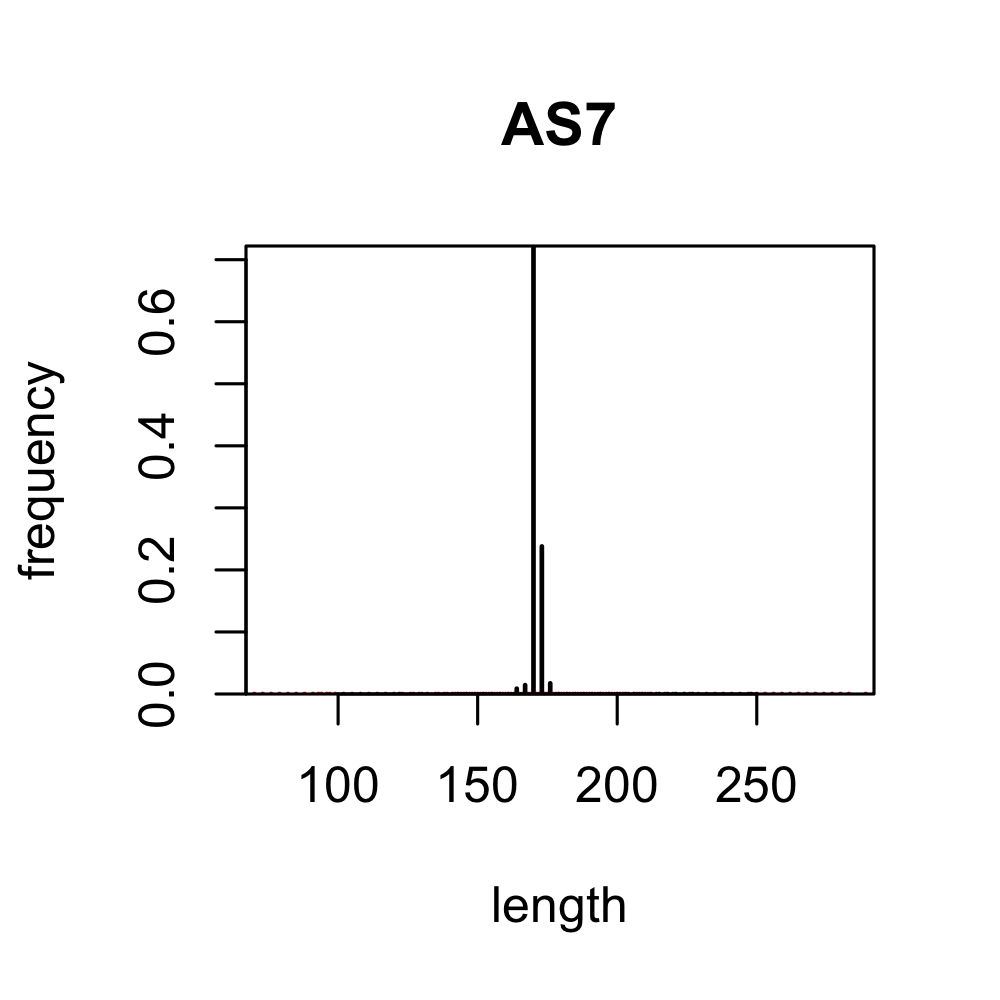** | **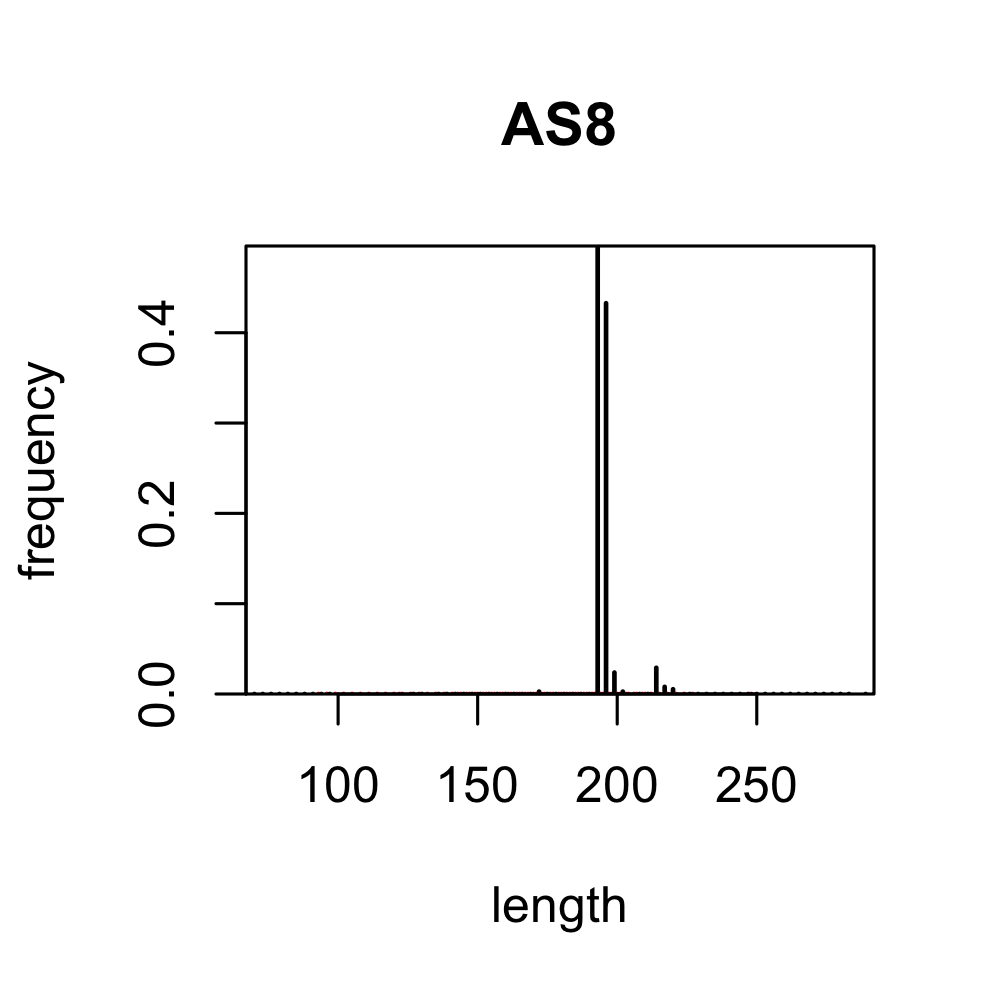** | **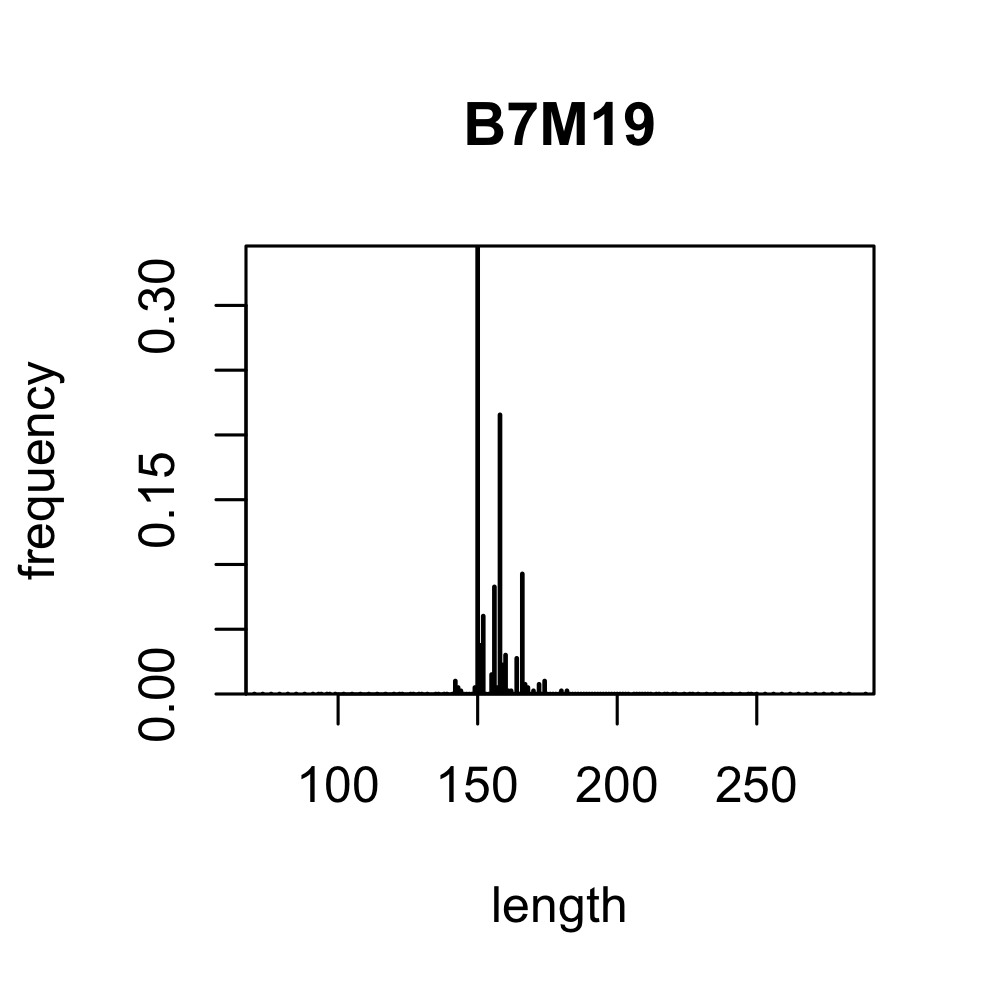** | **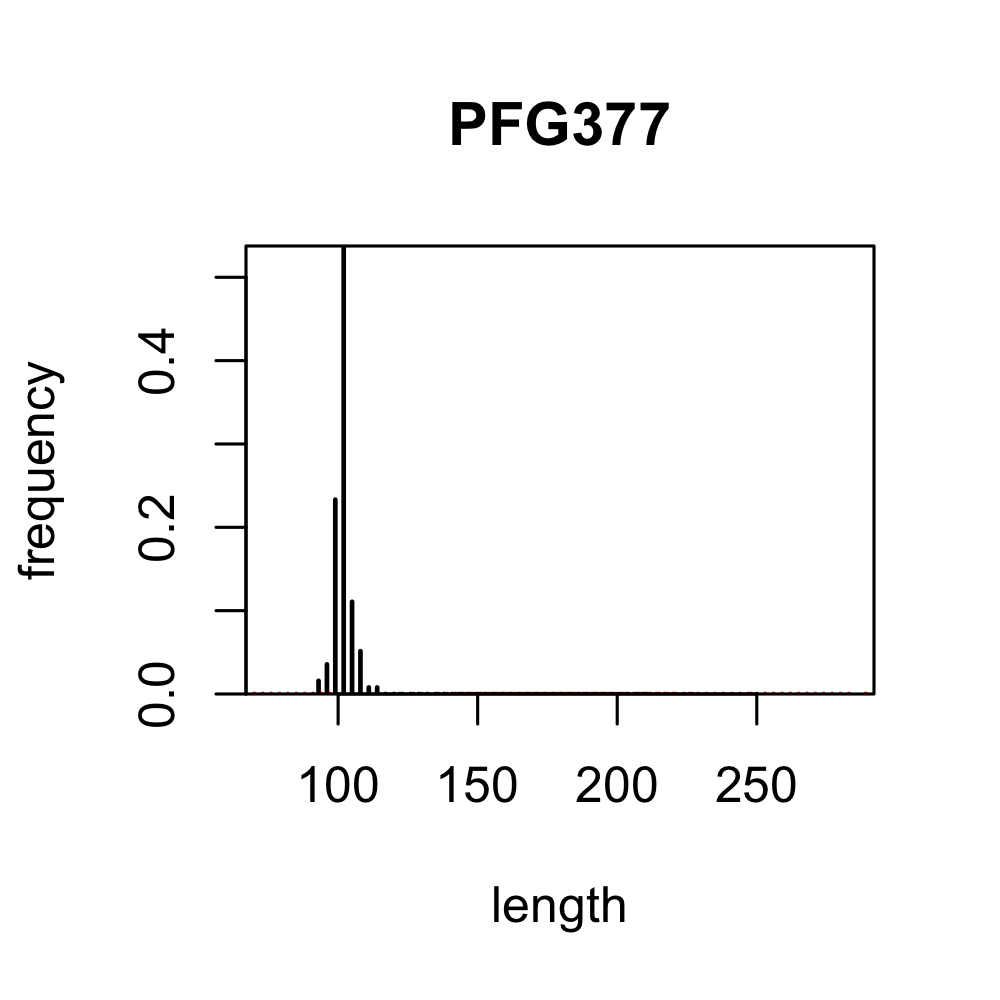** |
| **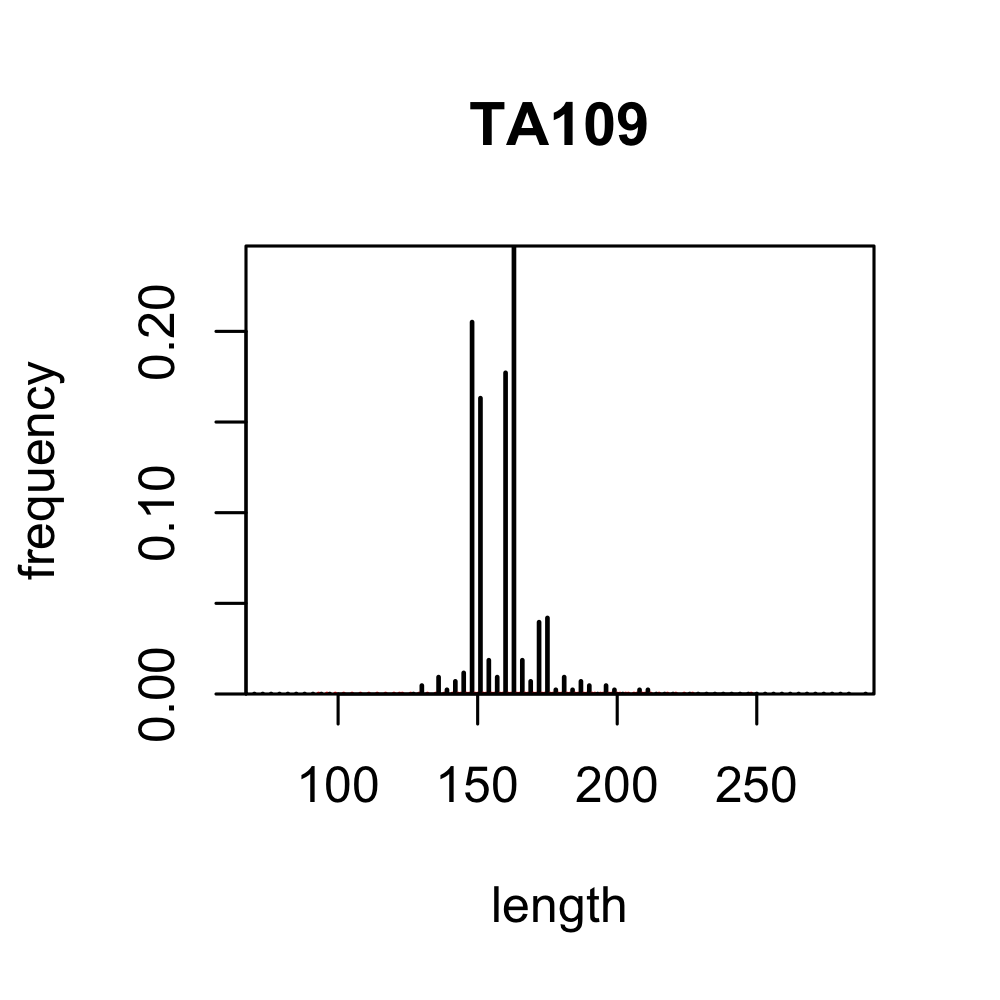** | **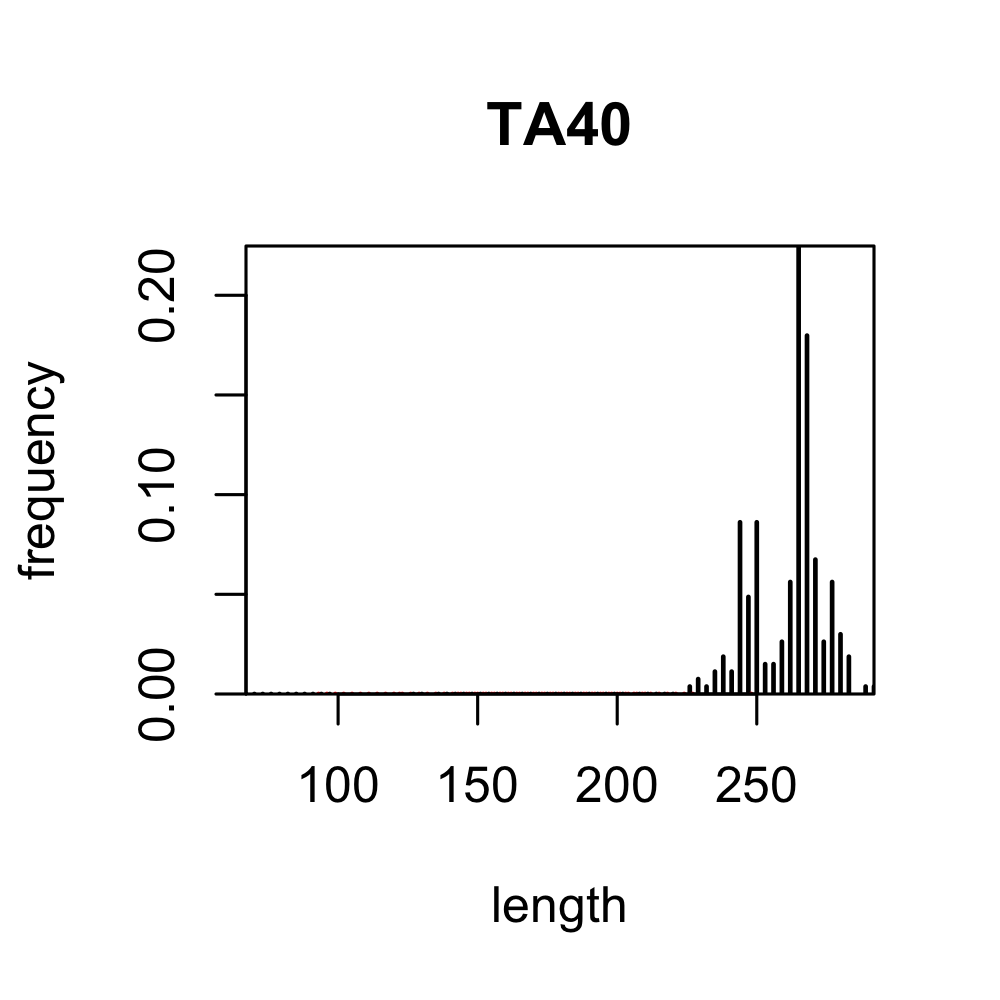** | **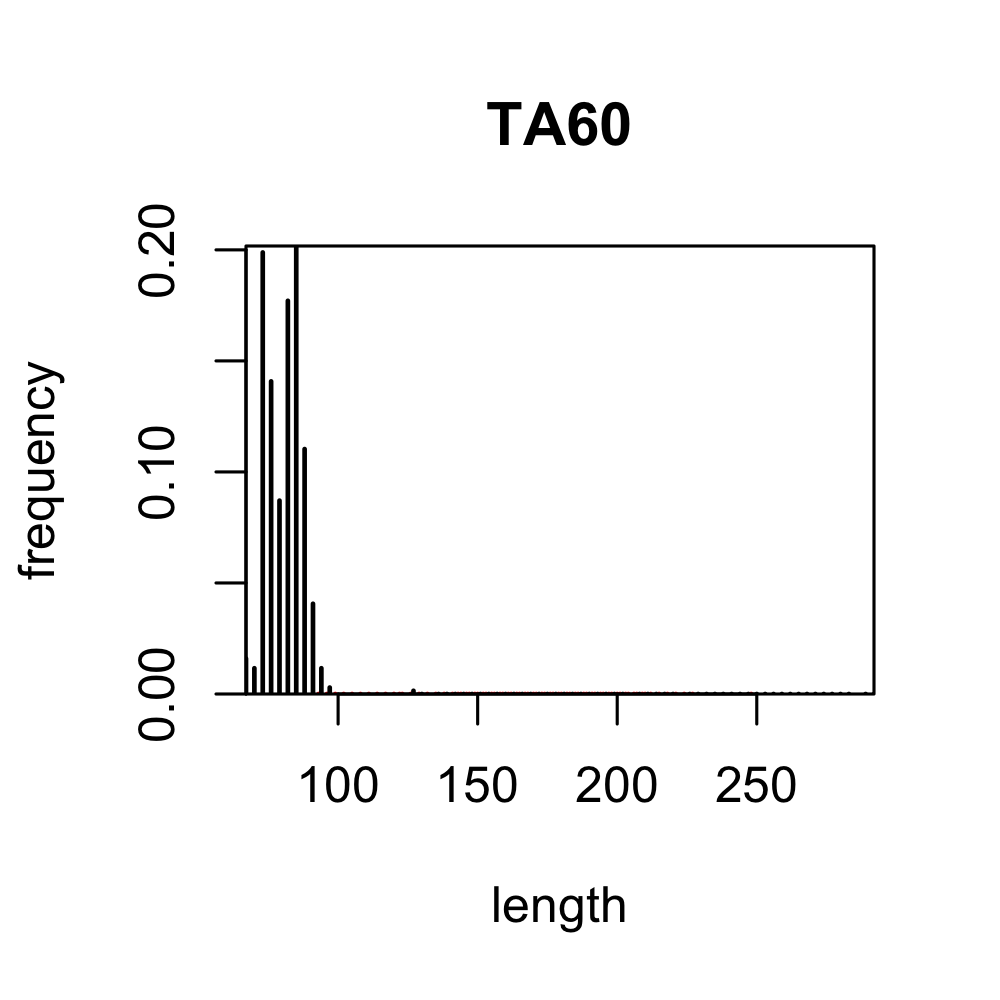** | **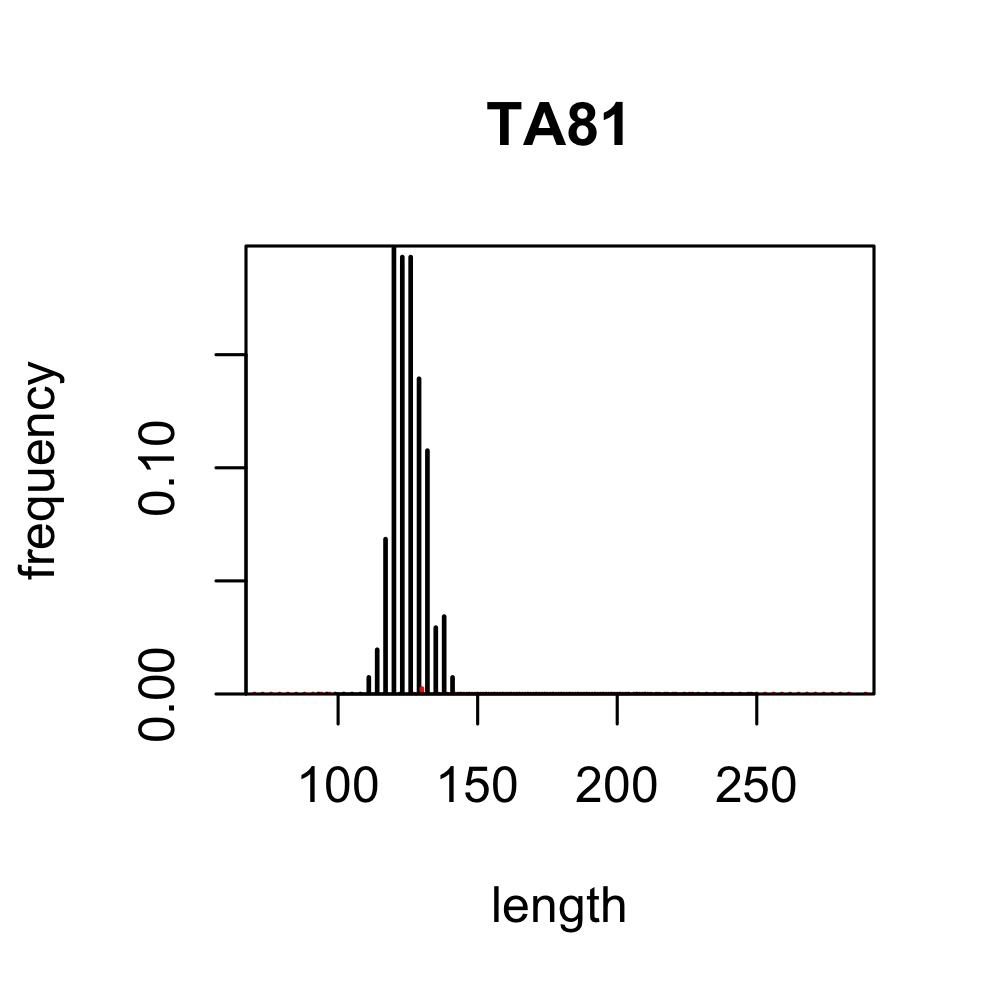** |

### ***Supplementary Figure 6. Microsatellite length distributions for the 20 well-validated microsatellites.***

Histograms of microsatellite lengths for the 20 well-validated microsatellites. 19 loci showed clear trinucleotide repeat distributions (all apart from B7M19). Within these 19 trinucleotide loci, 2 length calls fell outside the trinucleotide pattern and were removed - one in AS14 and one in TA81. These are indicated in red.
