# Supplementary material for: Drug-Resistance and Population Structure of Plasmodium falciparum Across the Democratic Republic of Congo Using High-Throughput Molecular Inversion Probes
Source: J Infect Dis. 2018 Apr 28;218(6):946–55. doi: 10.1093/infdis/jiy223 (PMC6093412; doi:10.1093/infdis/jiy223)
Supplement: Supplementary Figure7 [file jiy223_suppl_supplementary_figure7.docx]

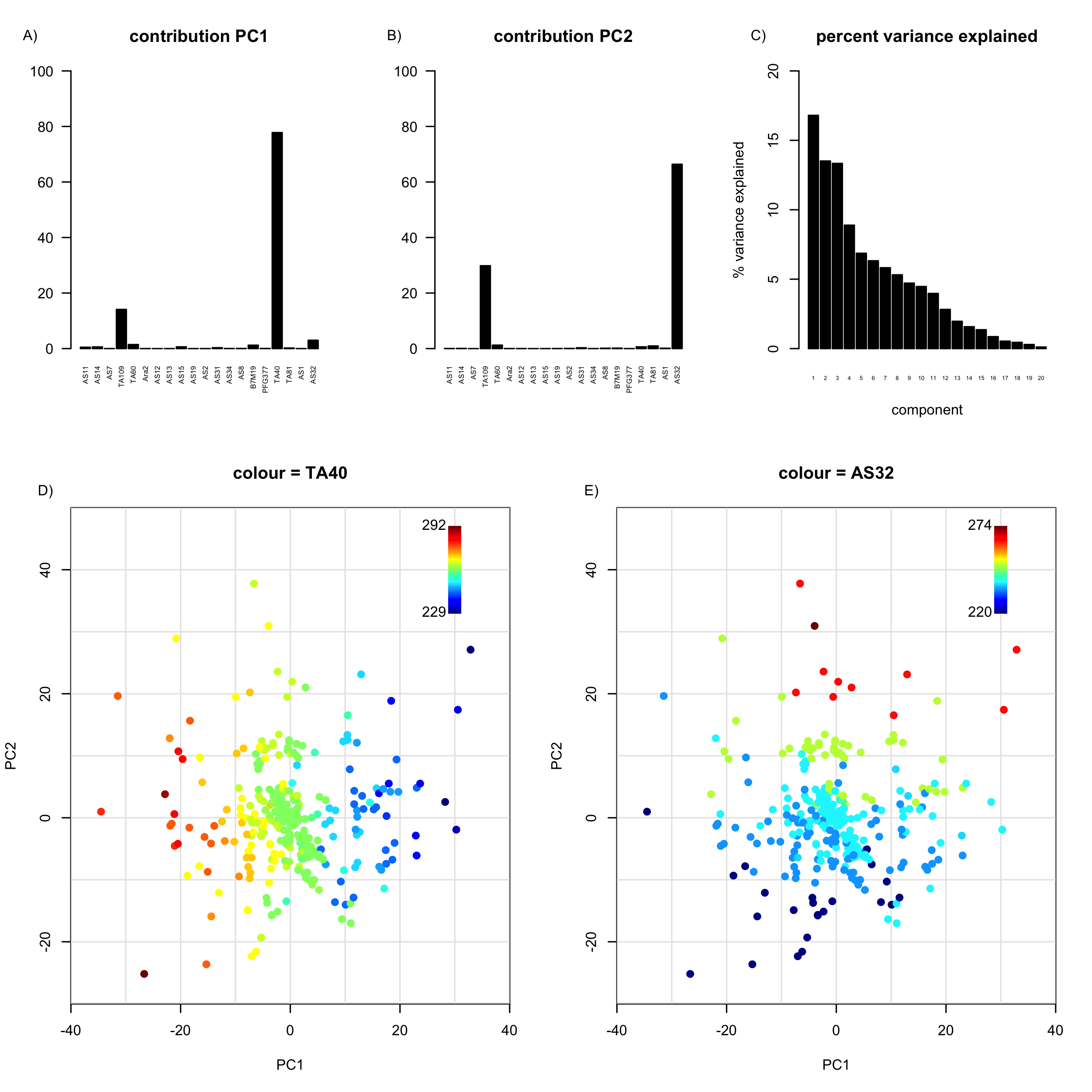


### ***Supplementary Figure 7. PCA Contribution and Variation.***

Initial principal components are mainly driven by singular microsatellites, consistent with their perceived independence (A,B). This is highlighted by coloring based on length of microsatellites TA40 (D) and AS32 (E), which correlate strongly with PC1 and PC2, respectively. The initial components also do not explain the majority of the variation (C).
