# Supplementary material for: Drug-Resistance and Population Structure of Plasmodium falciparum Across the Democratic Republic of Congo Using High-Throughput Molecular Inversion Probes
Source: J Infect Dis. 2018 Apr 28;218(6):946–55. doi: 10.1093/infdis/jiy223 (PMC6093412; doi:10.1093/infdis/jiy223)
Supplement: Supplementary Figure8 [file jiy223_suppl_supplementary_figure8.docx]

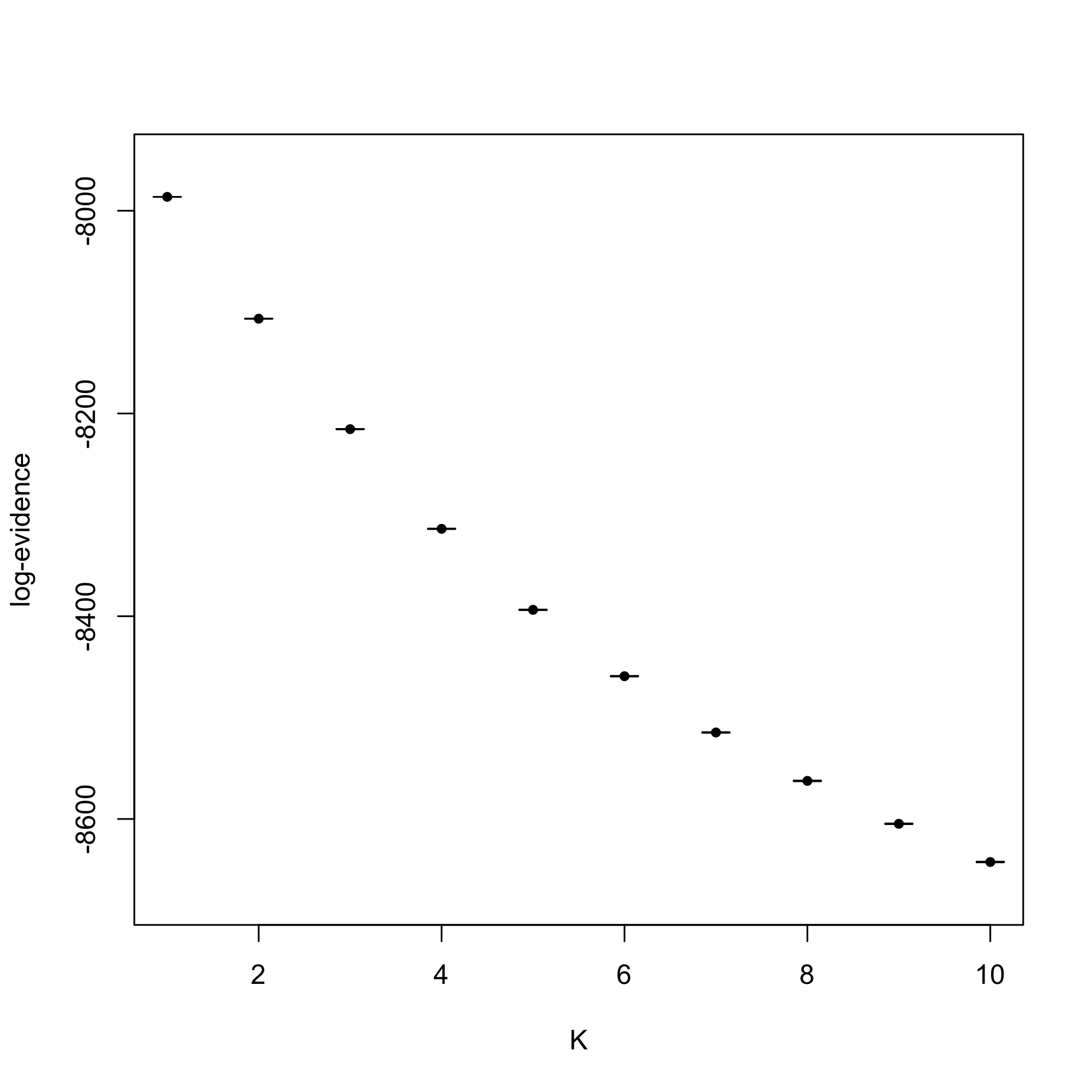


### ***Supplementary Figure 8. Estimate of the number of microsatellite clusters produced by MavericK***

Estimates of the model evidence for each of K=1 to K=10 clusters, generated by the program *MavericK*. Analysis is based on the dominant allele in all individuals, and missing data is accommodated by the program. Estimates are shown on a log scale.
