# Supplementary material for: Drug-Resistance and Population Structure of Plasmodium falciparum Across the Democratic Republic of Congo Using High-Throughput Molecular Inversion Probes
Source: J Infect Dis. 2018 Apr 28;218(6):946–55. doi: 10.1093/infdis/jiy223 (PMC6093412; doi:10.1093/infdis/jiy223)
Supplement: Supplementary Figure9 [file jiy223_suppl_supplementary_figure9.docx]

###

| ***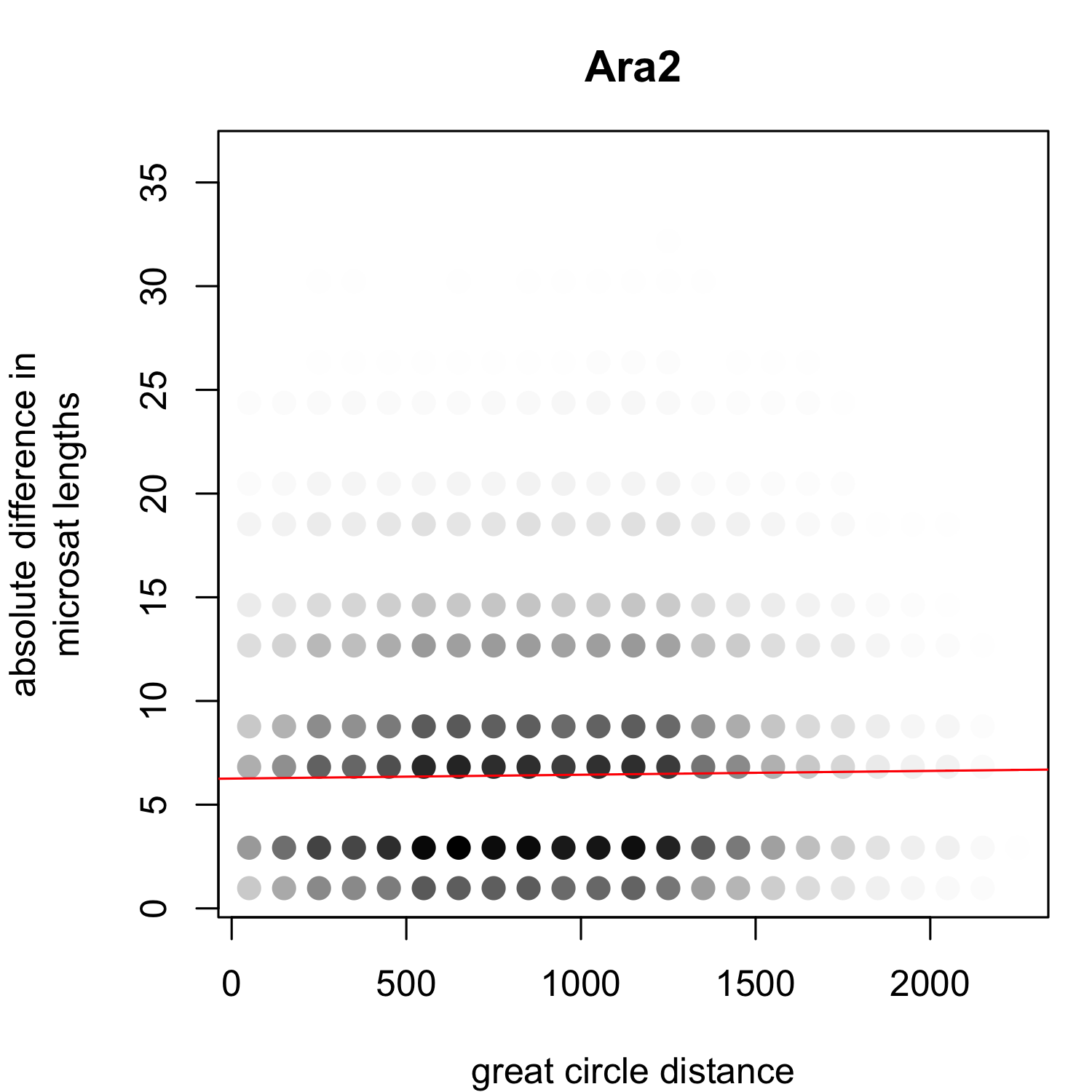*** | ***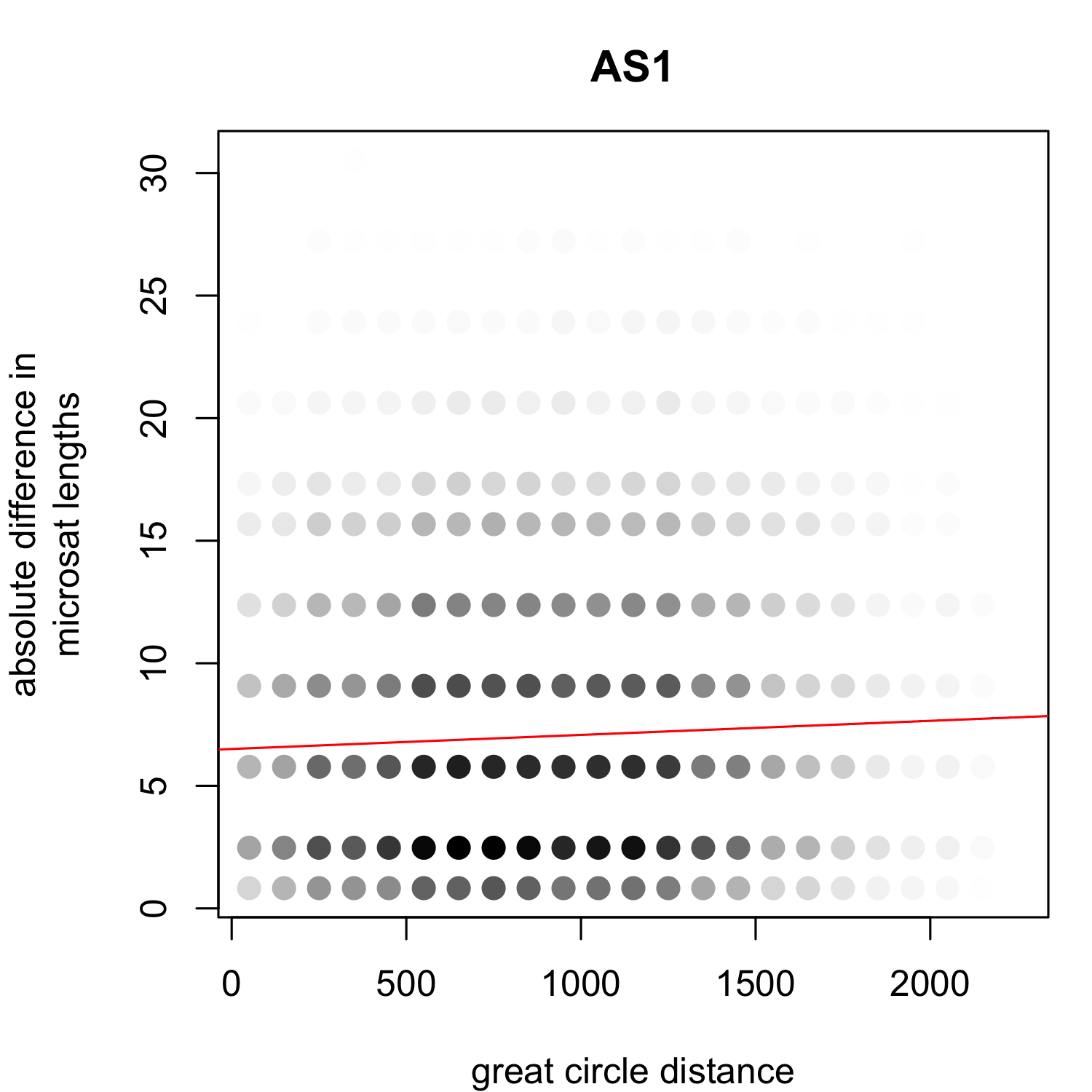*** | ***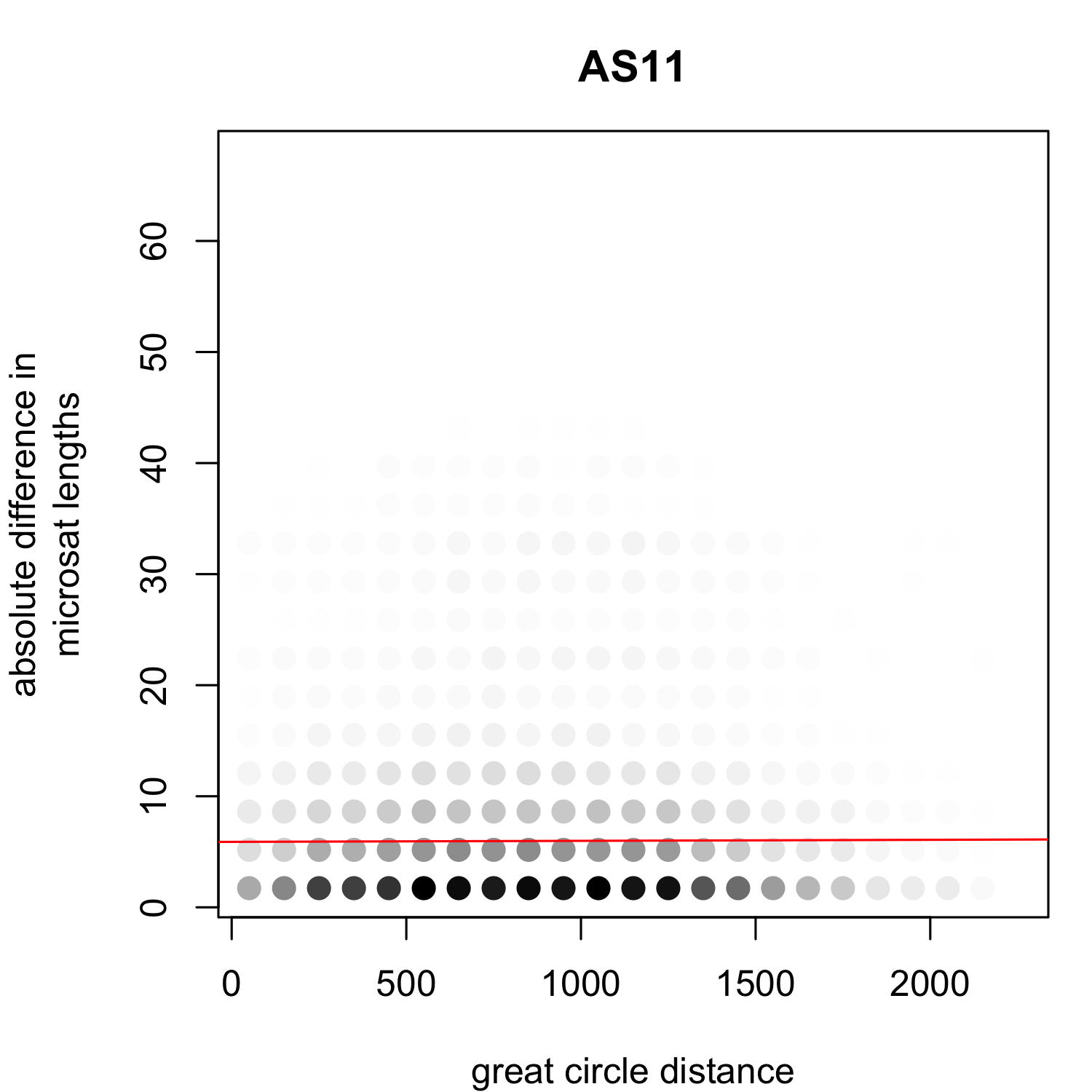*** | ***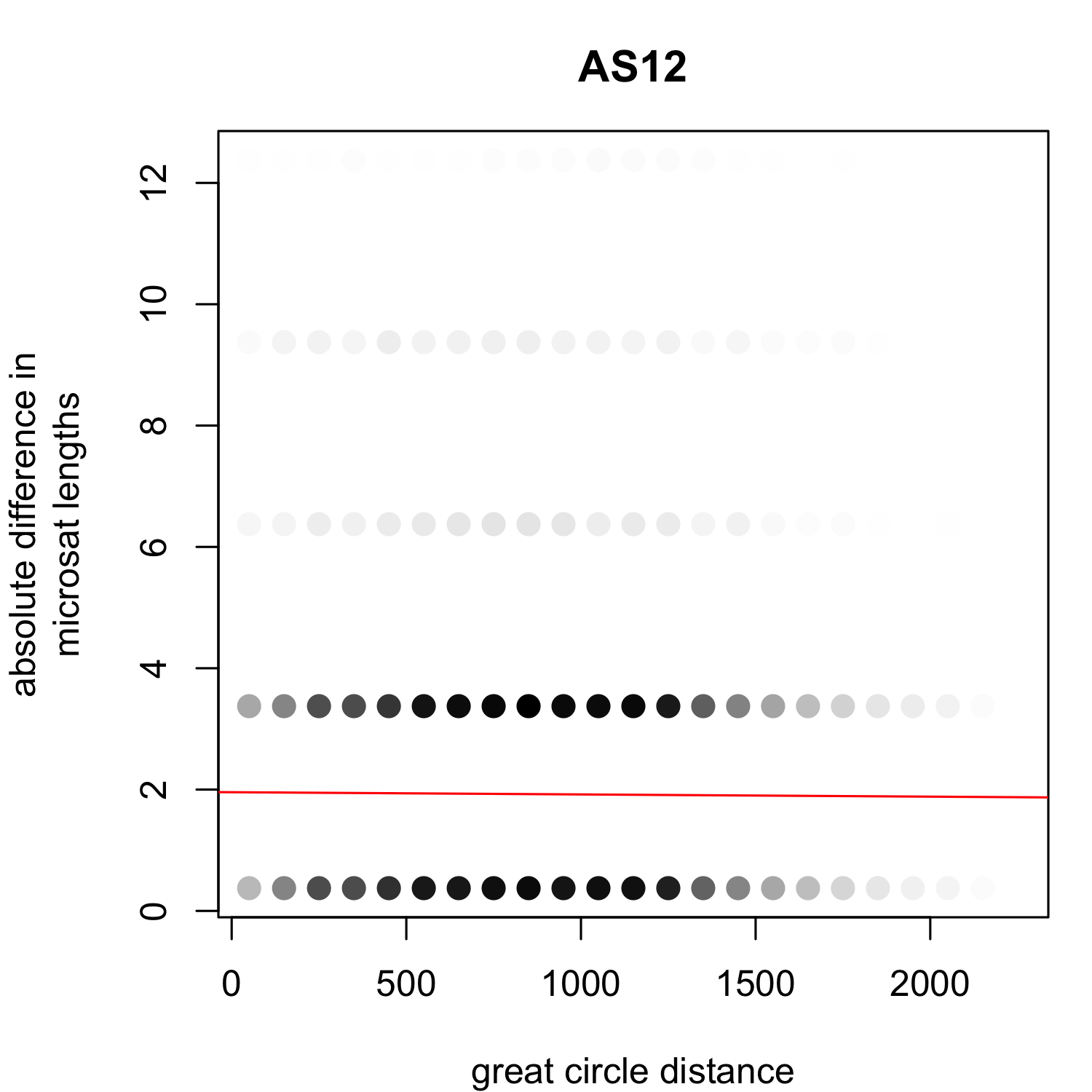*** |
| --- | --- | --- | --- |
| ***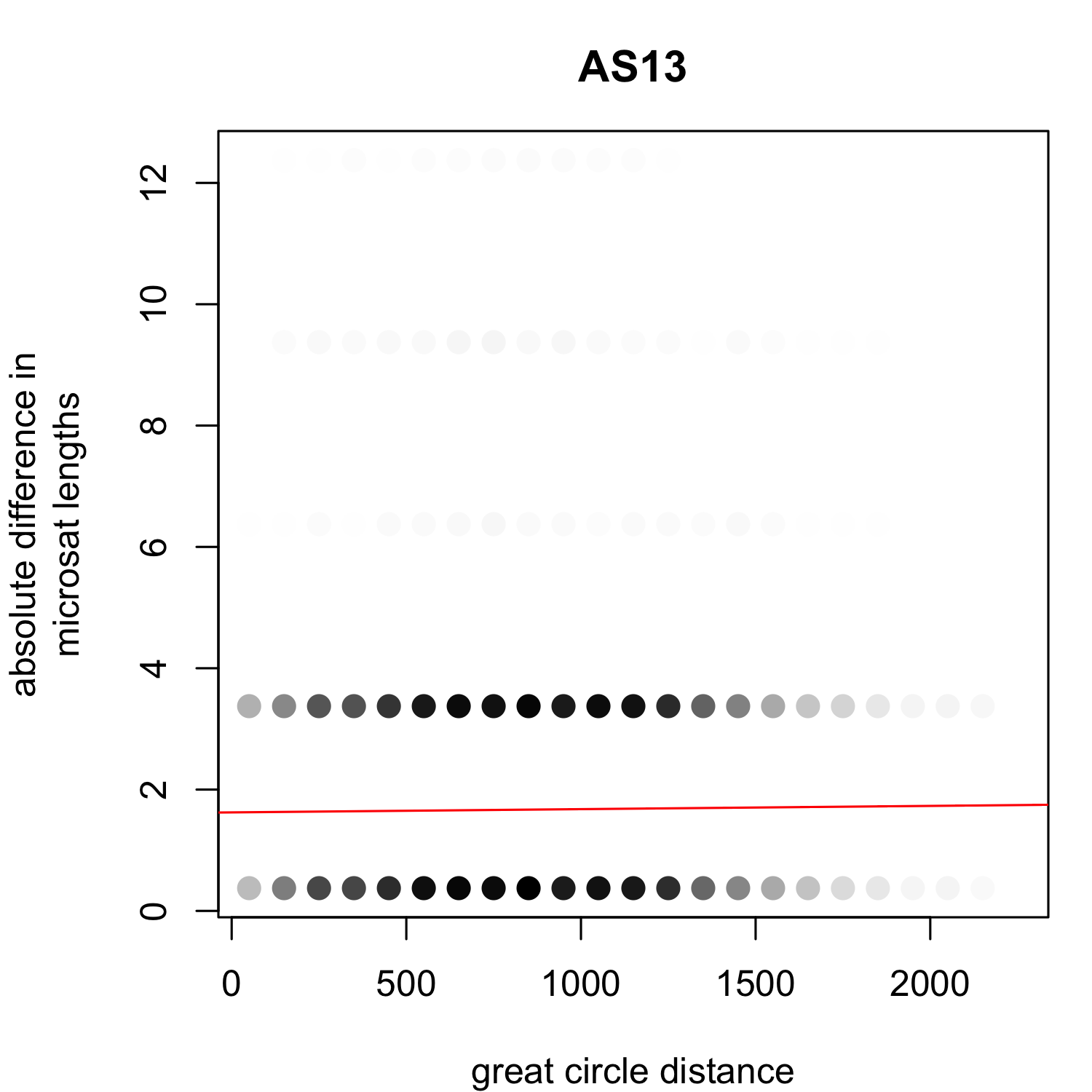*** | ***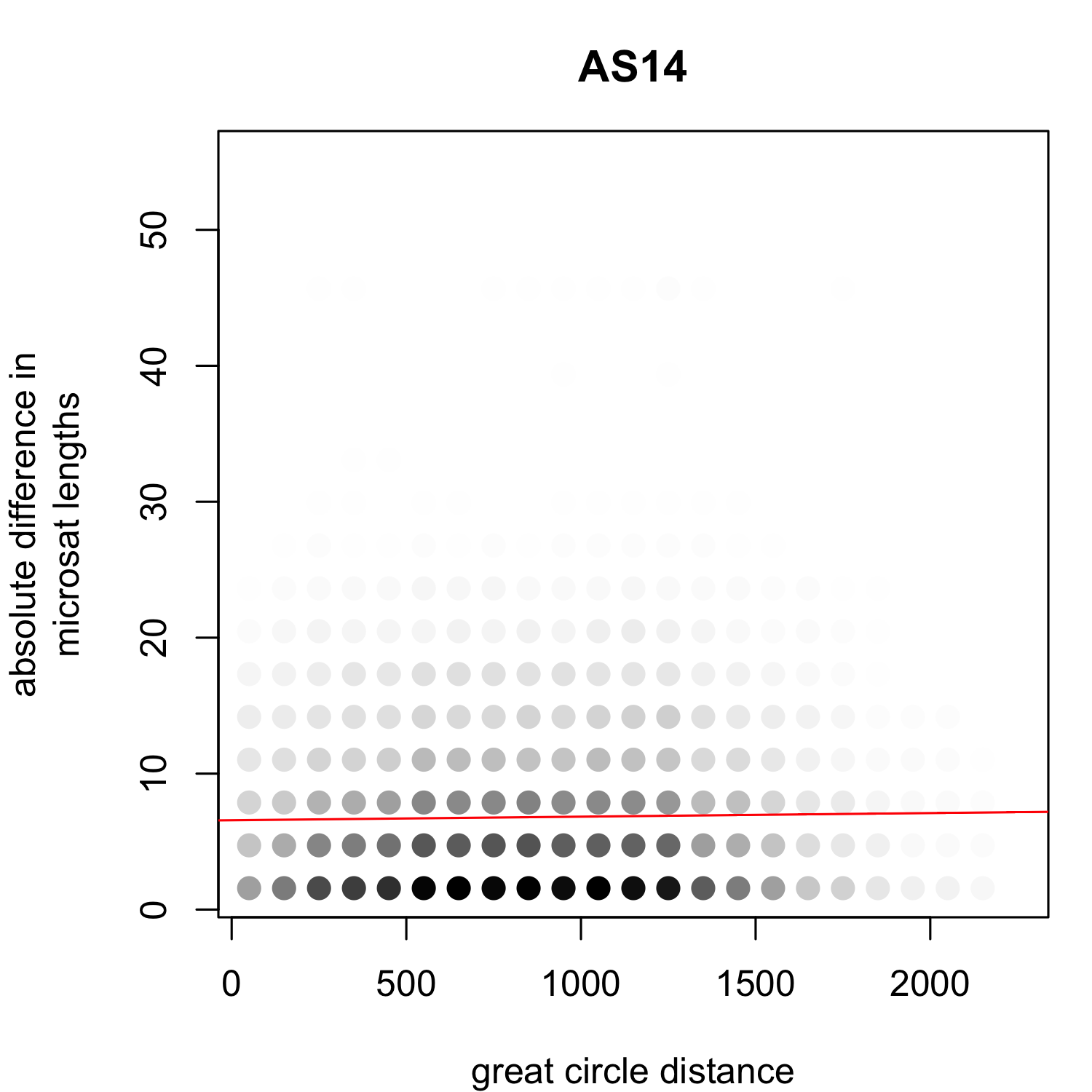*** | ***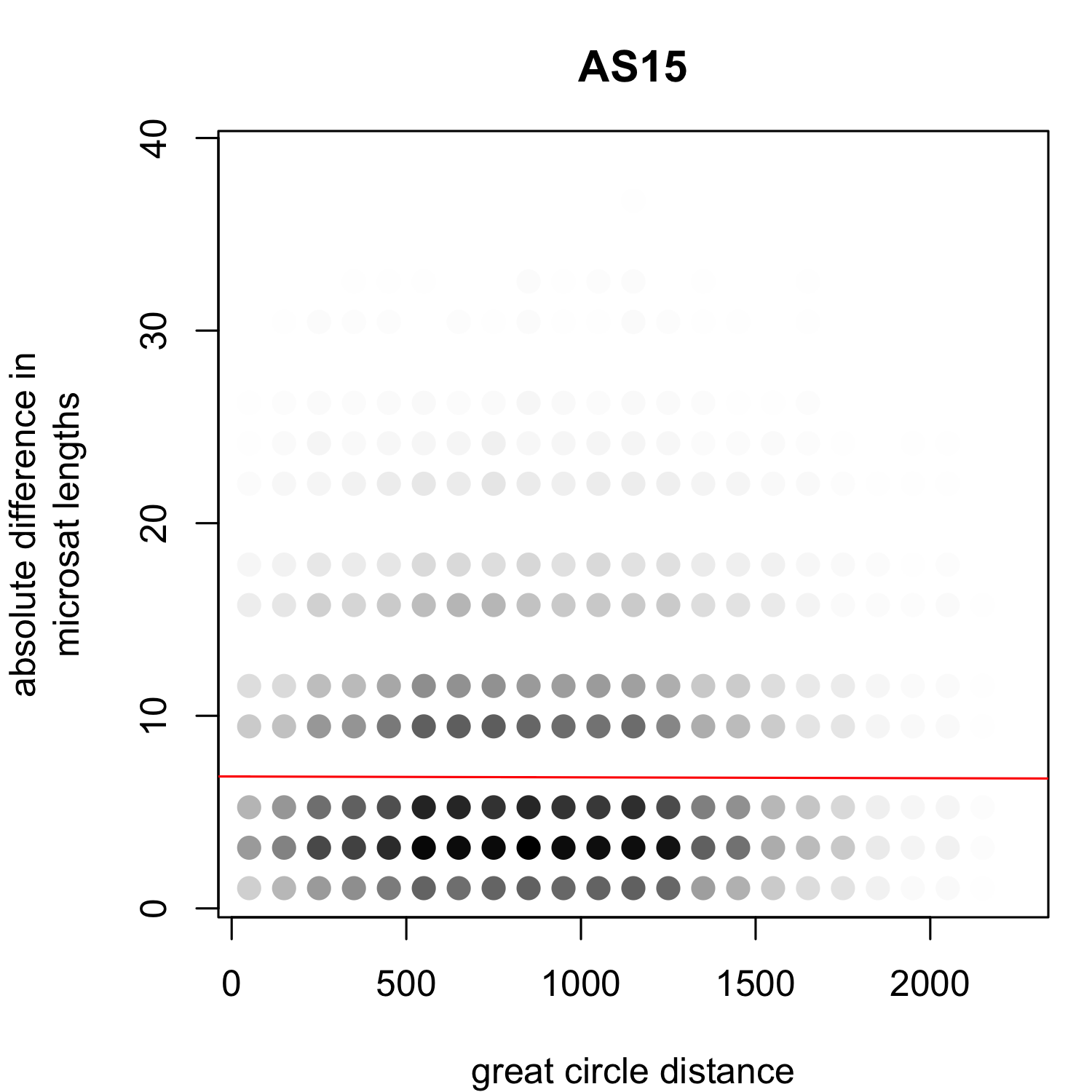*** | ***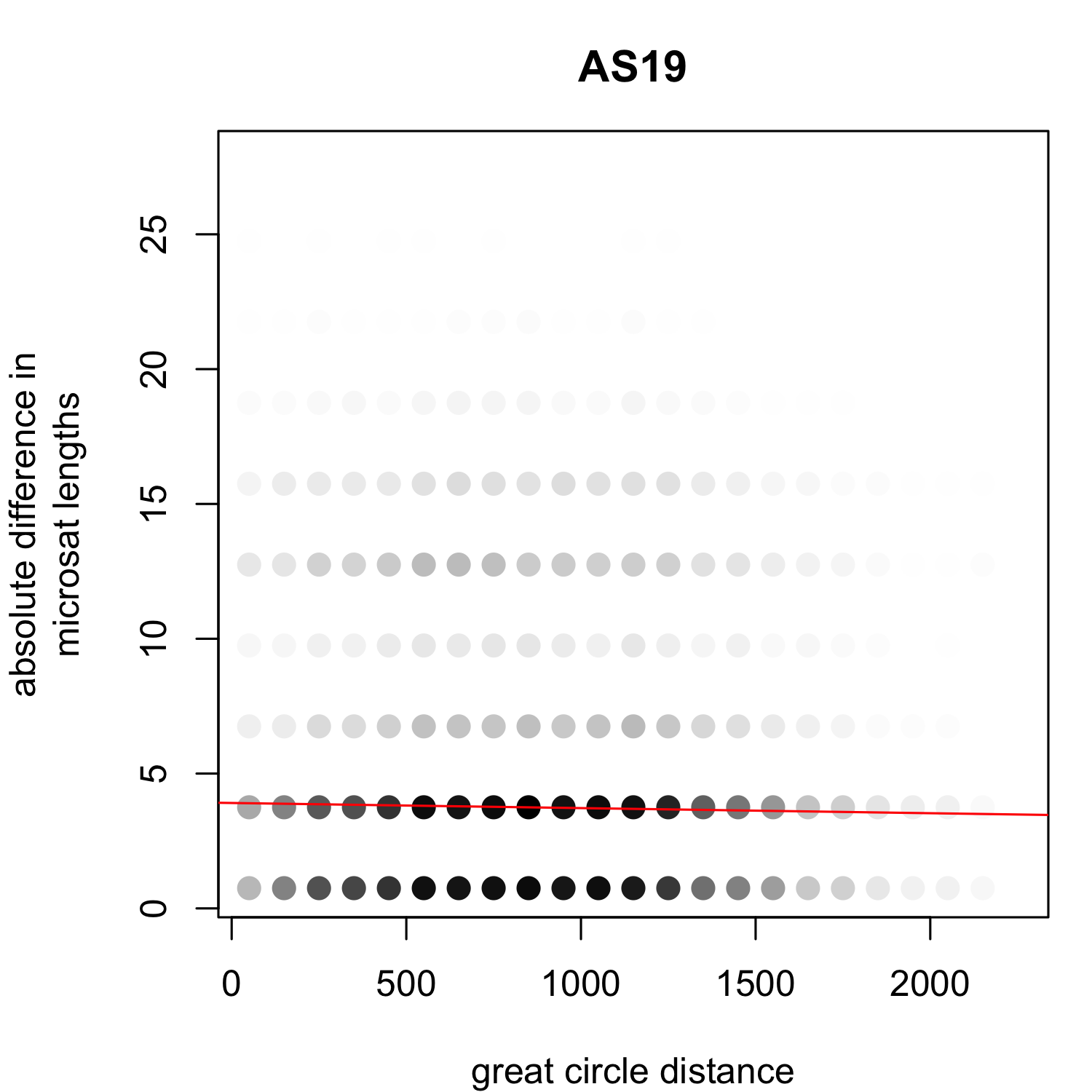*** |
| ***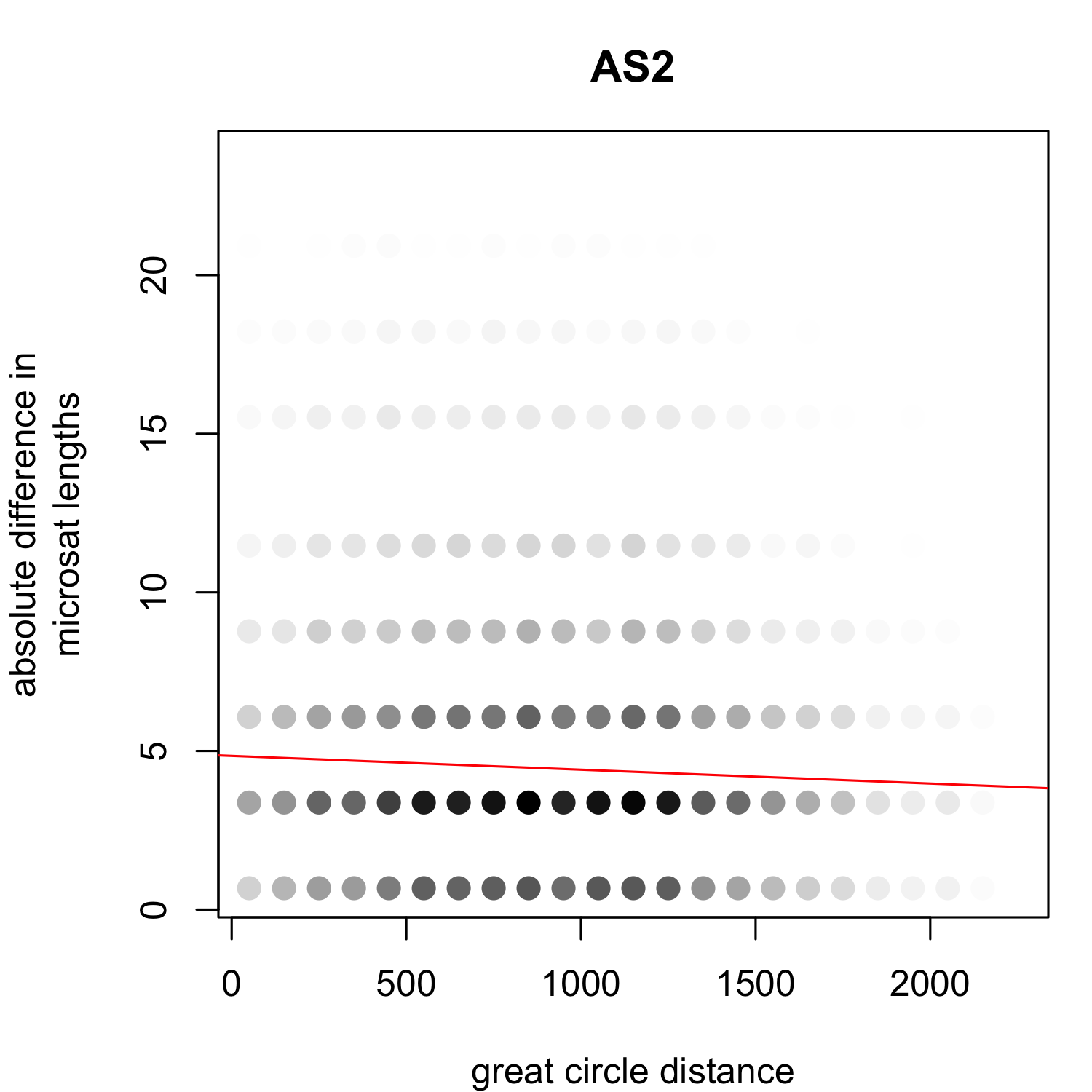*** | ***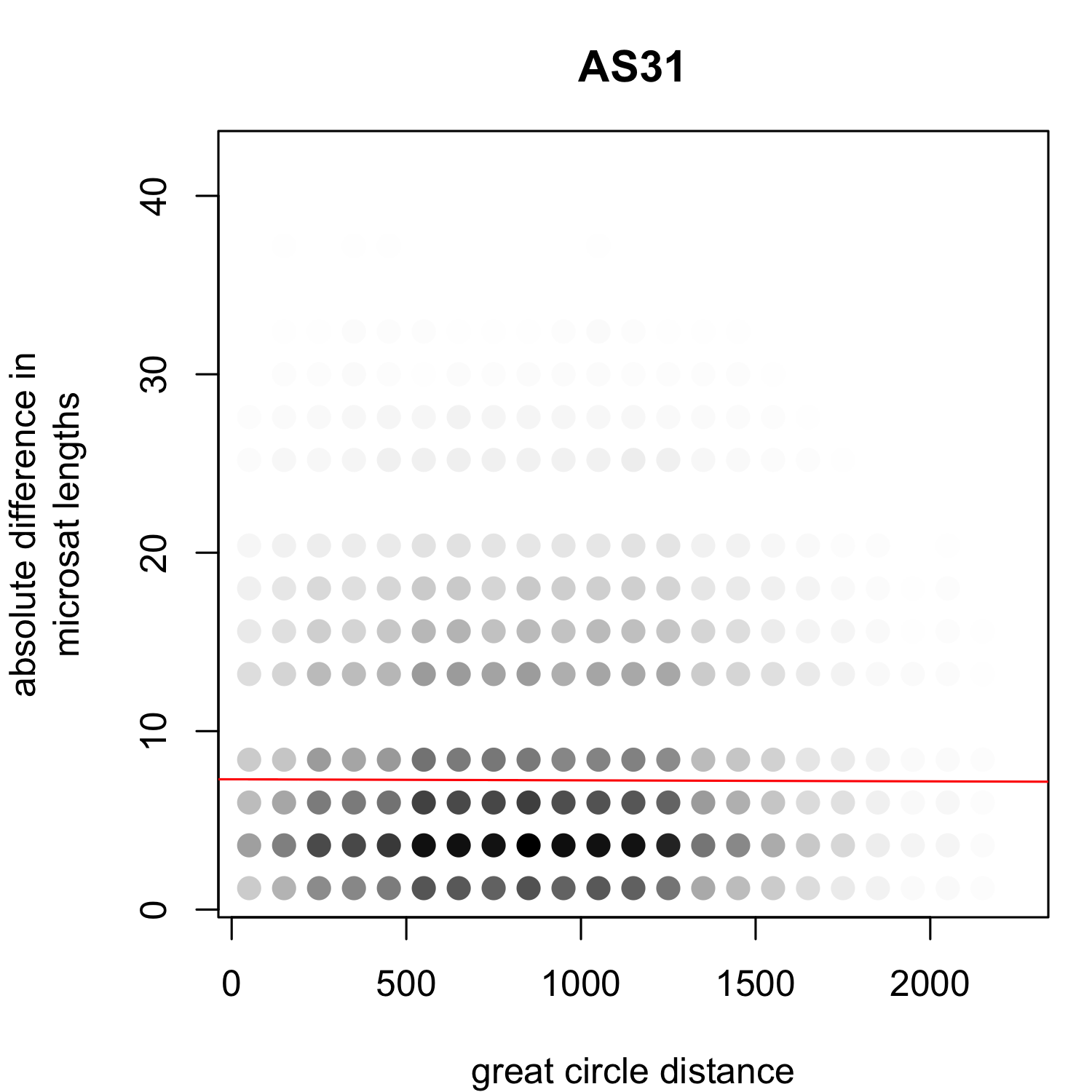*** | ***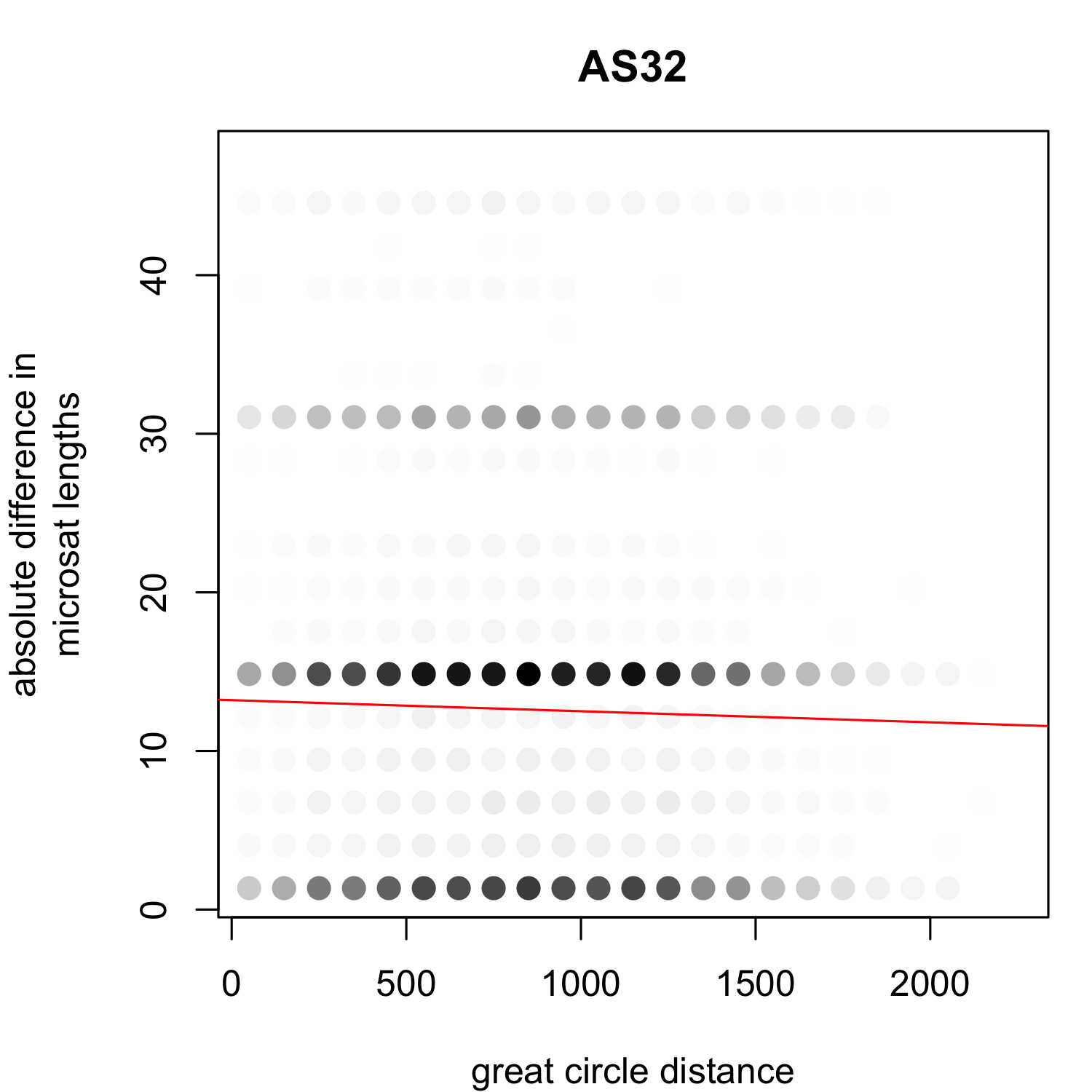*** | ***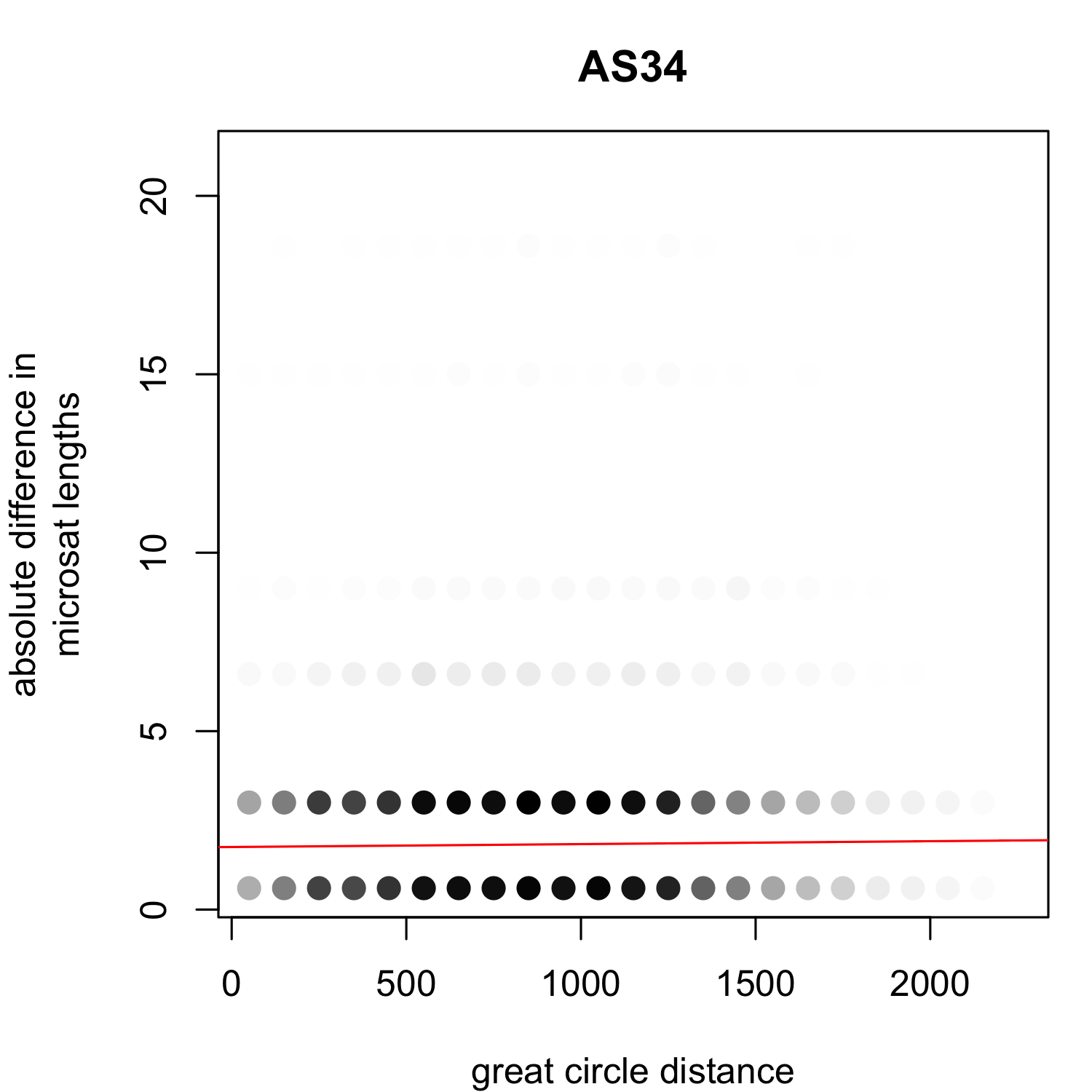*** |
| ***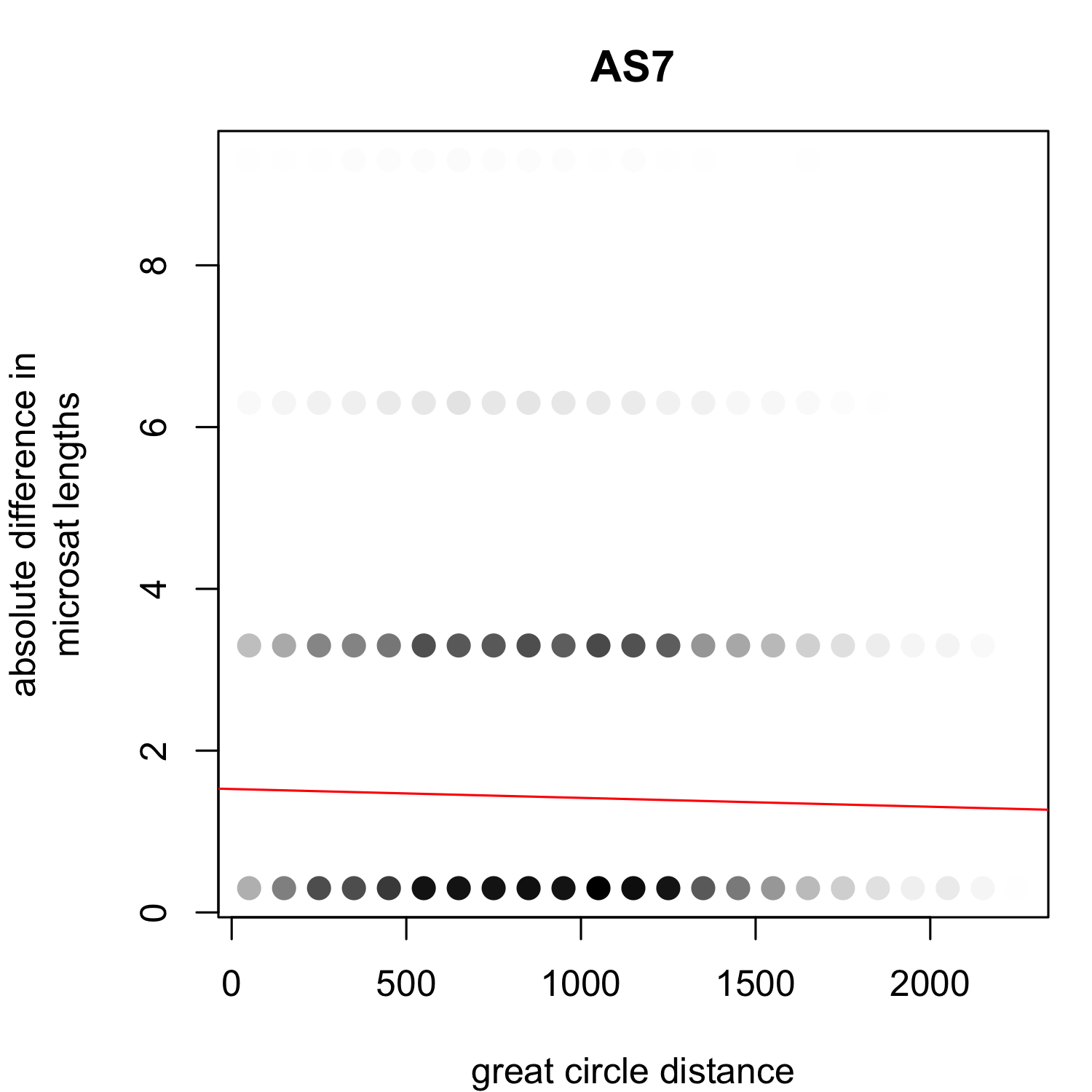*** | ***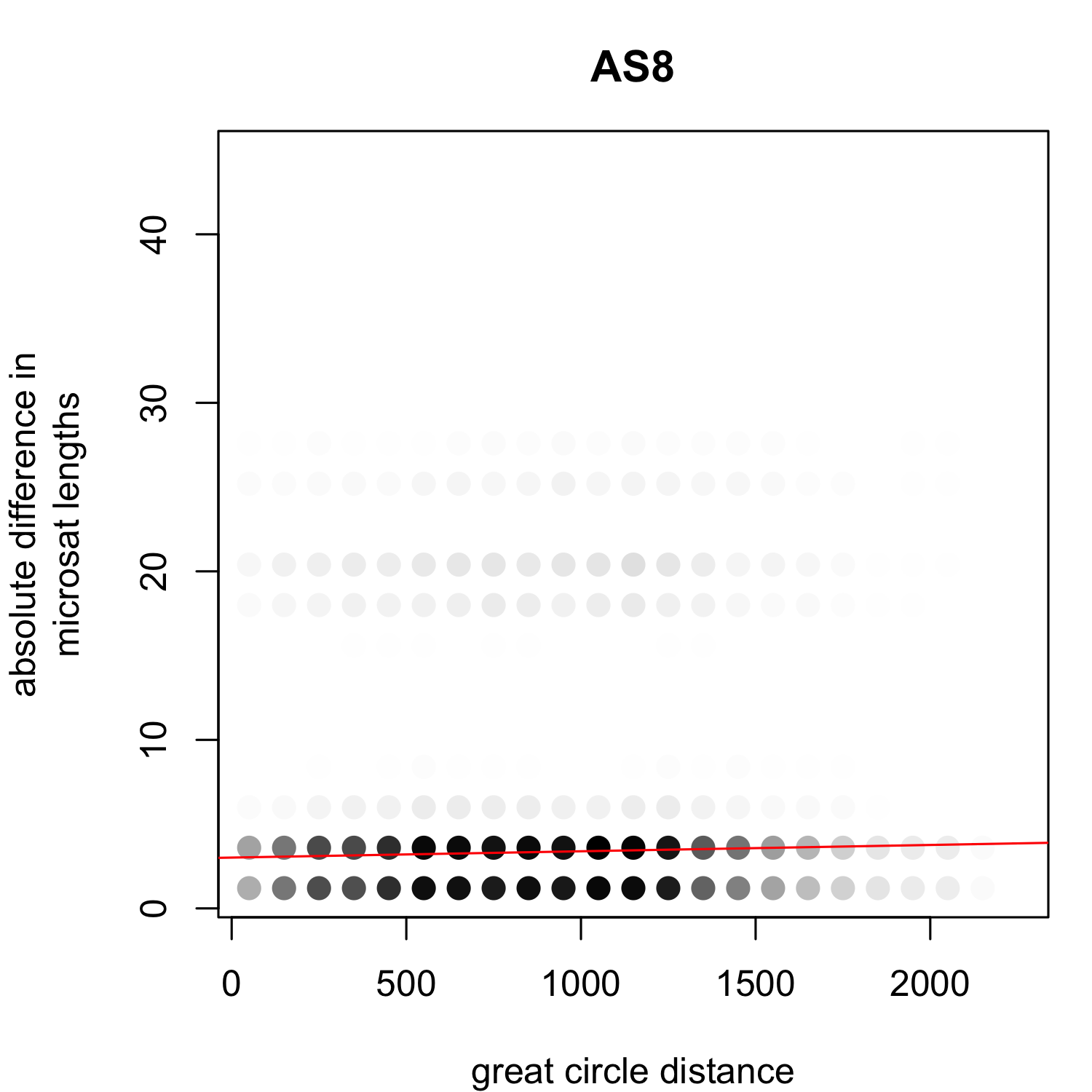*** | ***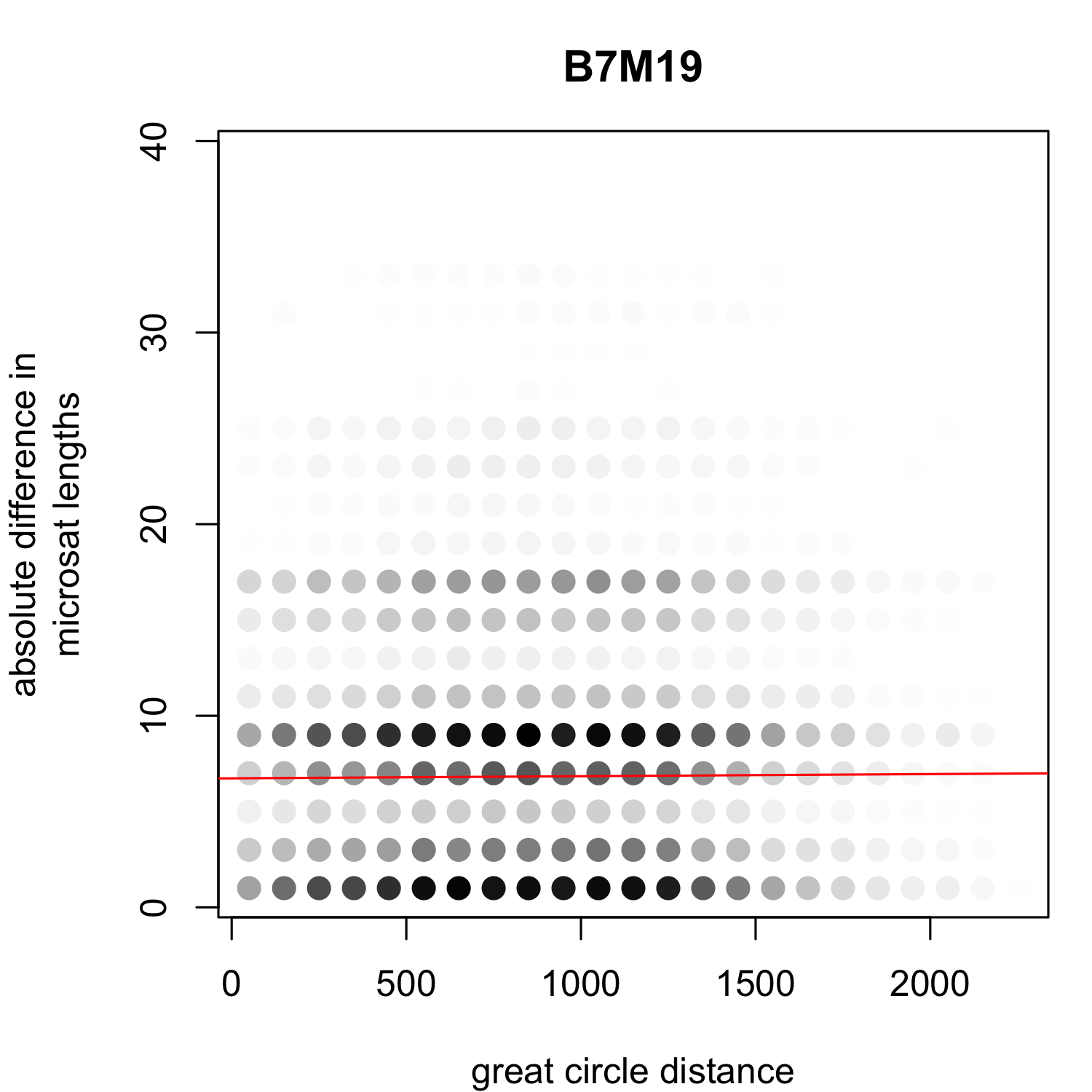*** | ***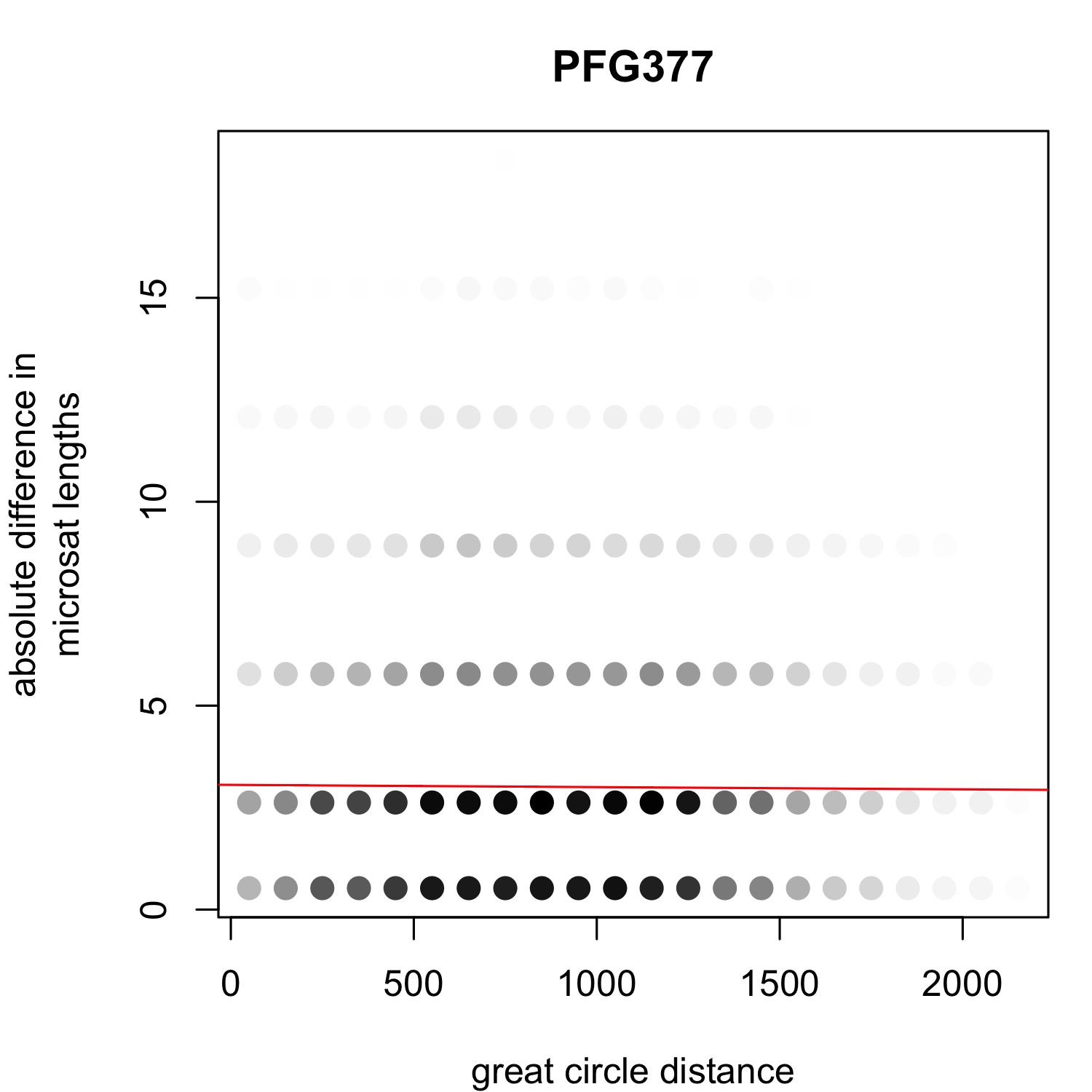*** |
| ***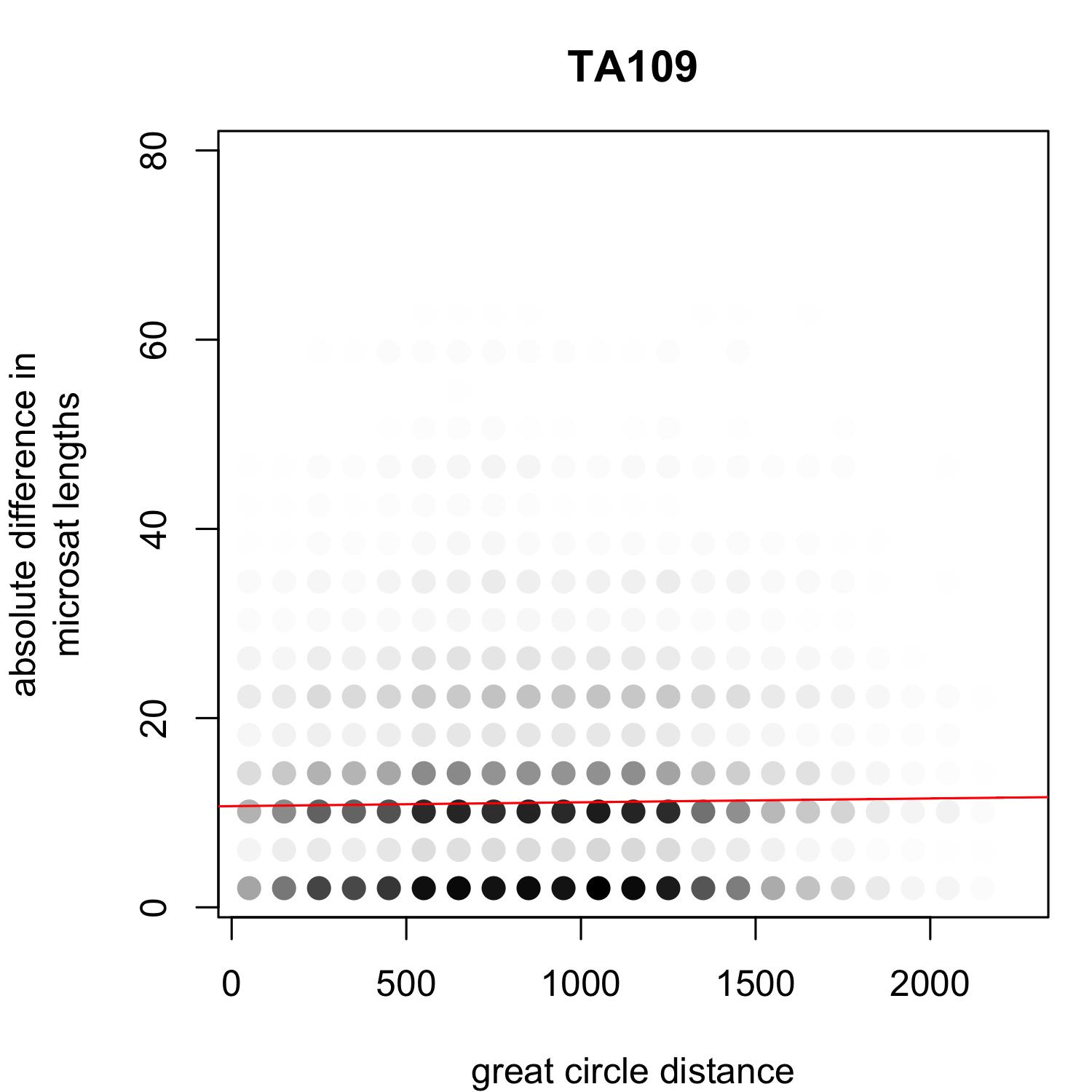*** | ***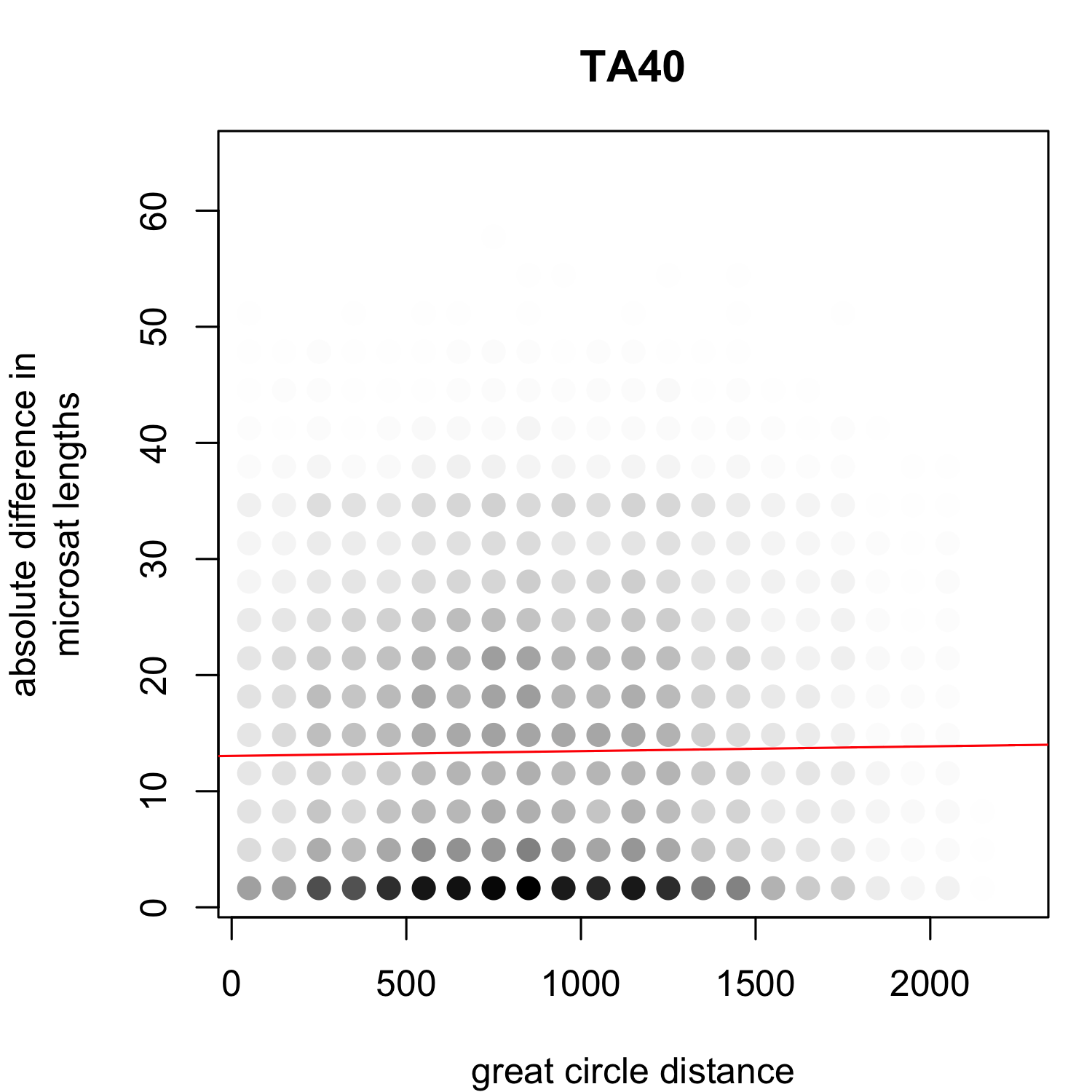*** | ***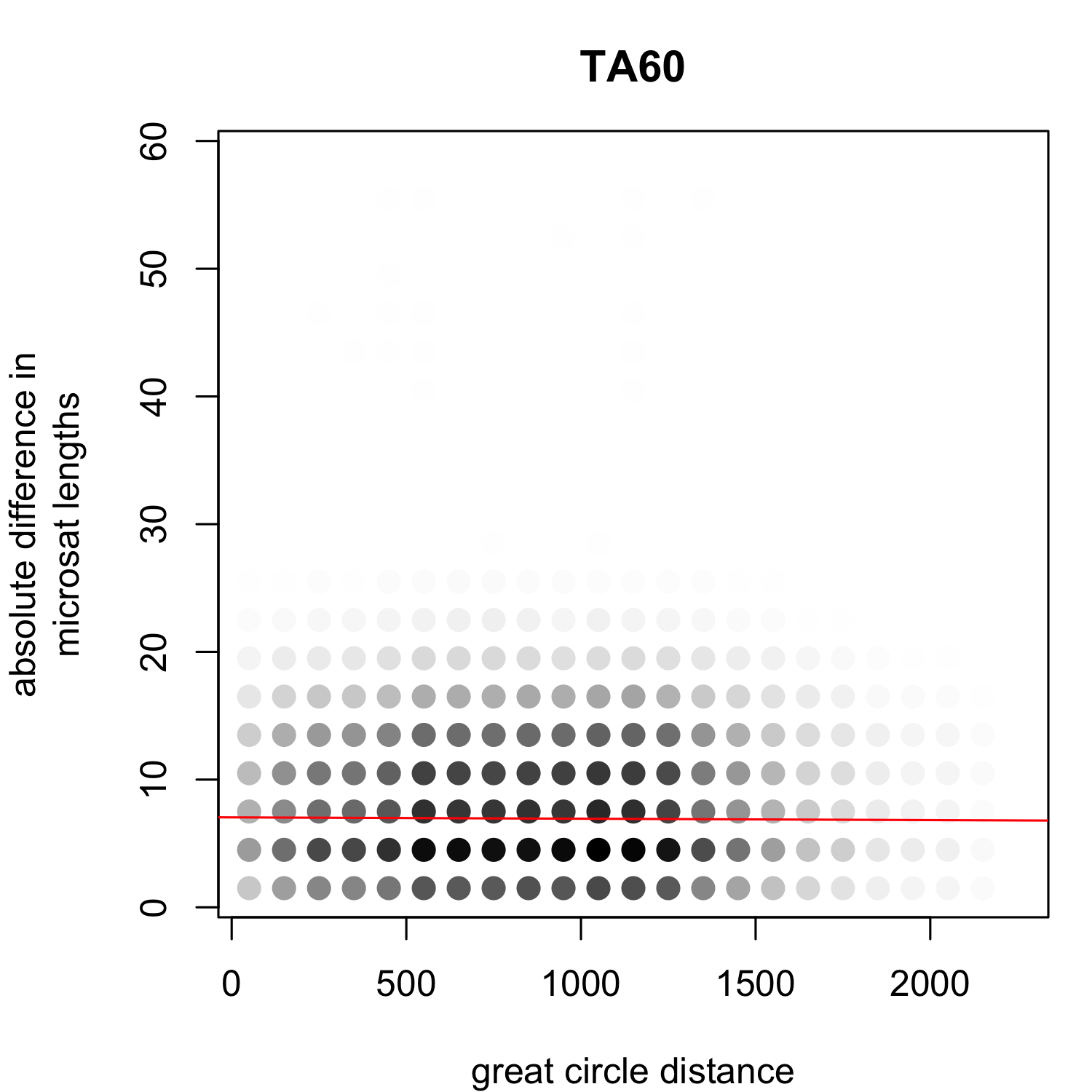*** | ***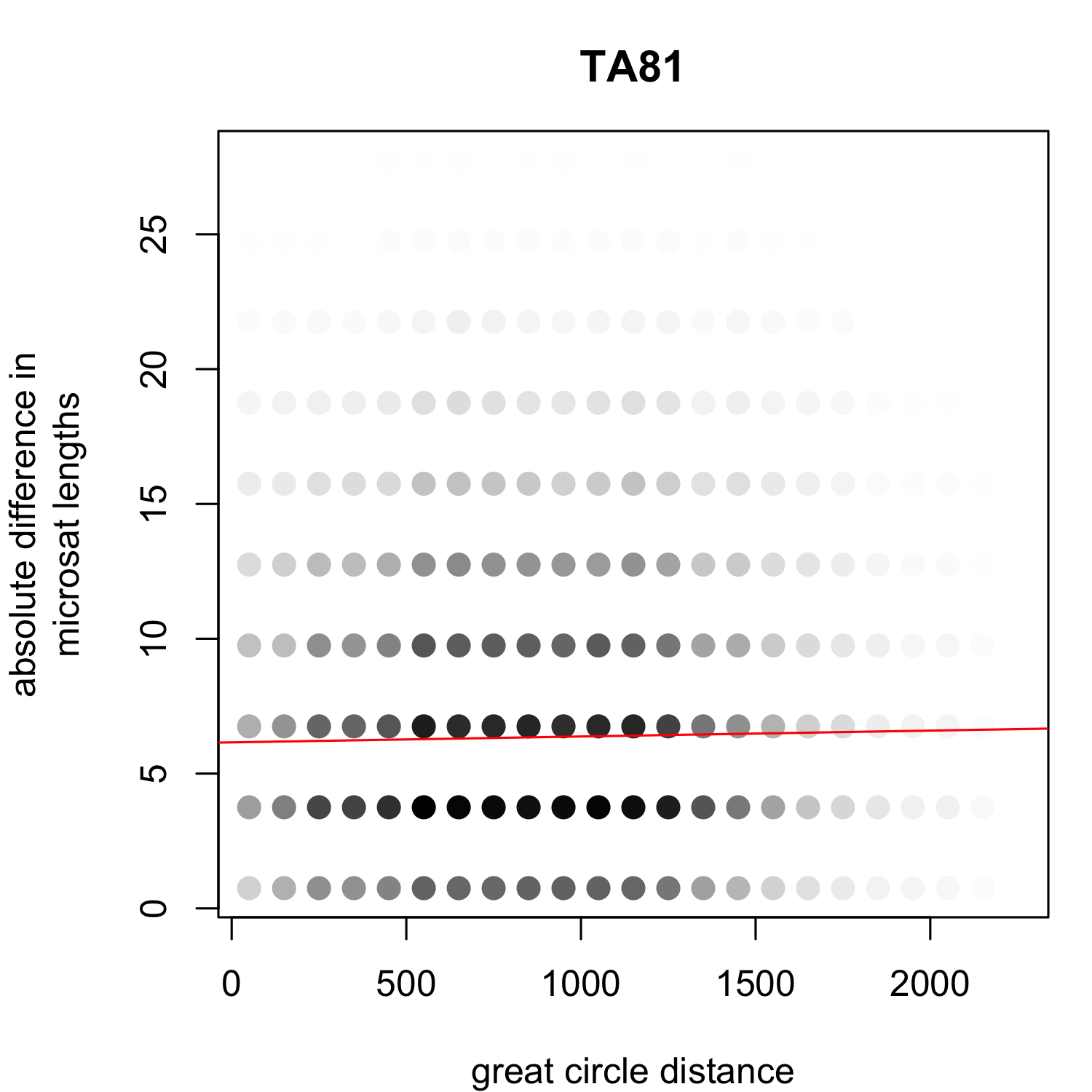*** |

###

### ***Supplementary Figure 9. Test for isolation by distance at each microsatellite locus***

Regression of genetic distance (absolute difference in microsatellite lengths) against geographic distance (great circle distance between clusters). Multiple points have the same combination of genetic and geographic distance, and so shading indicates the density of points for a given combination.
