# Supplementary material for: Drug-Resistance and Population Structure of Plasmodium falciparum Across the Democratic Republic of Congo Using High-Throughput Molecular Inversion Probes
Source: J Infect Dis. 2018 Apr 28;218(6):946–55. doi: 10.1093/infdis/jiy223 (PMC6093412; doi:10.1093/infdis/jiy223)
Supplement: Supplementary Figure10 [file jiy223_suppl_supplementary_figure10.docx]

###
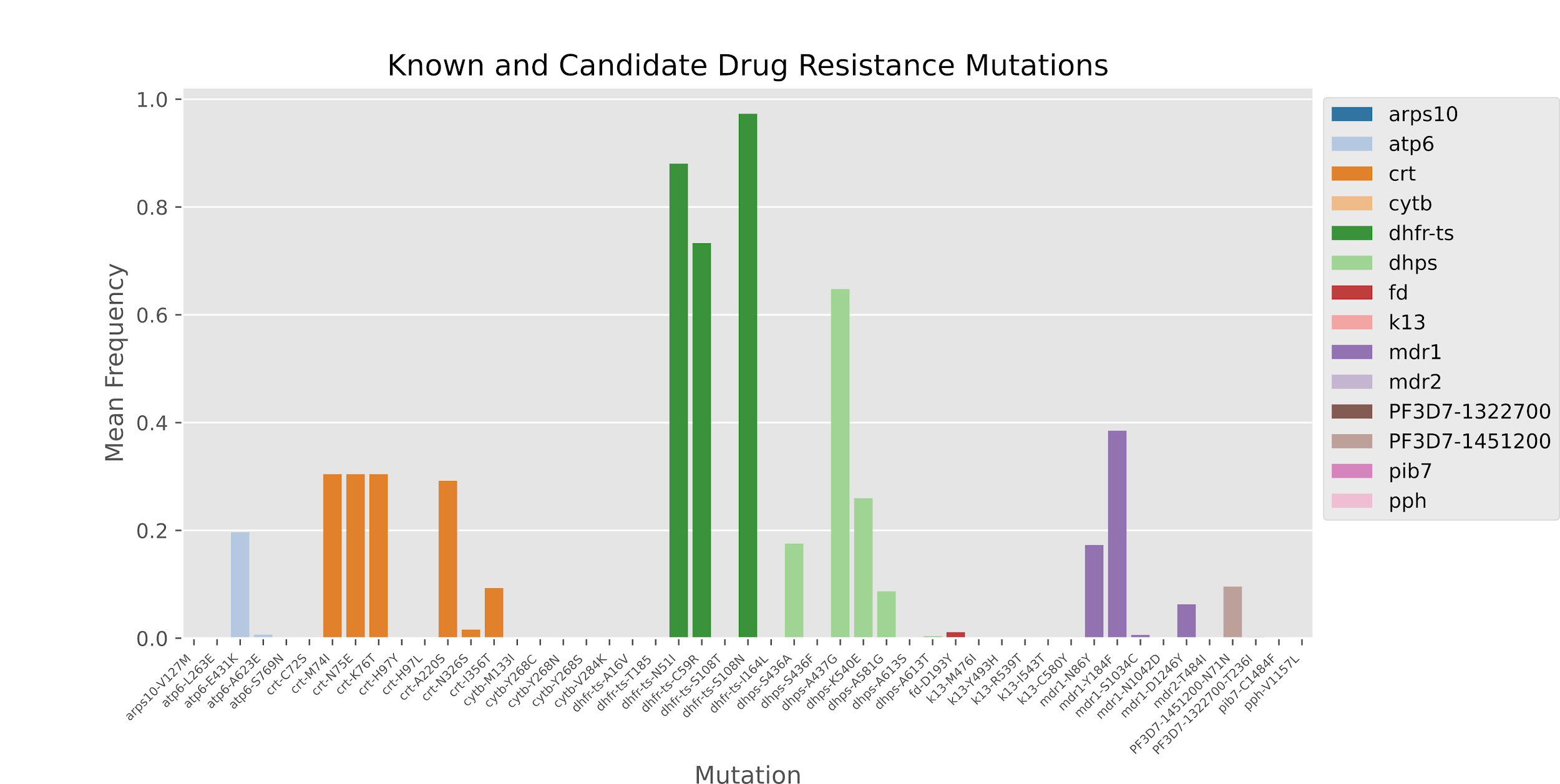


### ***Supplementary Figure 10. Drug resistance mutation frequencies***

The drug resistance mutation frequencies were calculated combining the relative allele frequencies in each individual sample with equal weighting. These values were comparable to the prevalences.
