# Supplementary material for: Drug-Resistance and Population Structure of Plasmodium falciparum Across the Democratic Republic of Congo Using High-Throughput Molecular Inversion Probes
Source: J Infect Dis. 2018 Apr 28;218(6):946–55. doi: 10.1093/infdis/jiy223 (PMC6093412; doi:10.1093/infdis/jiy223)
Supplement: Supplementary Figure11 [file jiy223_suppl_supplementary_figure11.docx]

| **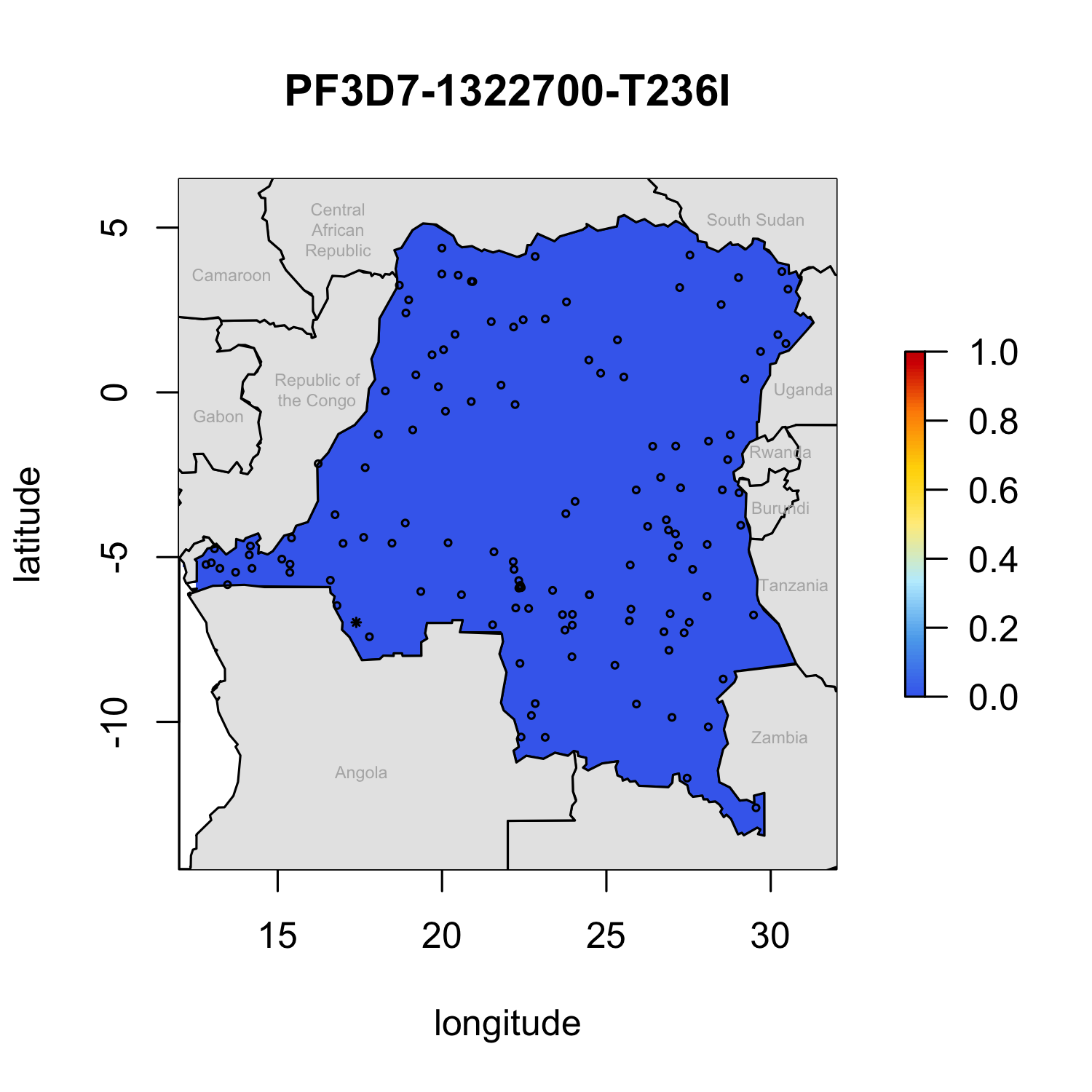** | **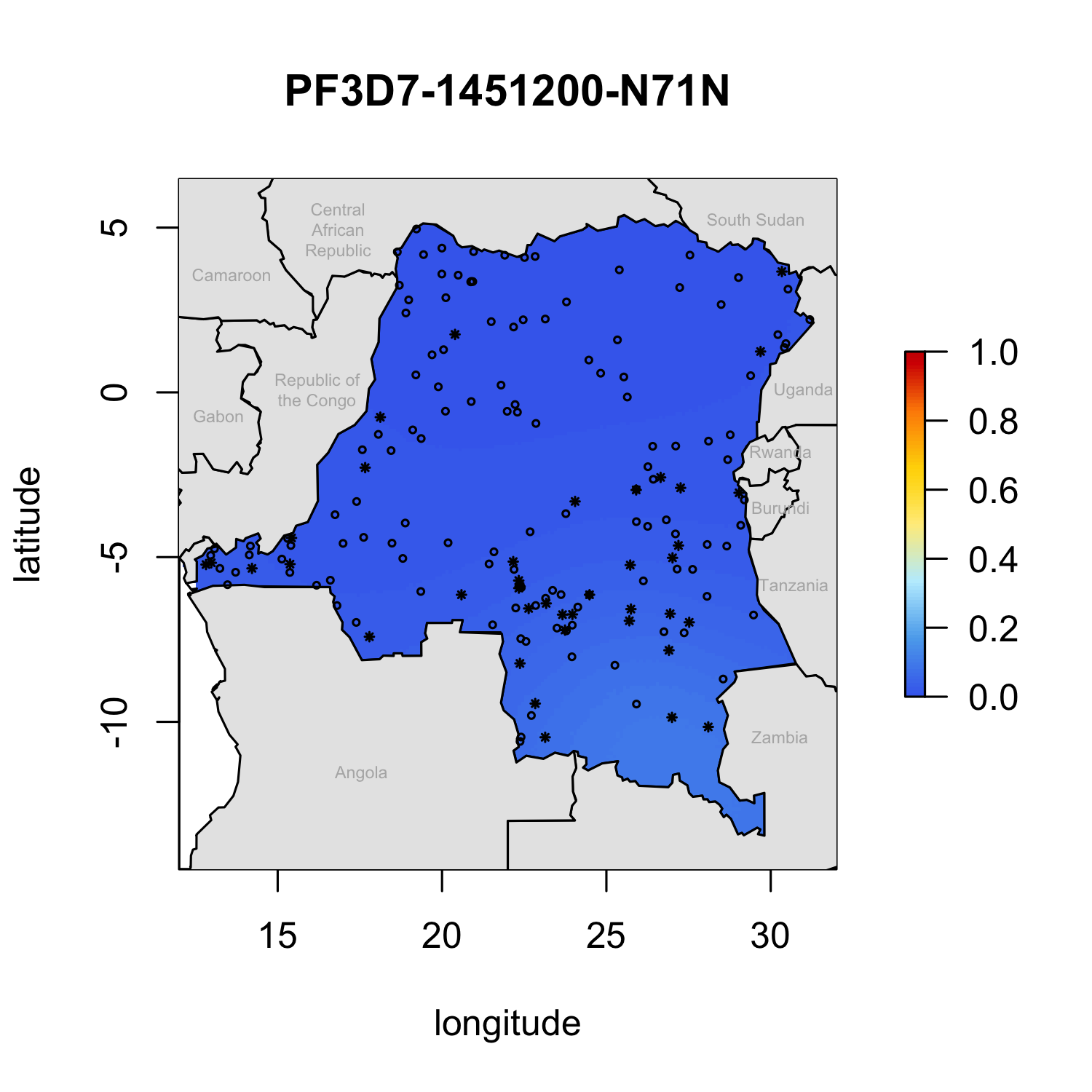** | **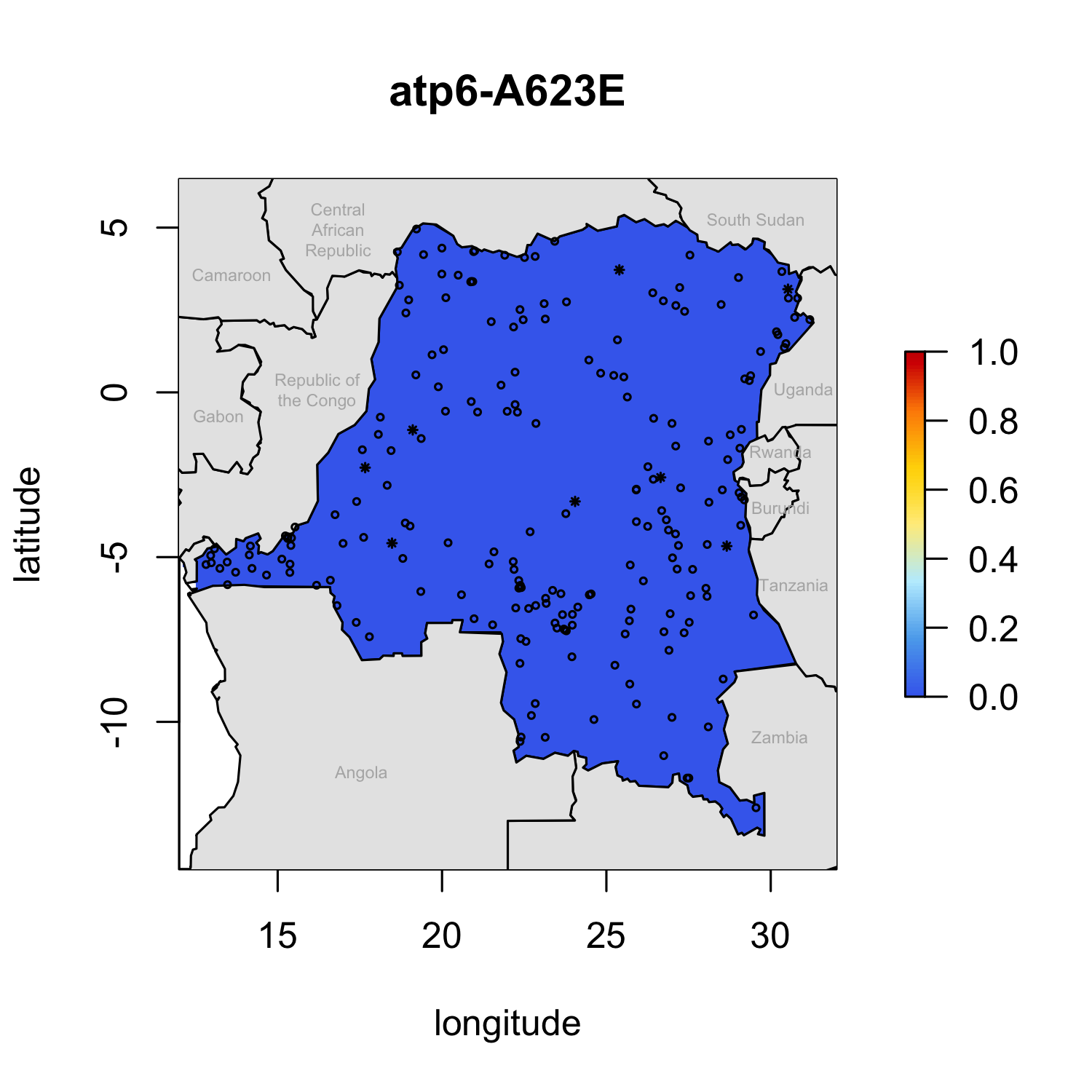** | **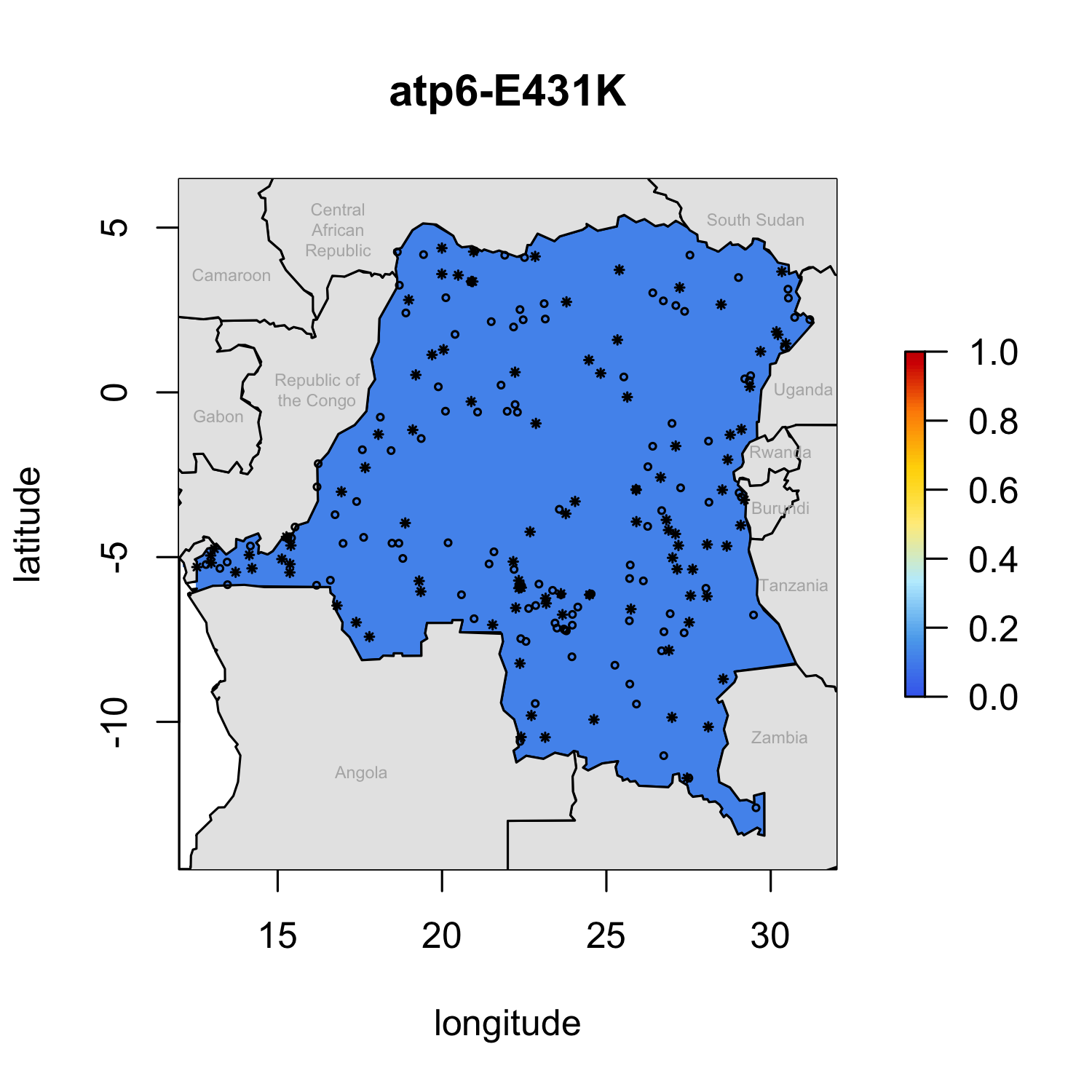** |
| --- | --- | --- | --- |
| **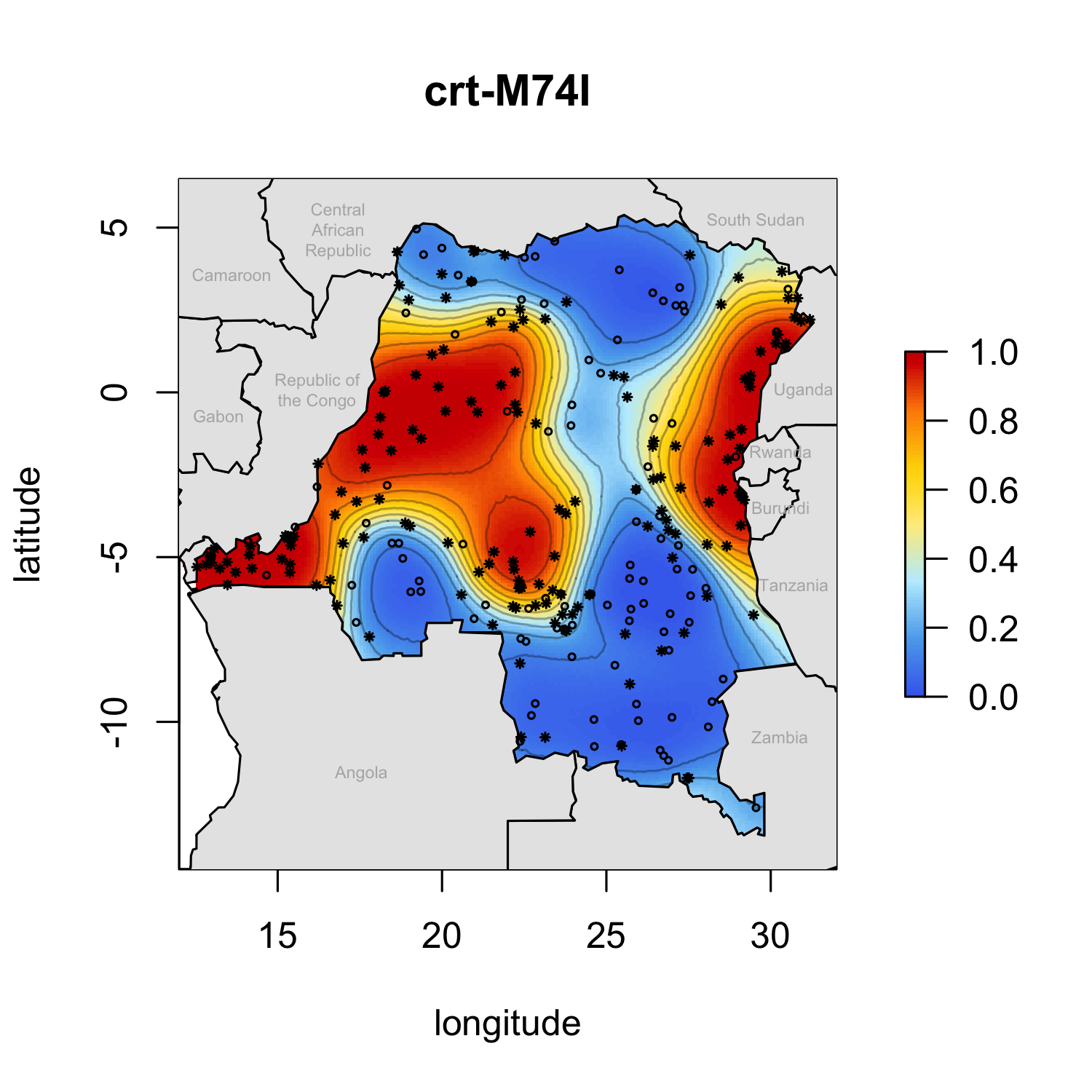** | **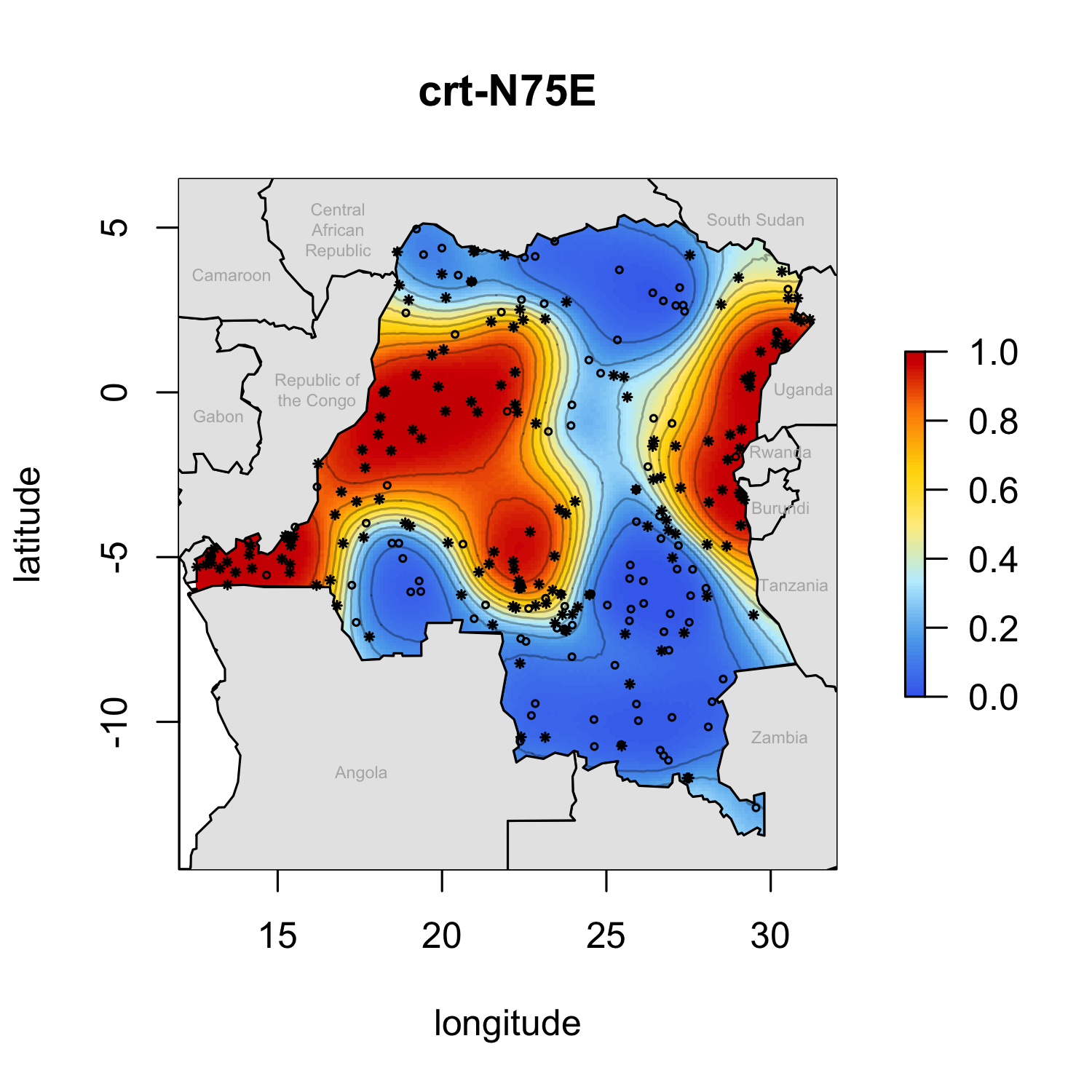** | **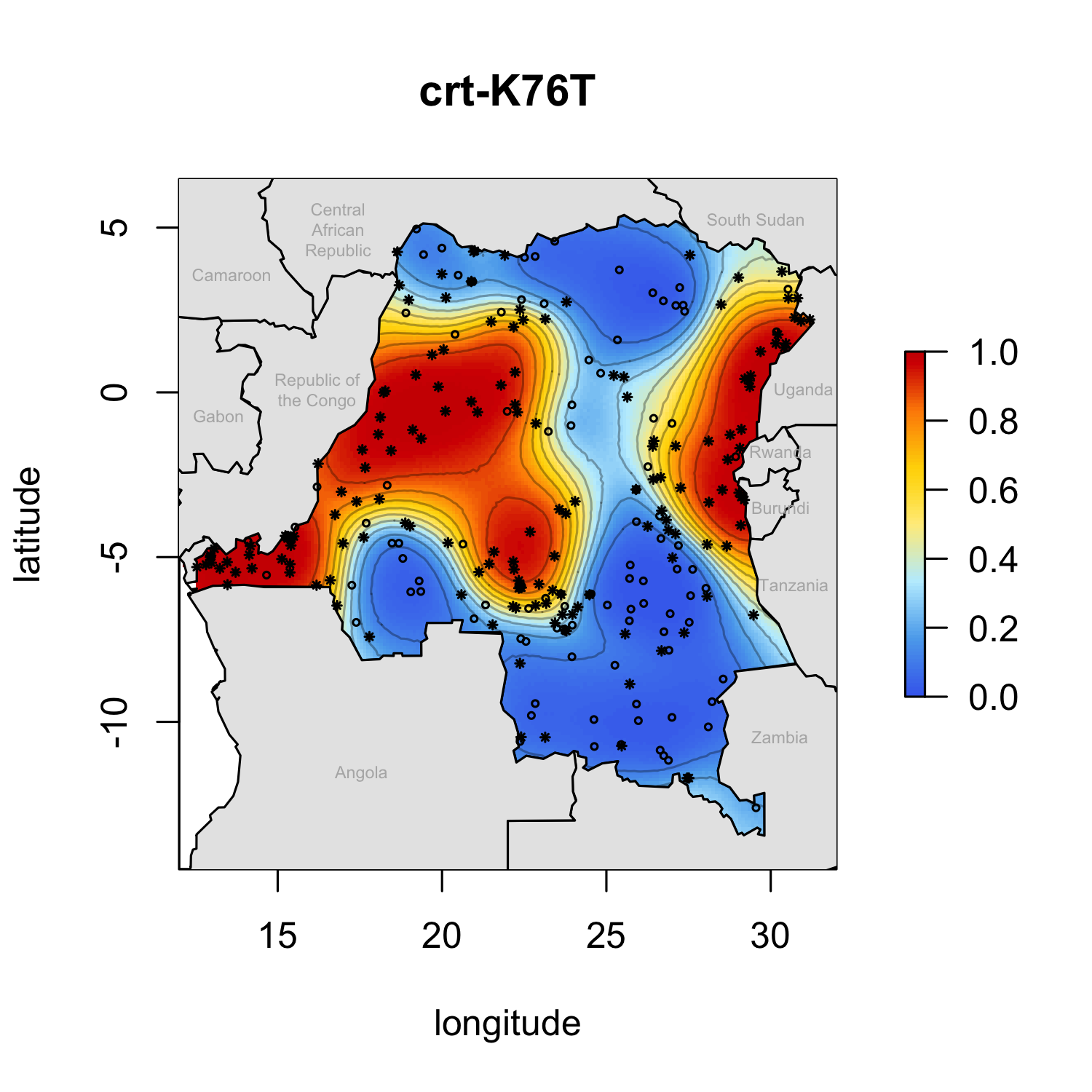** | **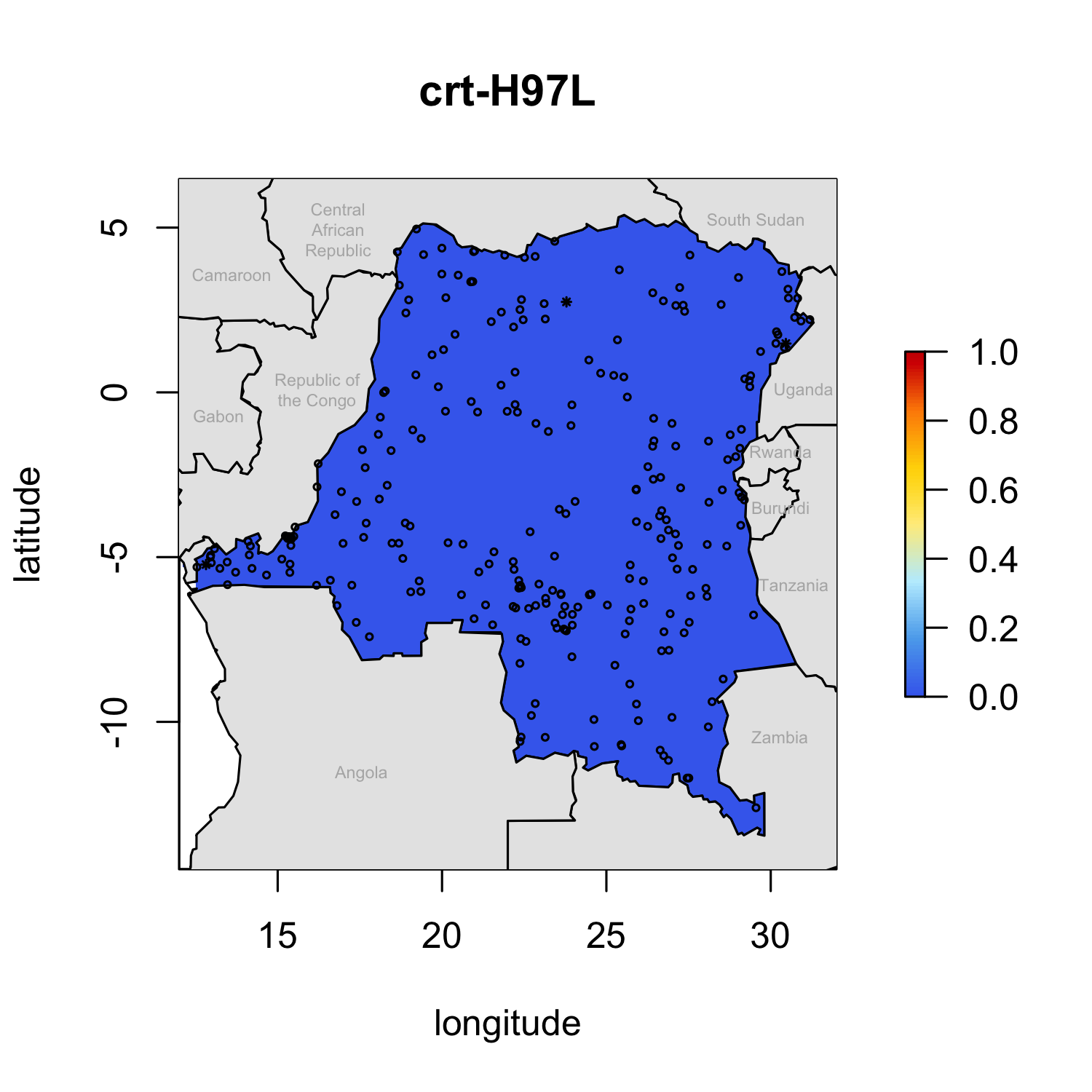** |
| **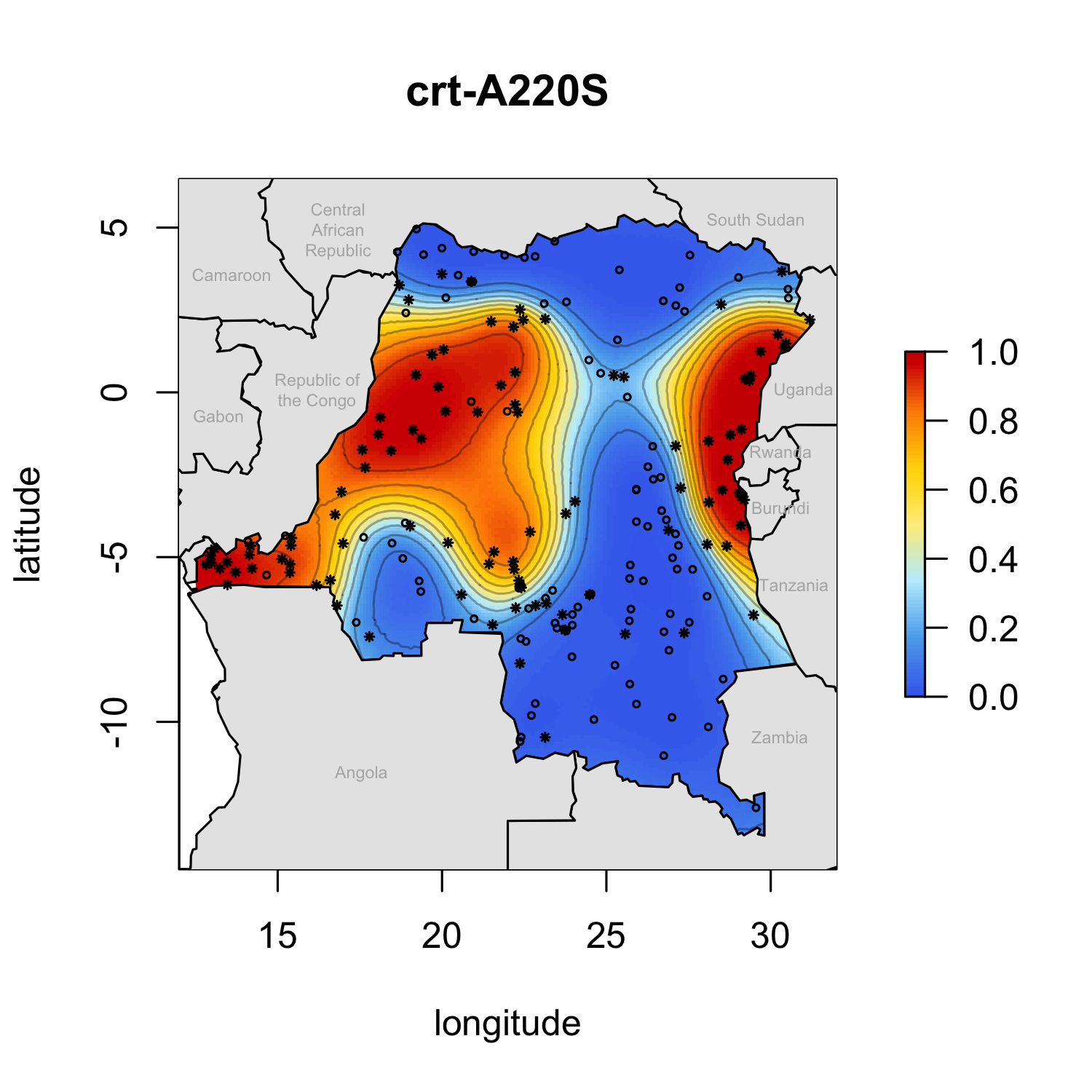** | **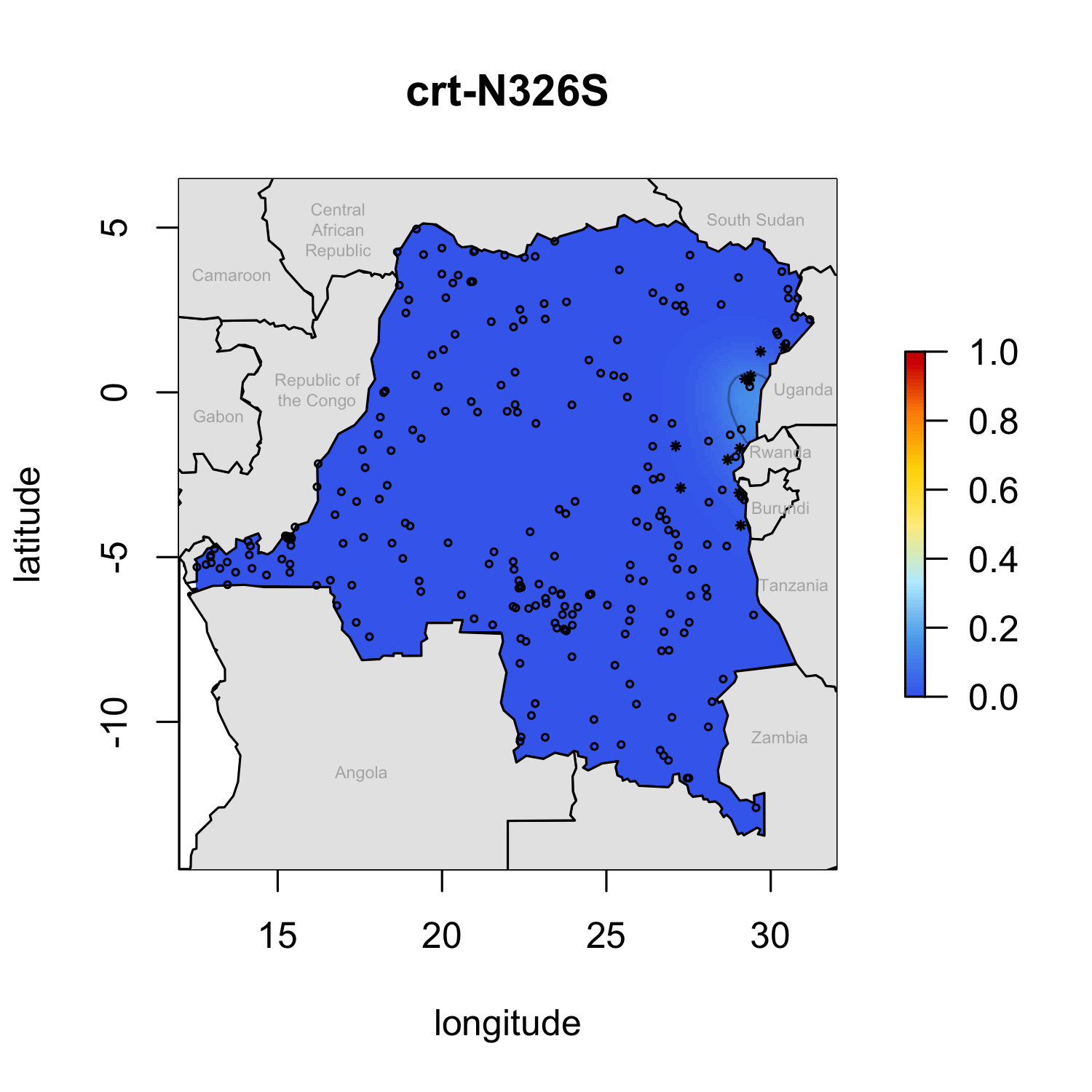** | **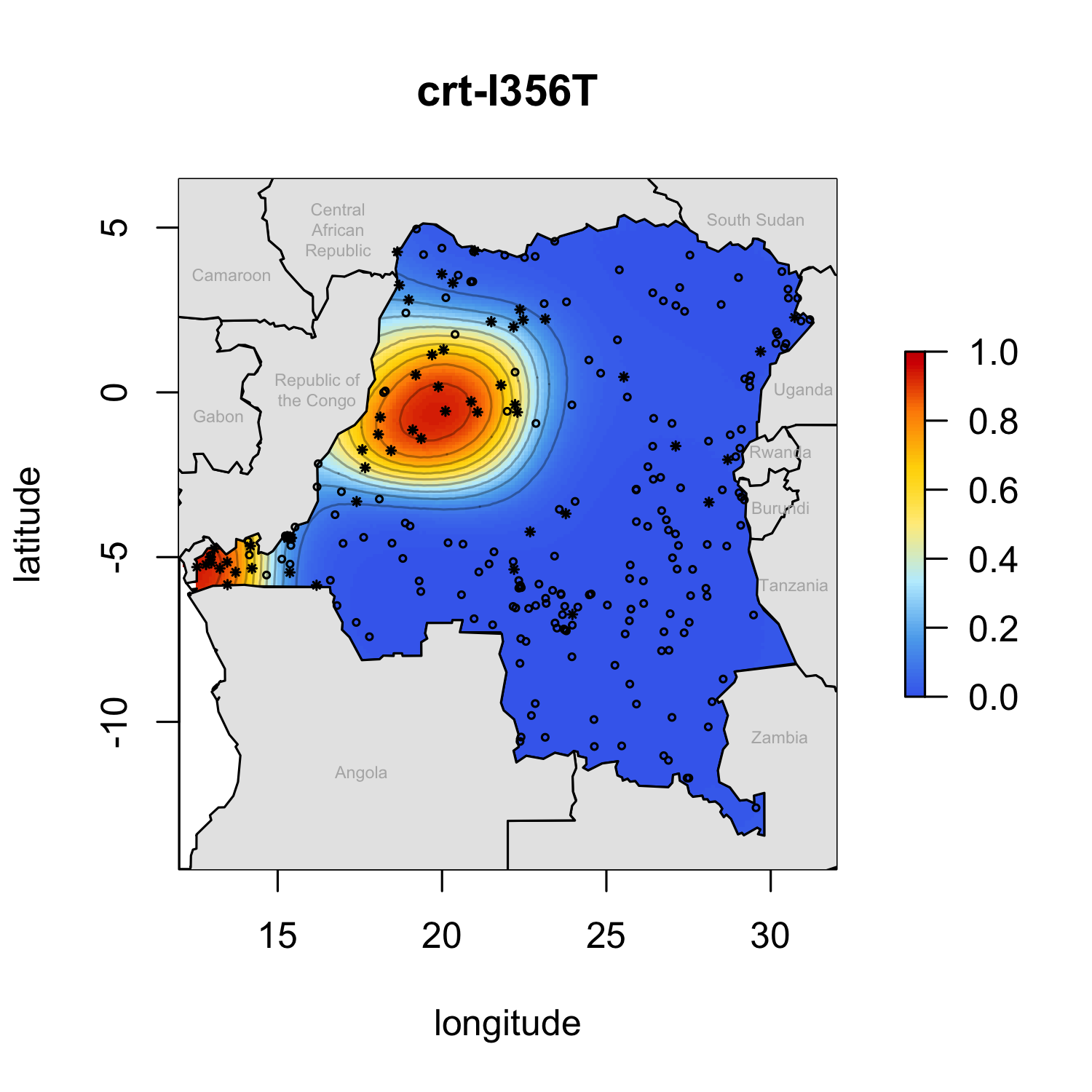** | **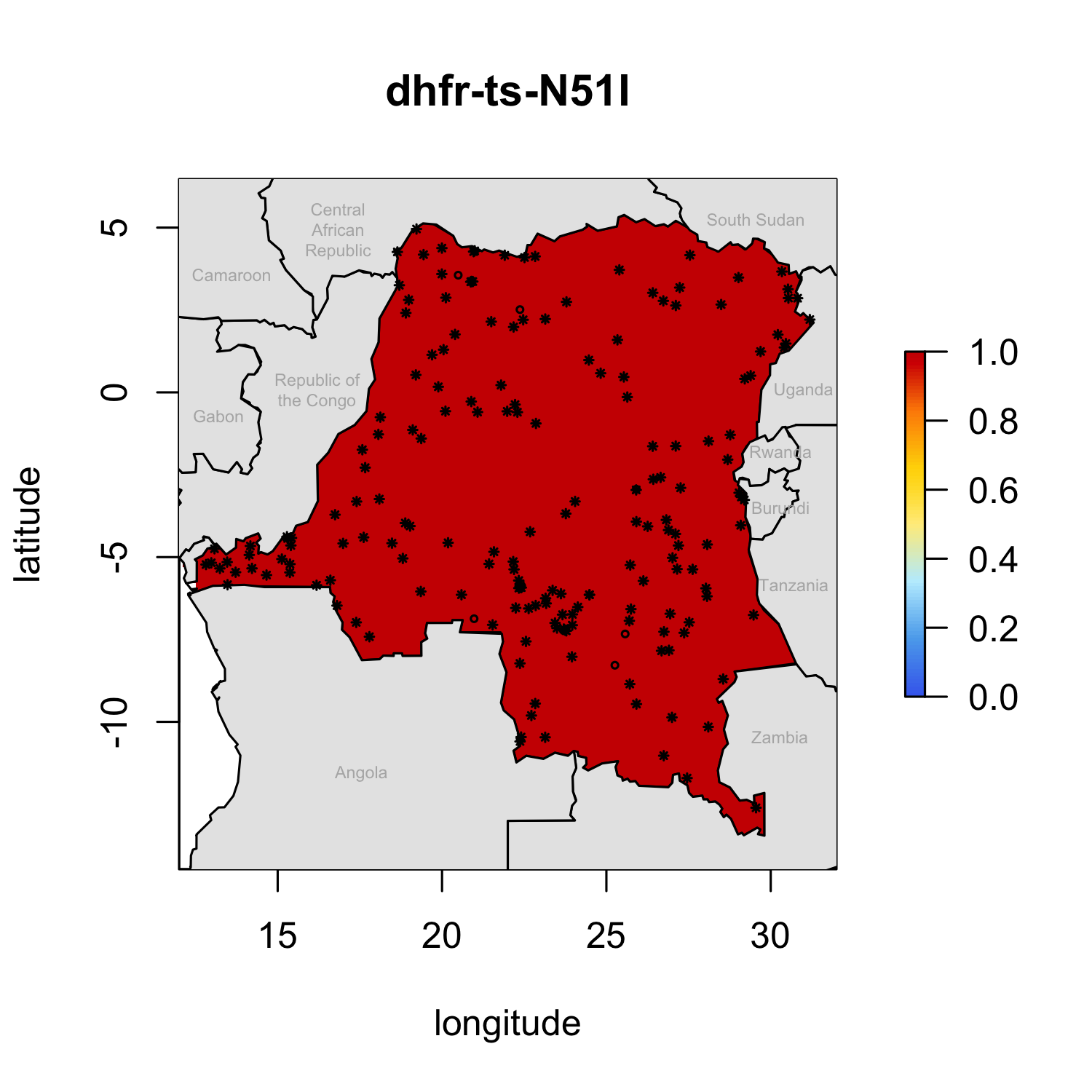** |
| **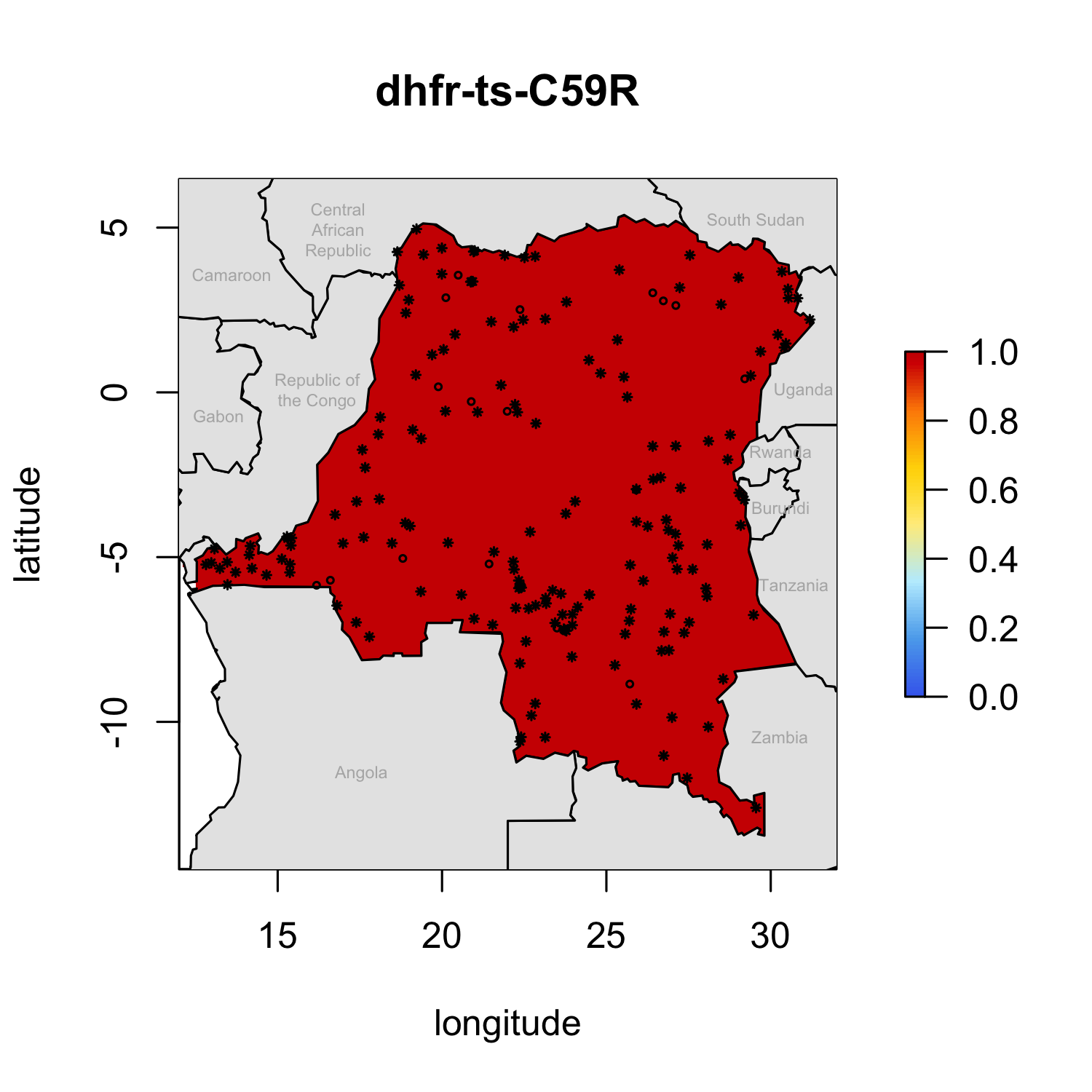** | **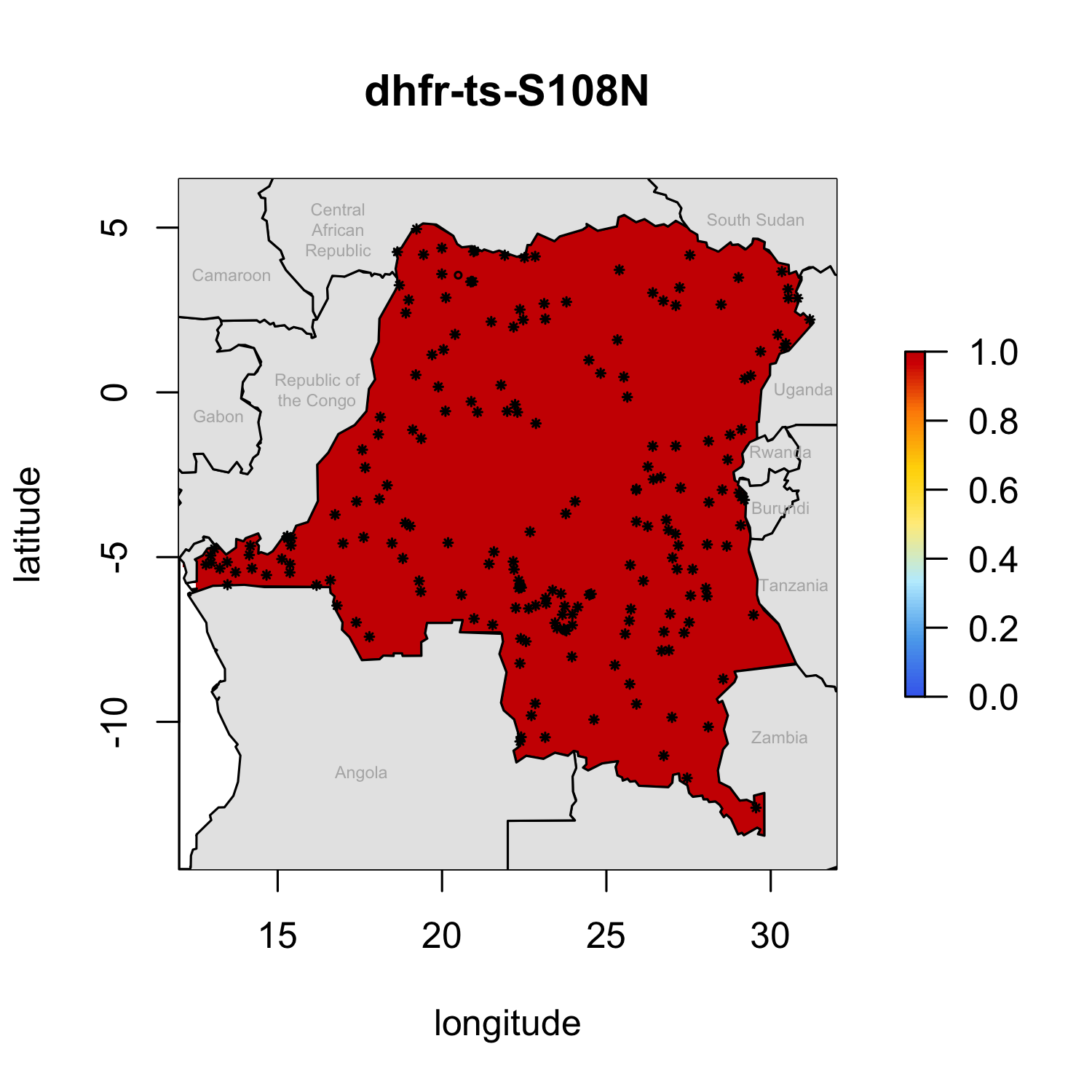** | **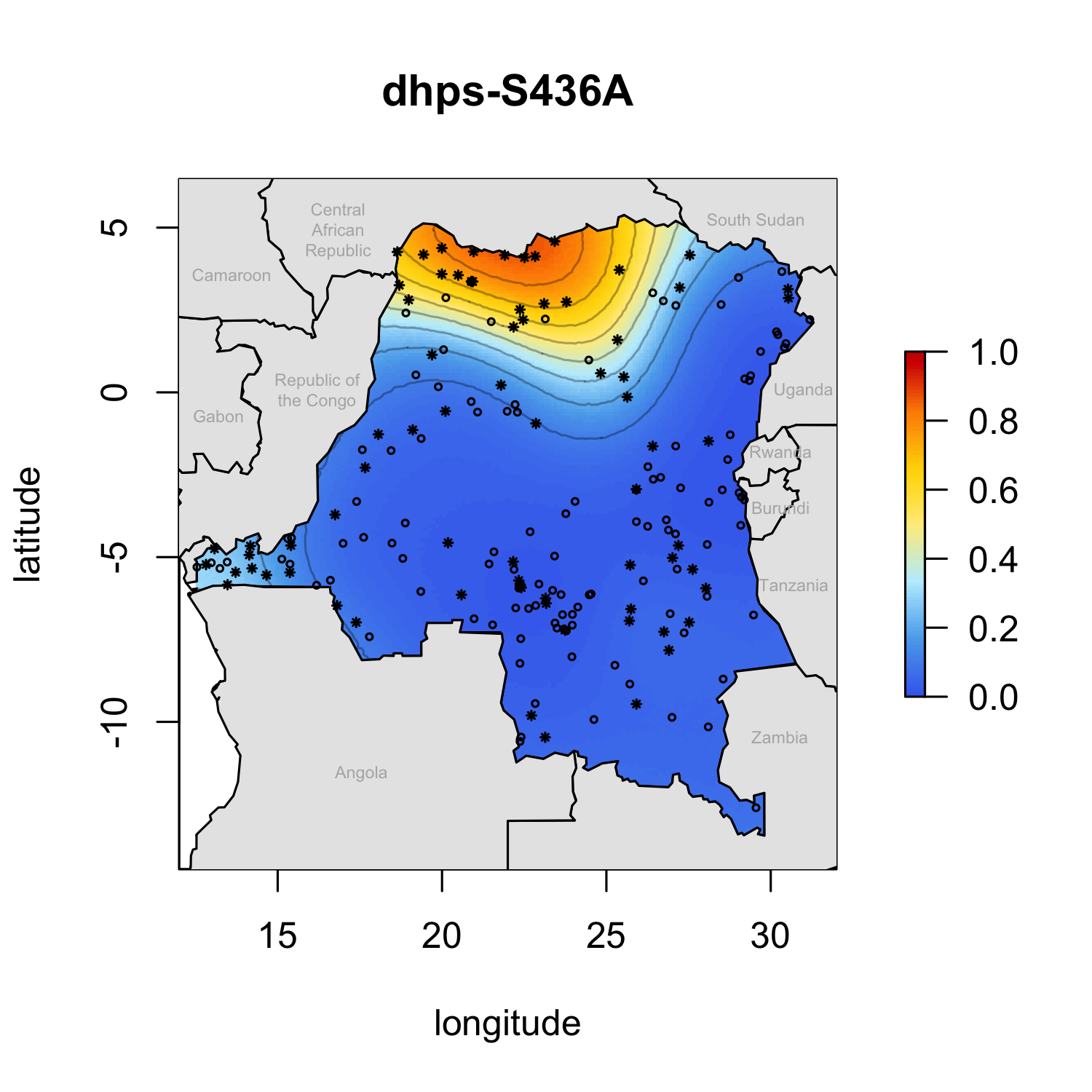** | **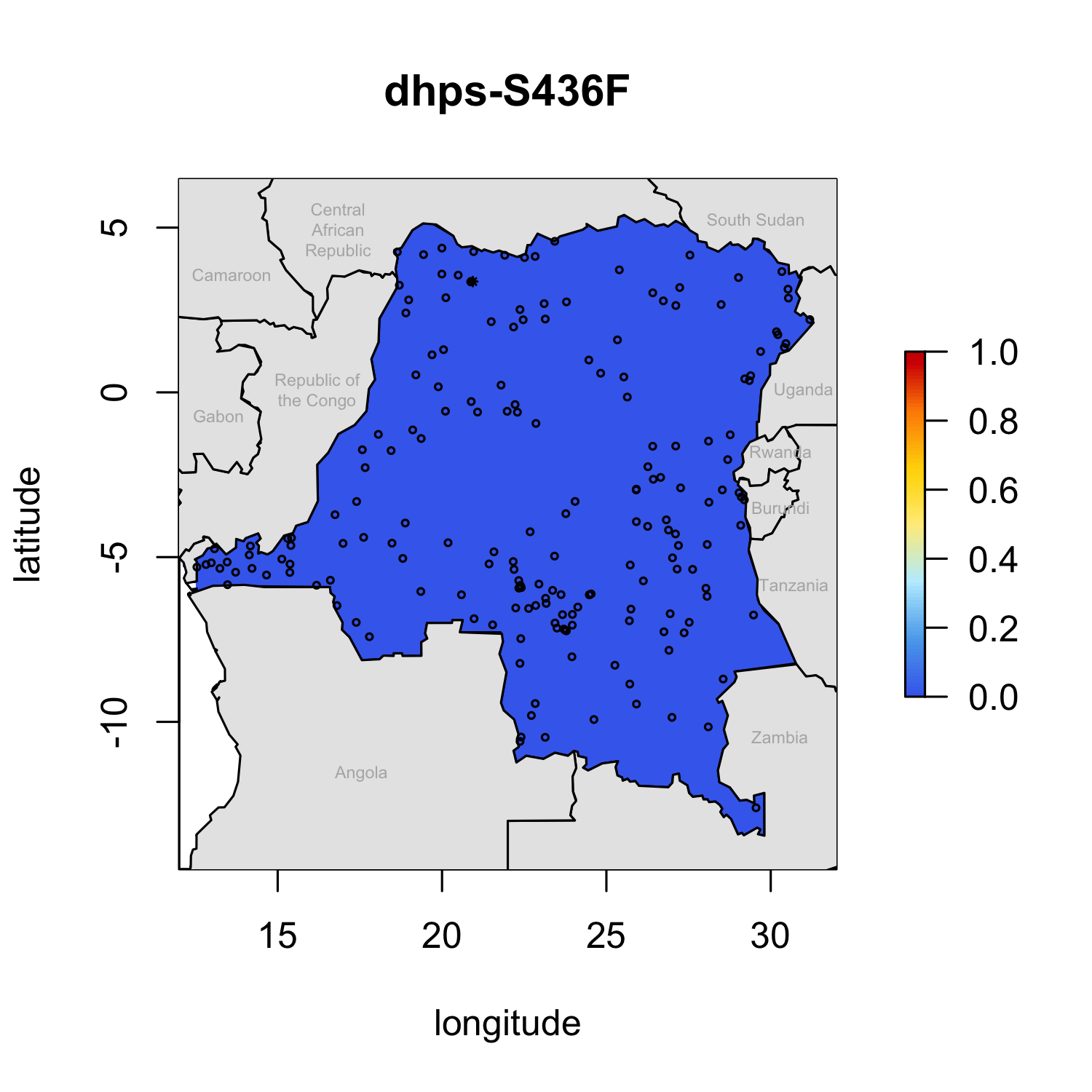** |

| **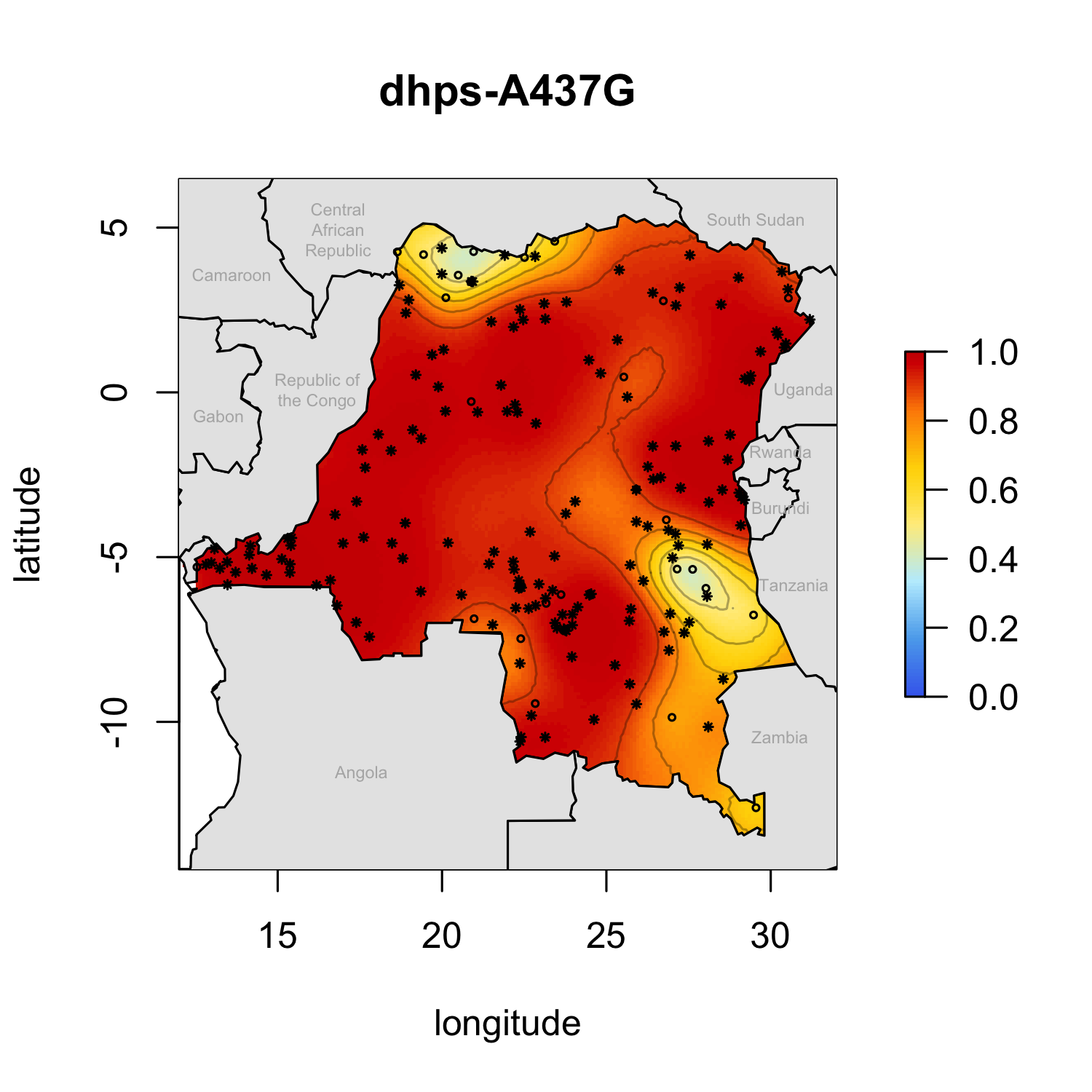** | **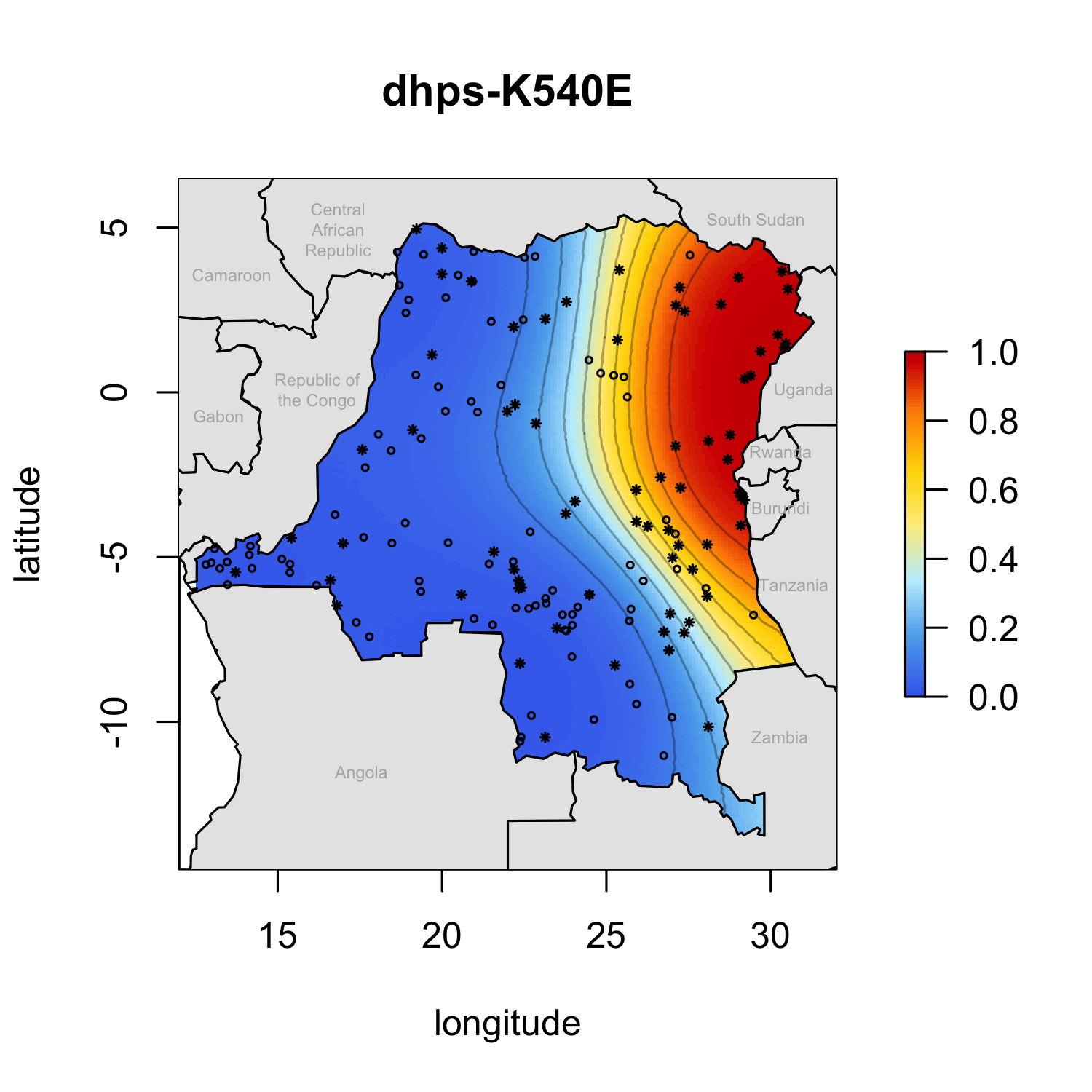** | **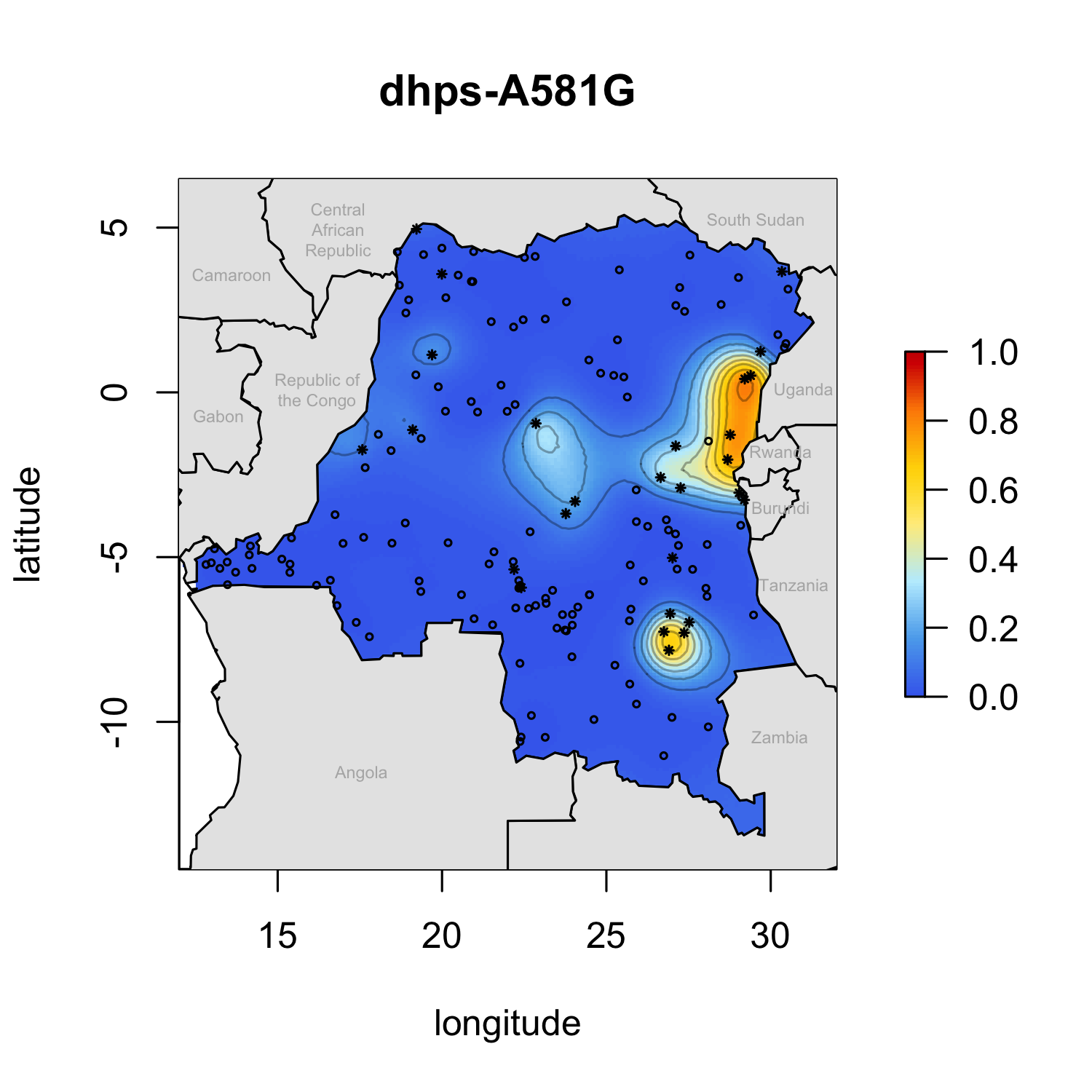** | **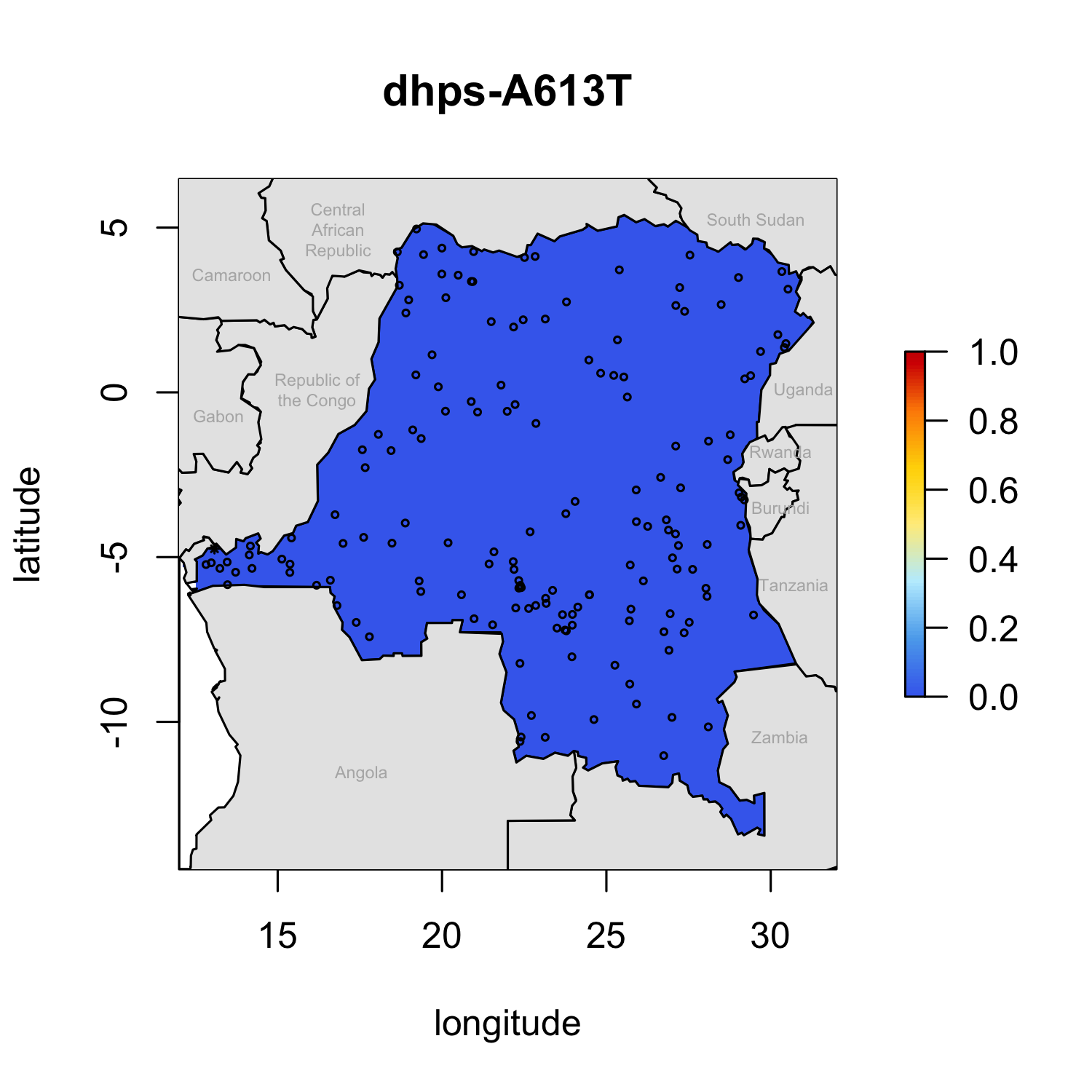** |
| --- | --- | --- | --- |
| **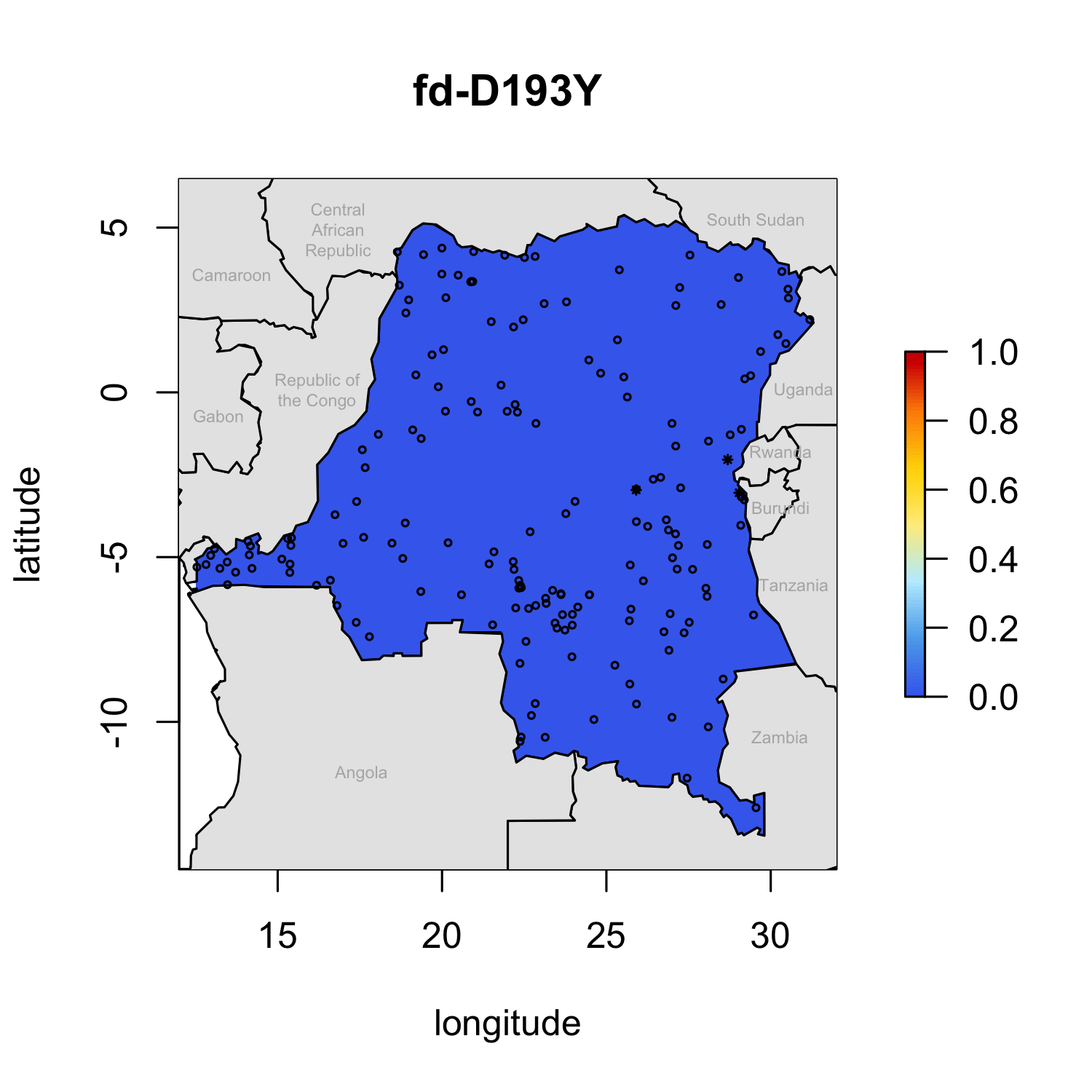** | **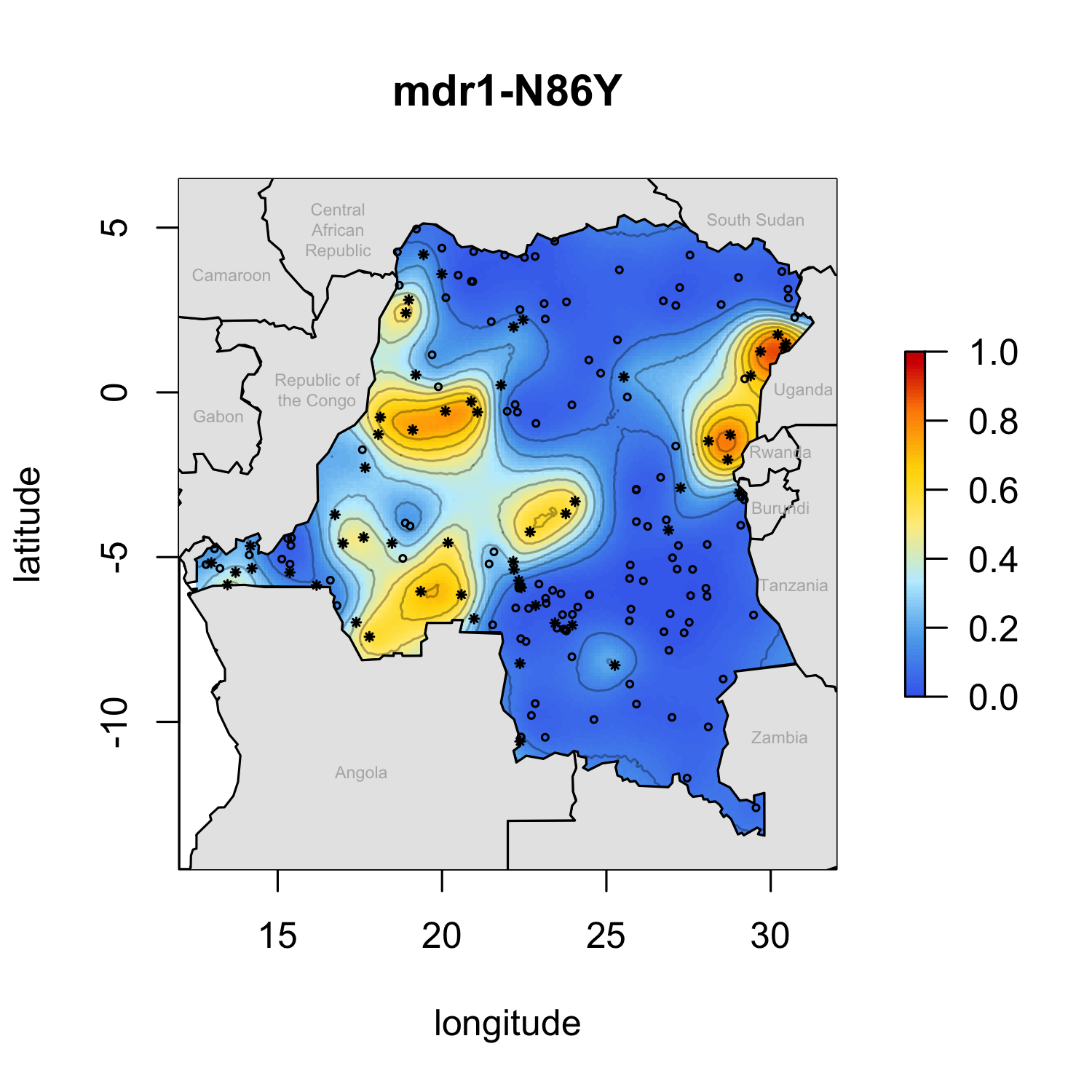** | **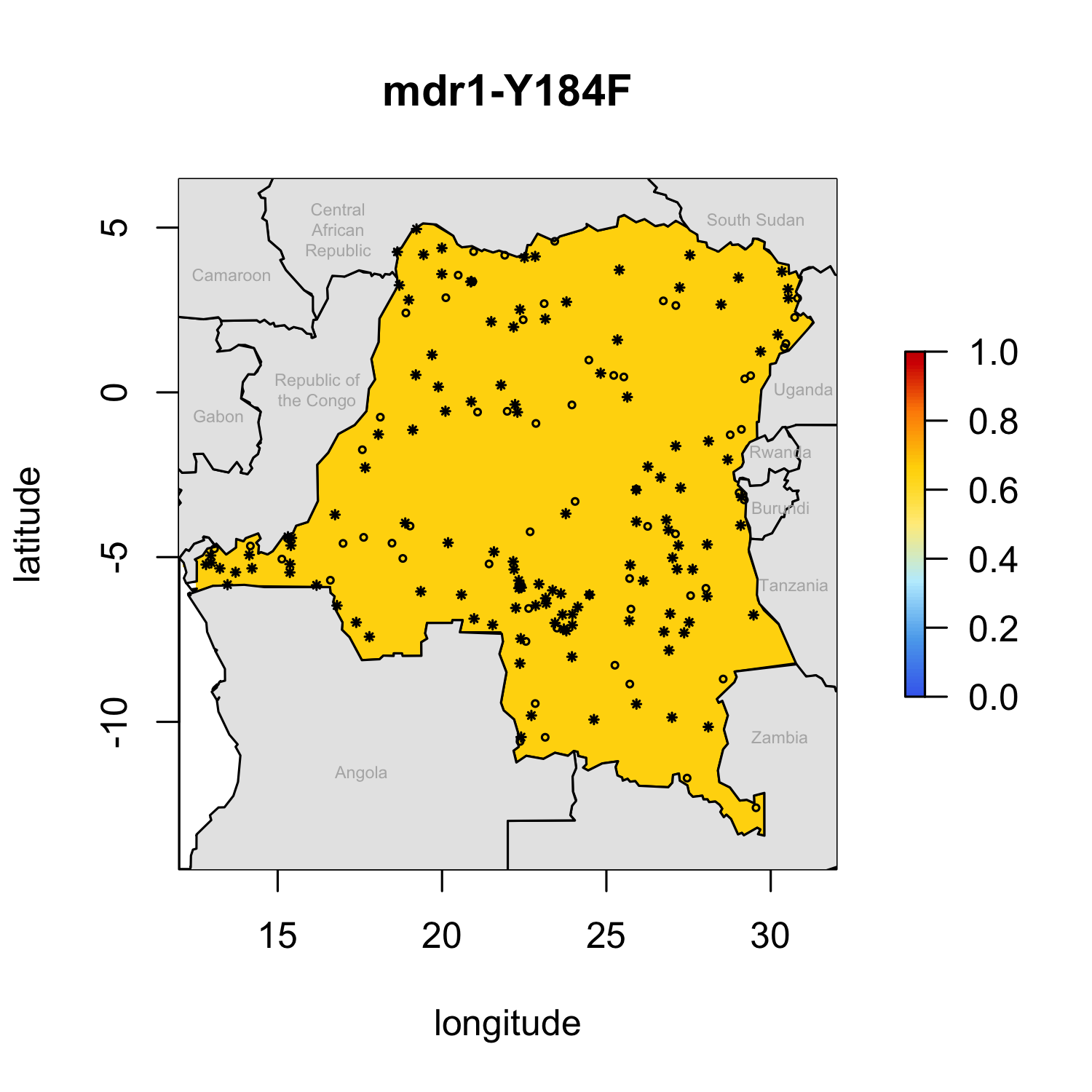** | **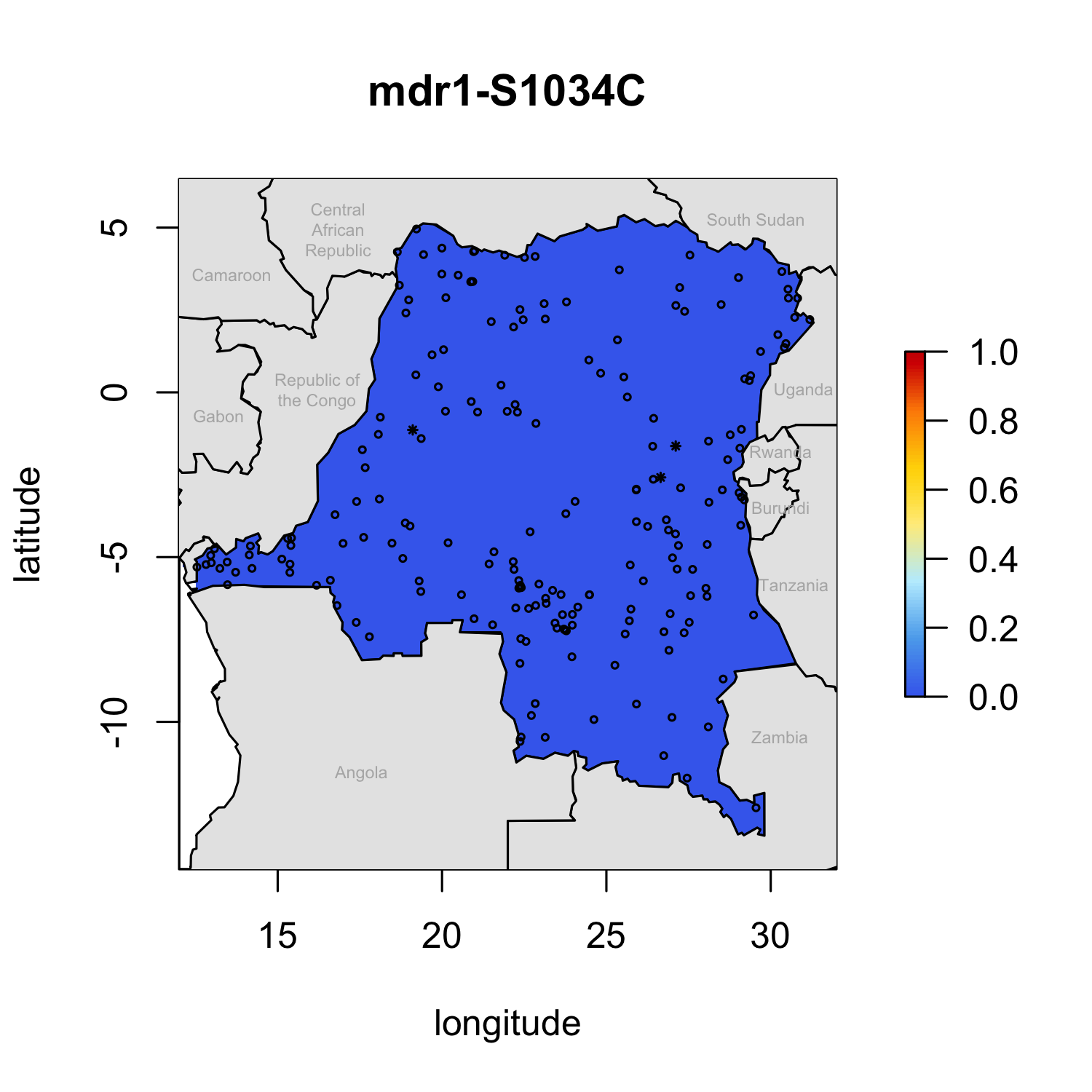** |
| **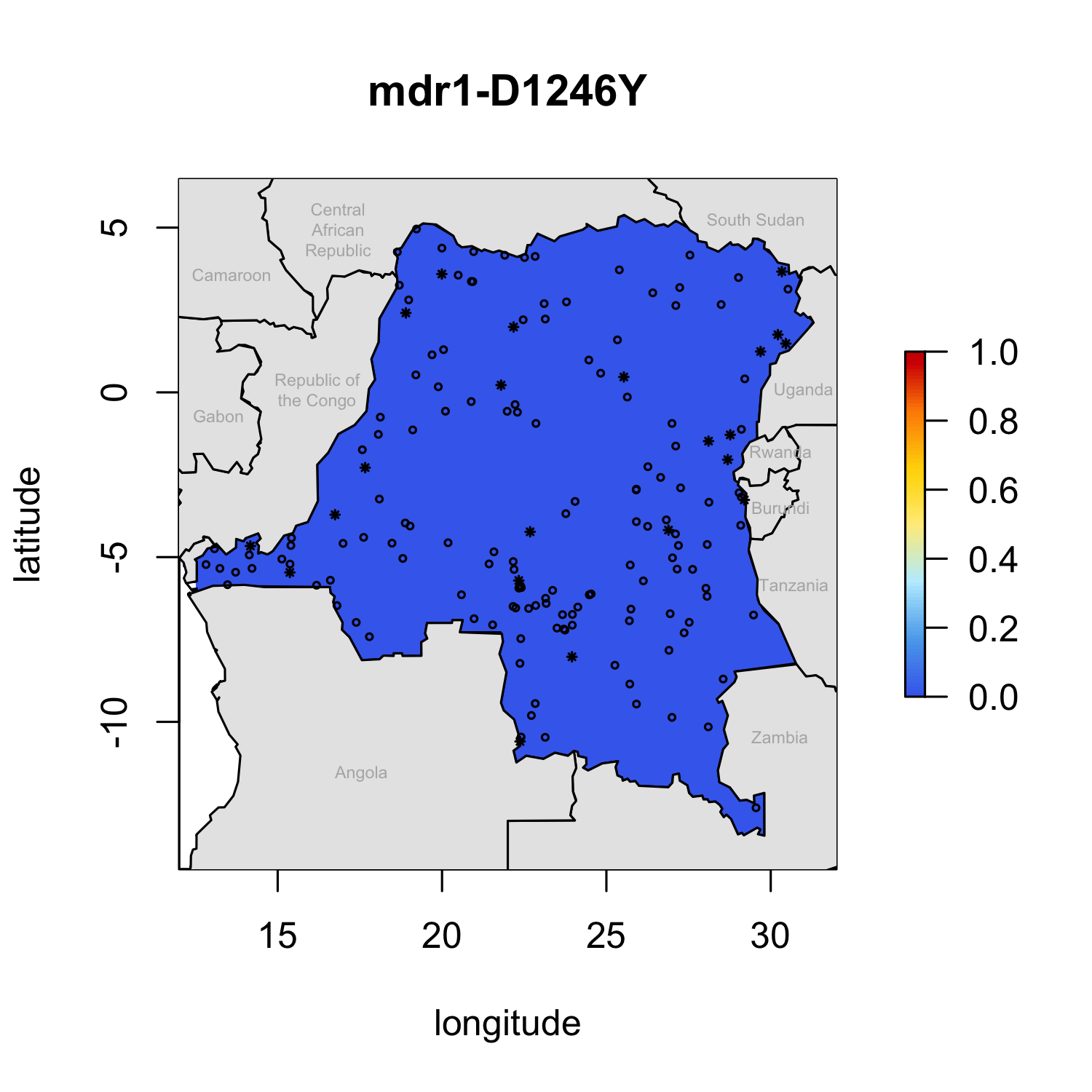** |  |  |  |
|  |  |  |  |

### ***Supplementary Figure 11. Predicted prevalence across DRC of the drug resistance mutations***

For each known or candidate drug resistance locus, the prevalence of the resistant allele mapped across the DRC as predicted by PrevMap.
