# Supplementary material for: Drug-Resistance and Population Structure of Plasmodium falciparum Across the Democratic Republic of Congo Using High-Throughput Molecular Inversion Probes
Source: J Infect Dis. 2018 Apr 28;218(6):946–55. doi: 10.1093/infdis/jiy223 (PMC6093412; doi:10.1093/infdis/jiy223)
Supplement: Supplementary Figure12 [file jiy223_suppl_supplementary_figure12.docx]

| 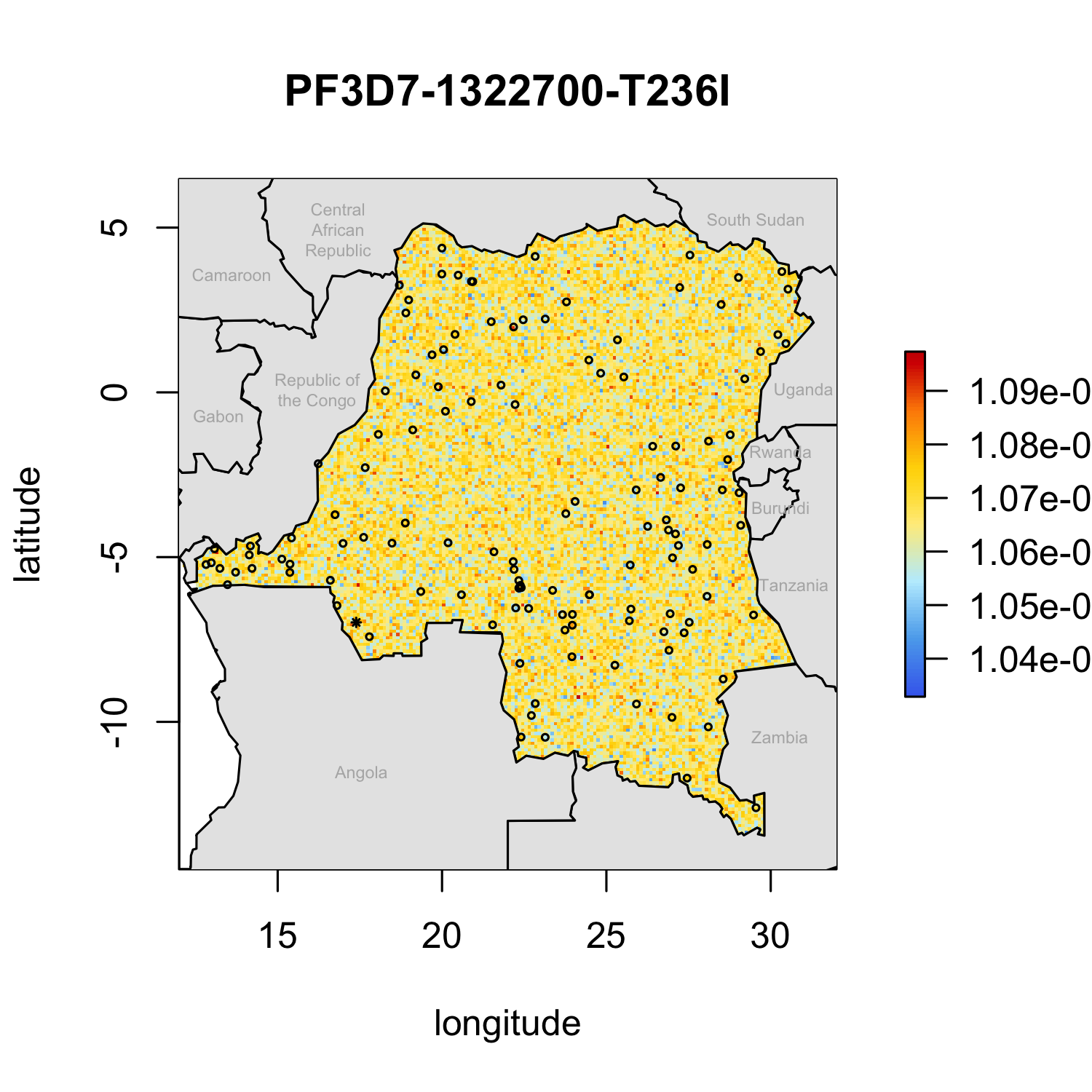 | 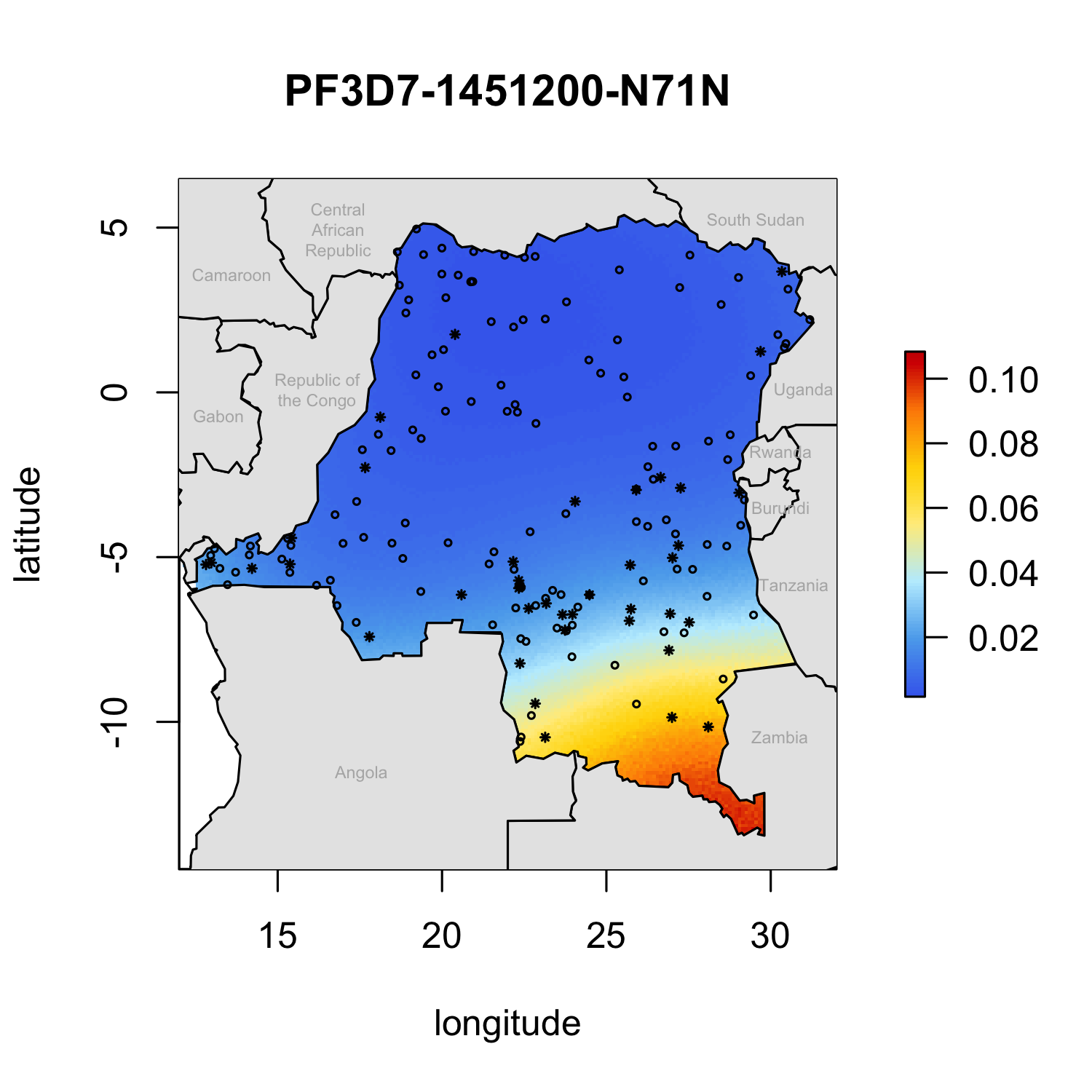 | 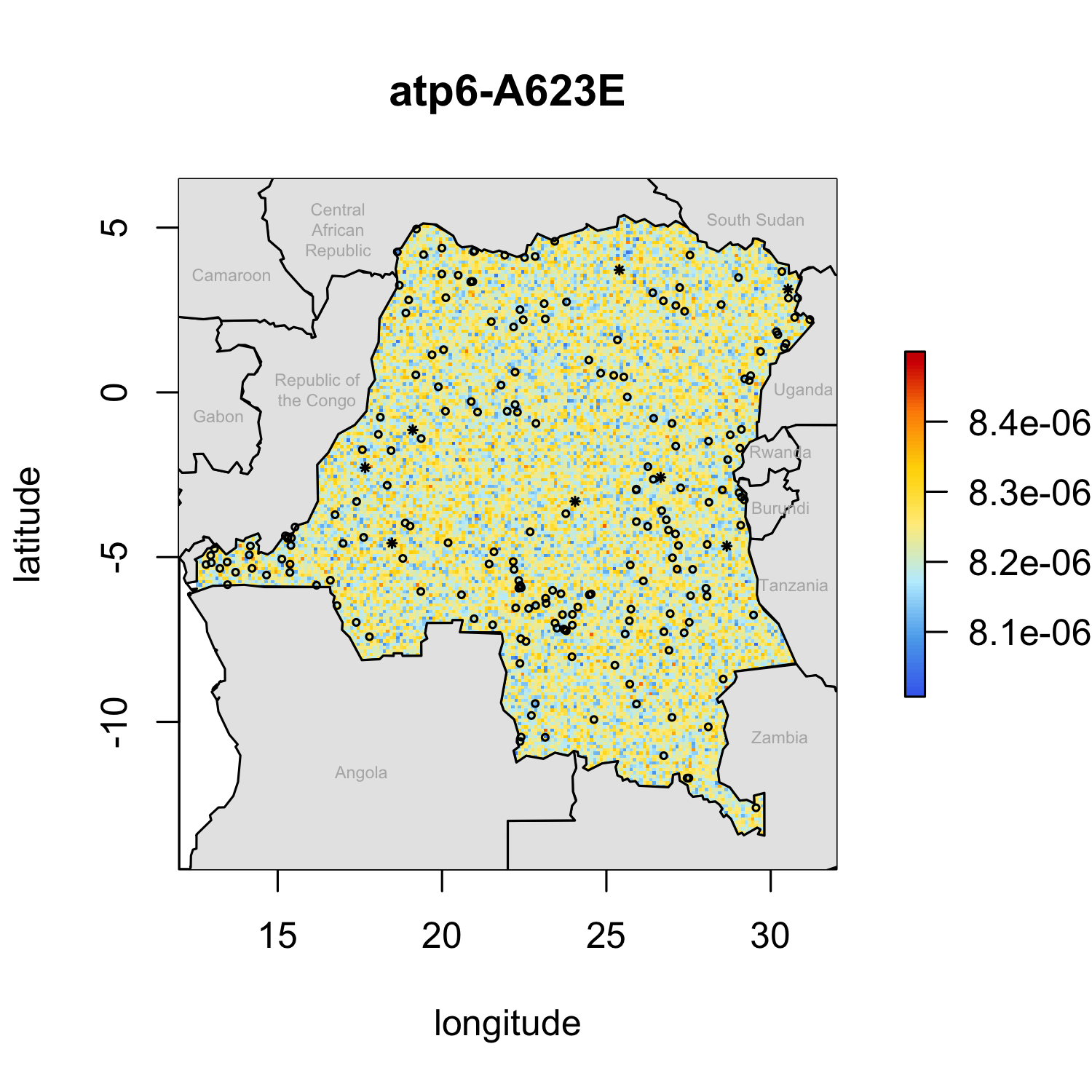 | 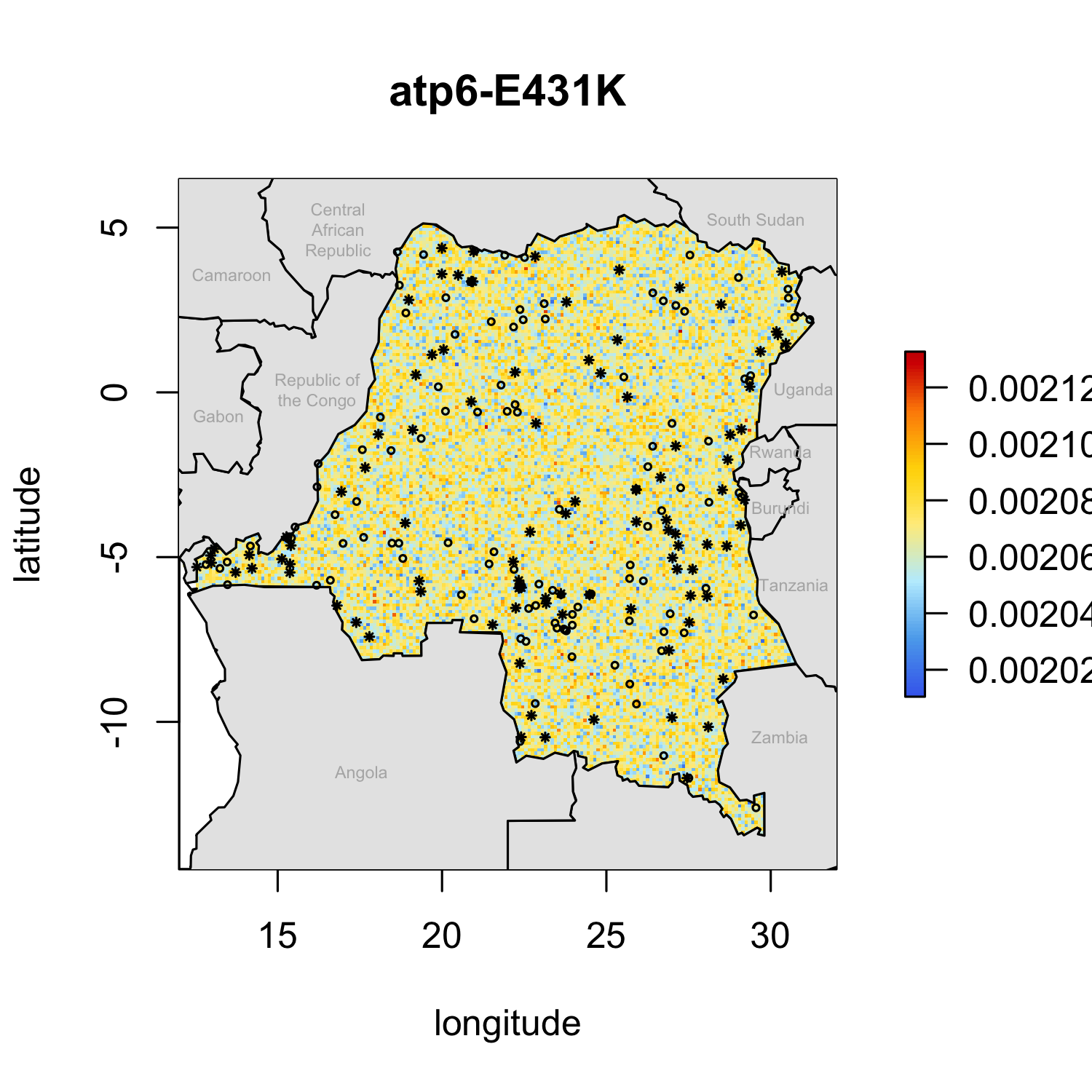 |
| --- | --- | --- | --- |
| 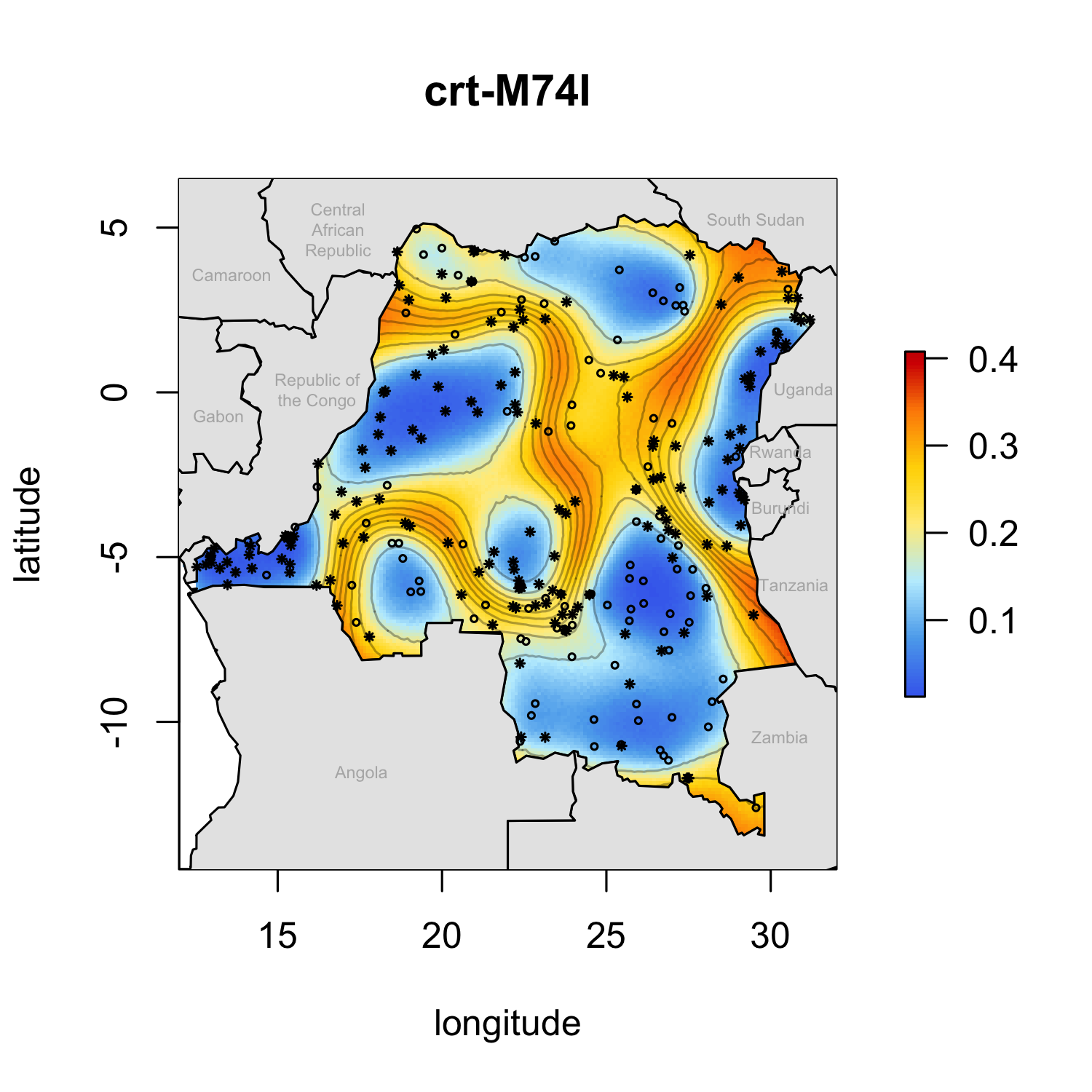 | 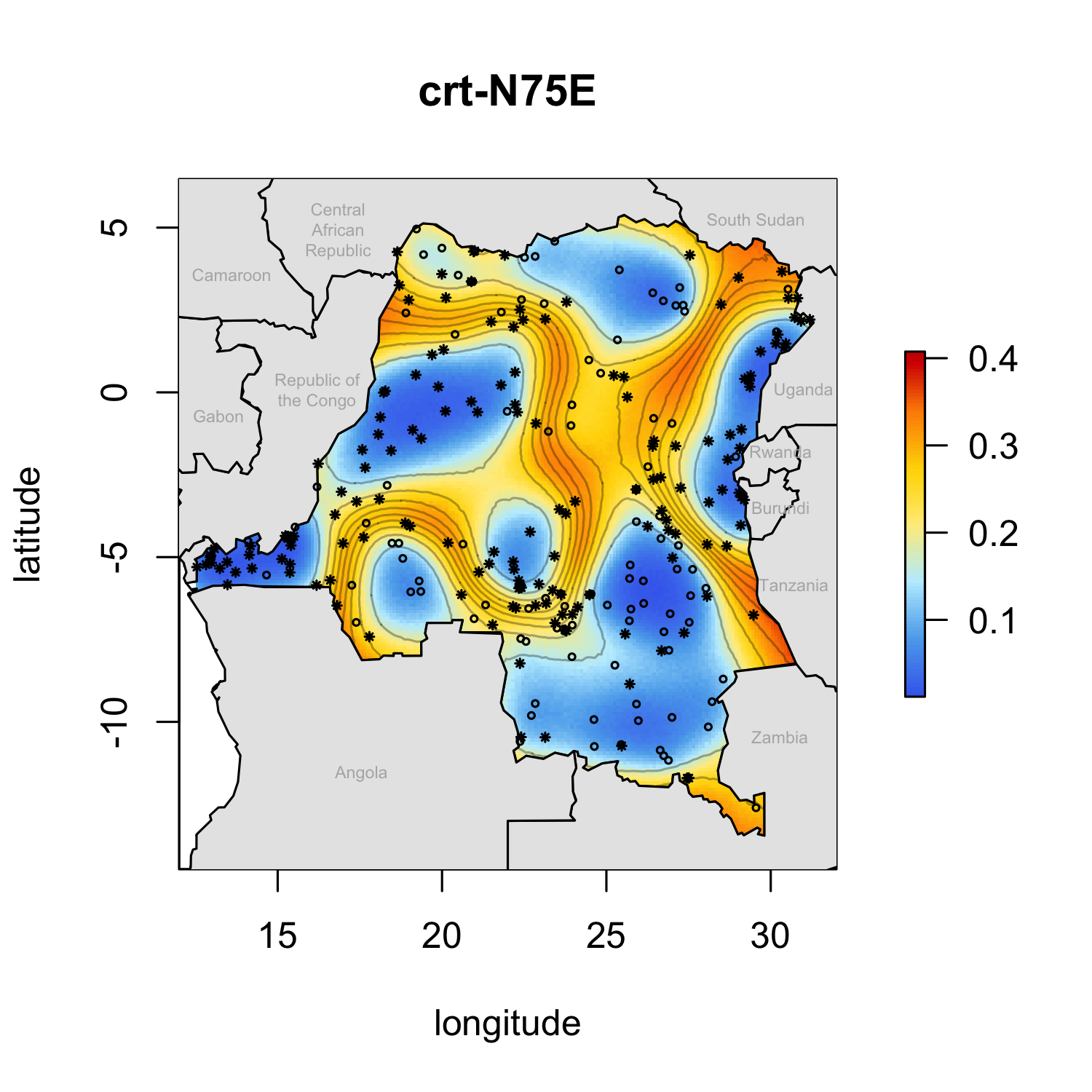 | 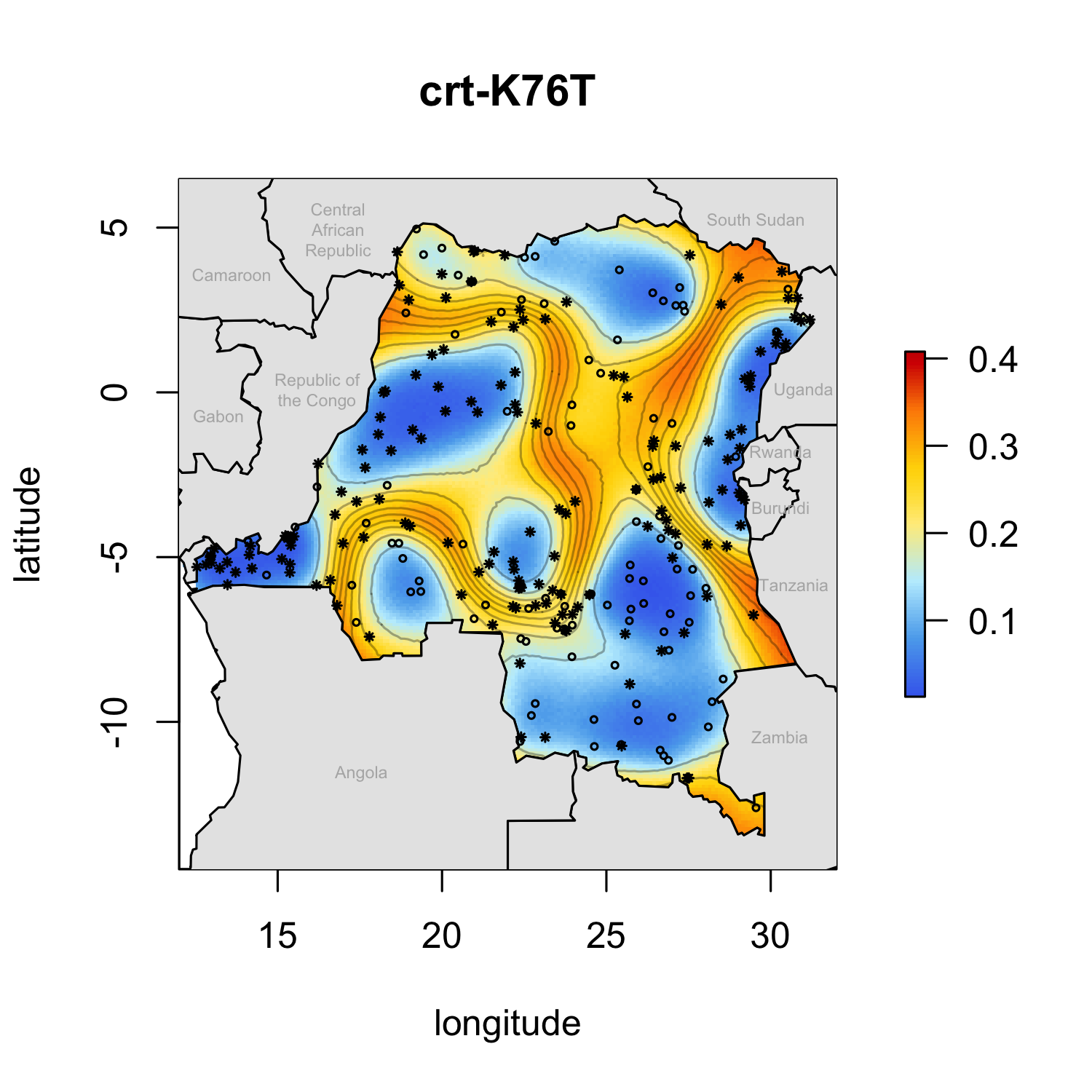 | 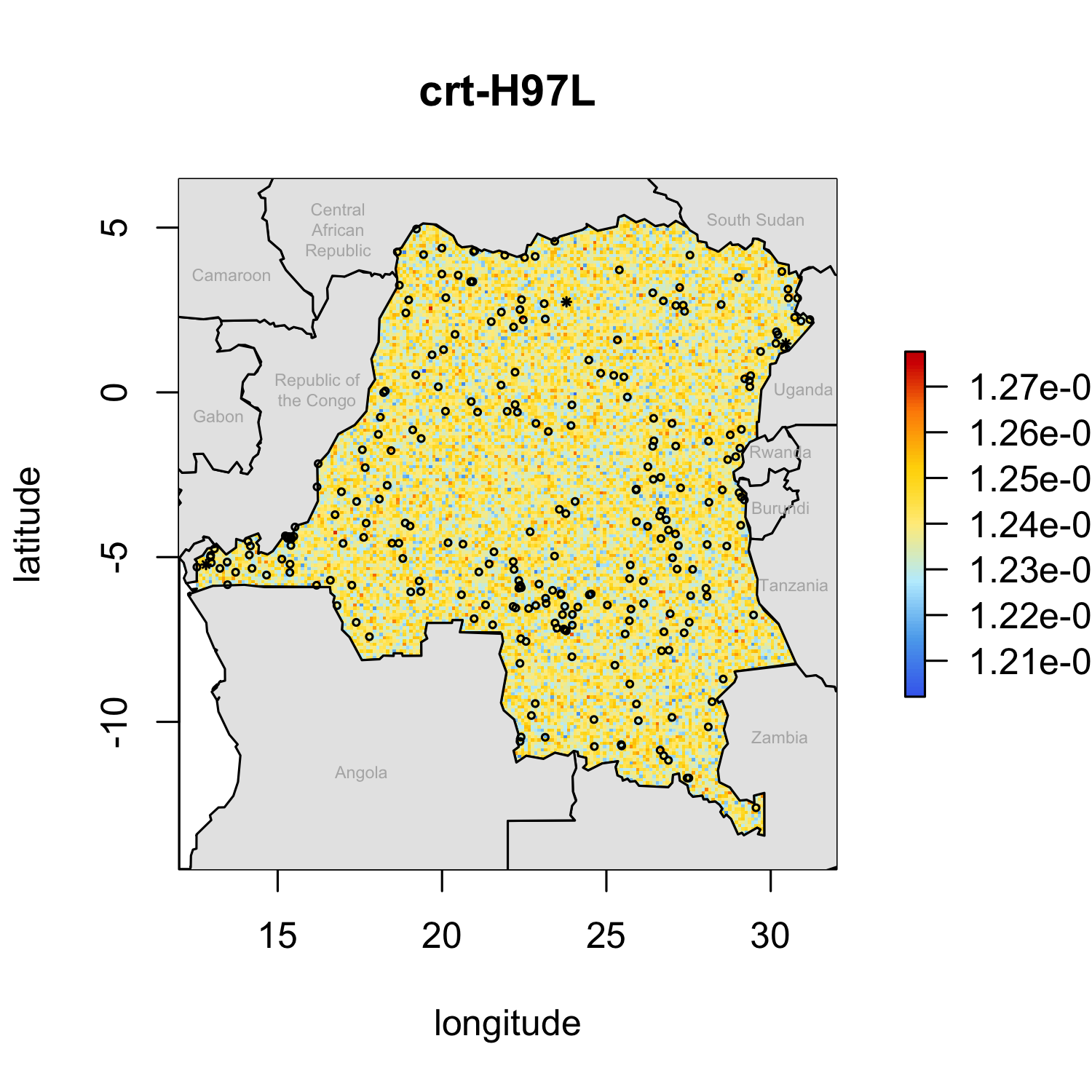 |
| 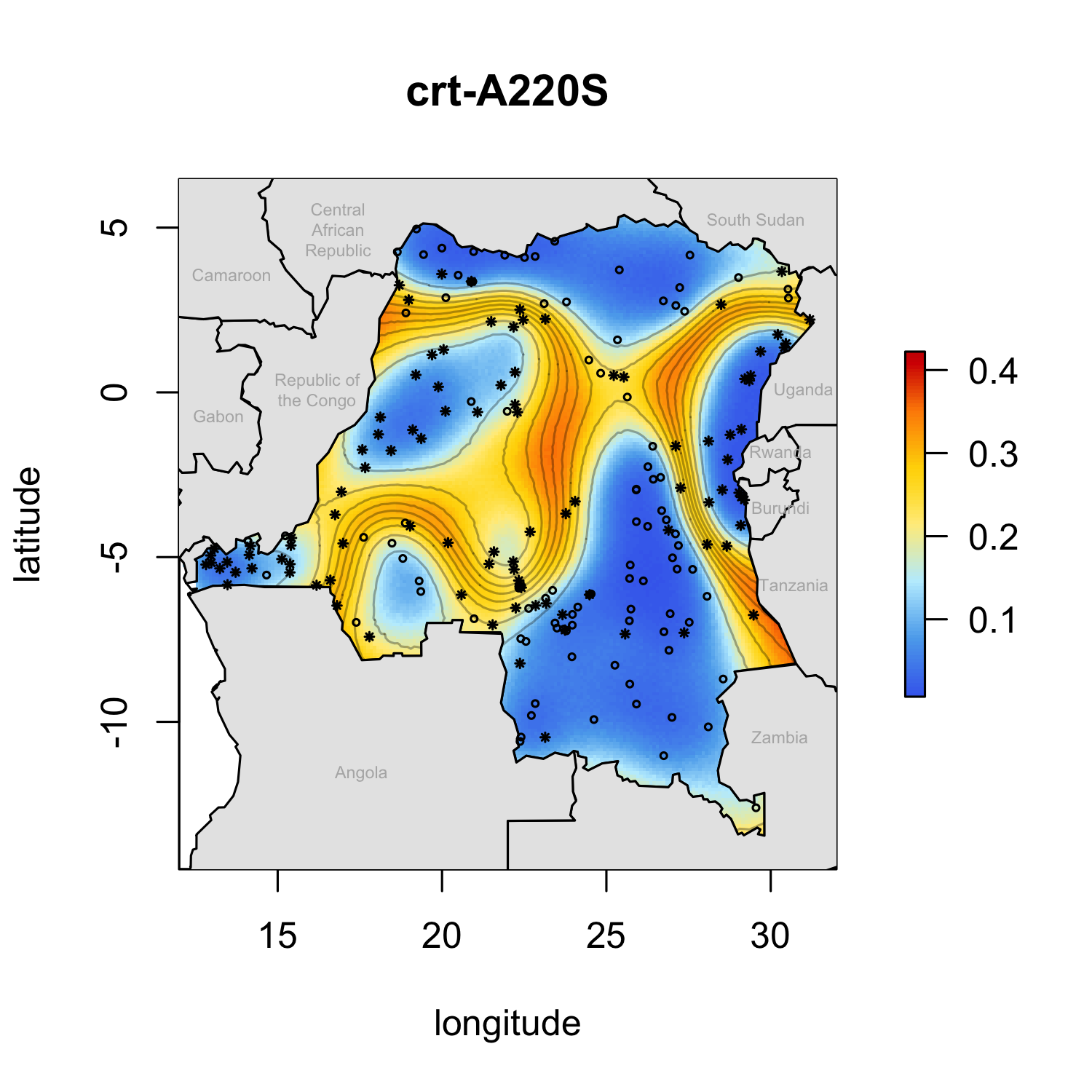 | 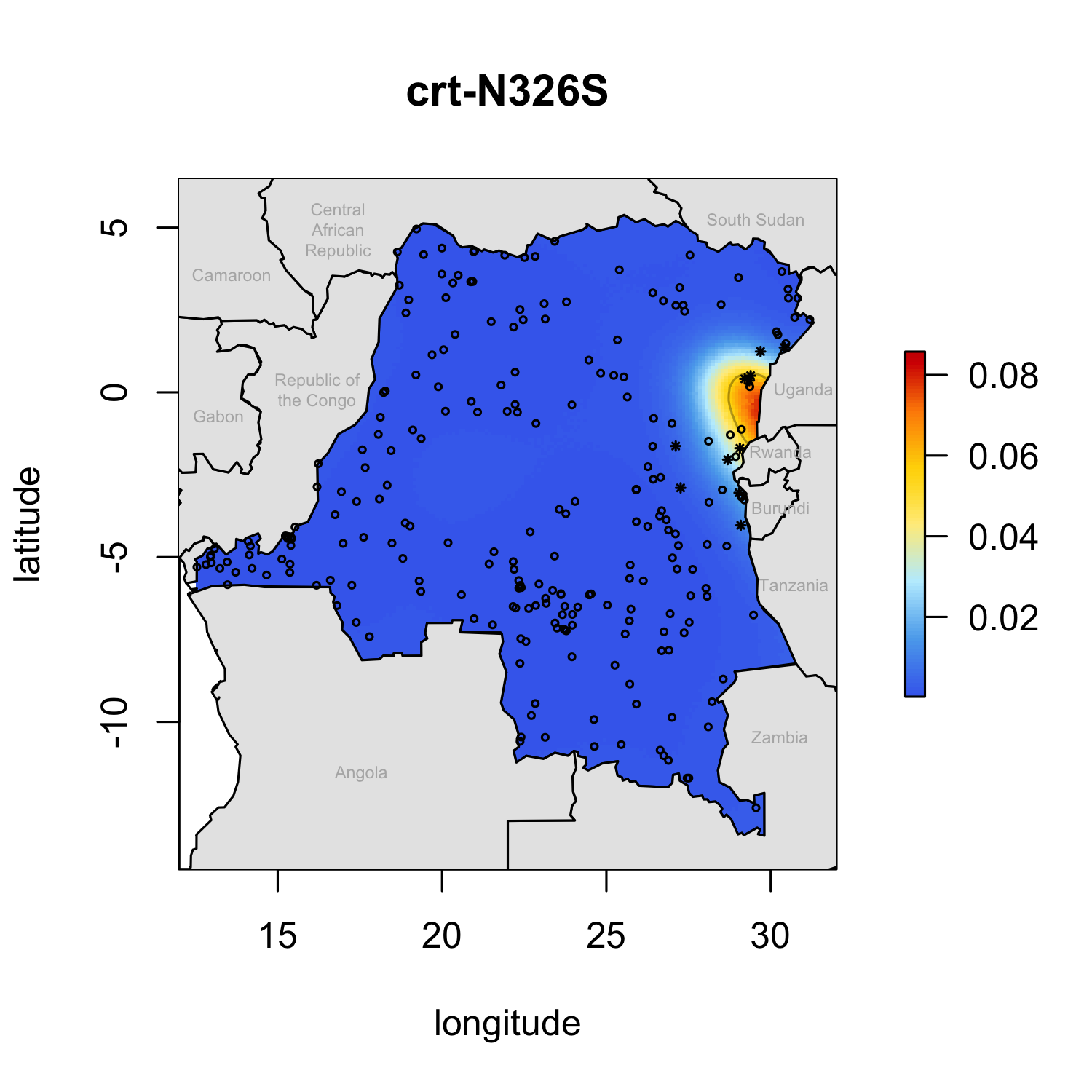 | 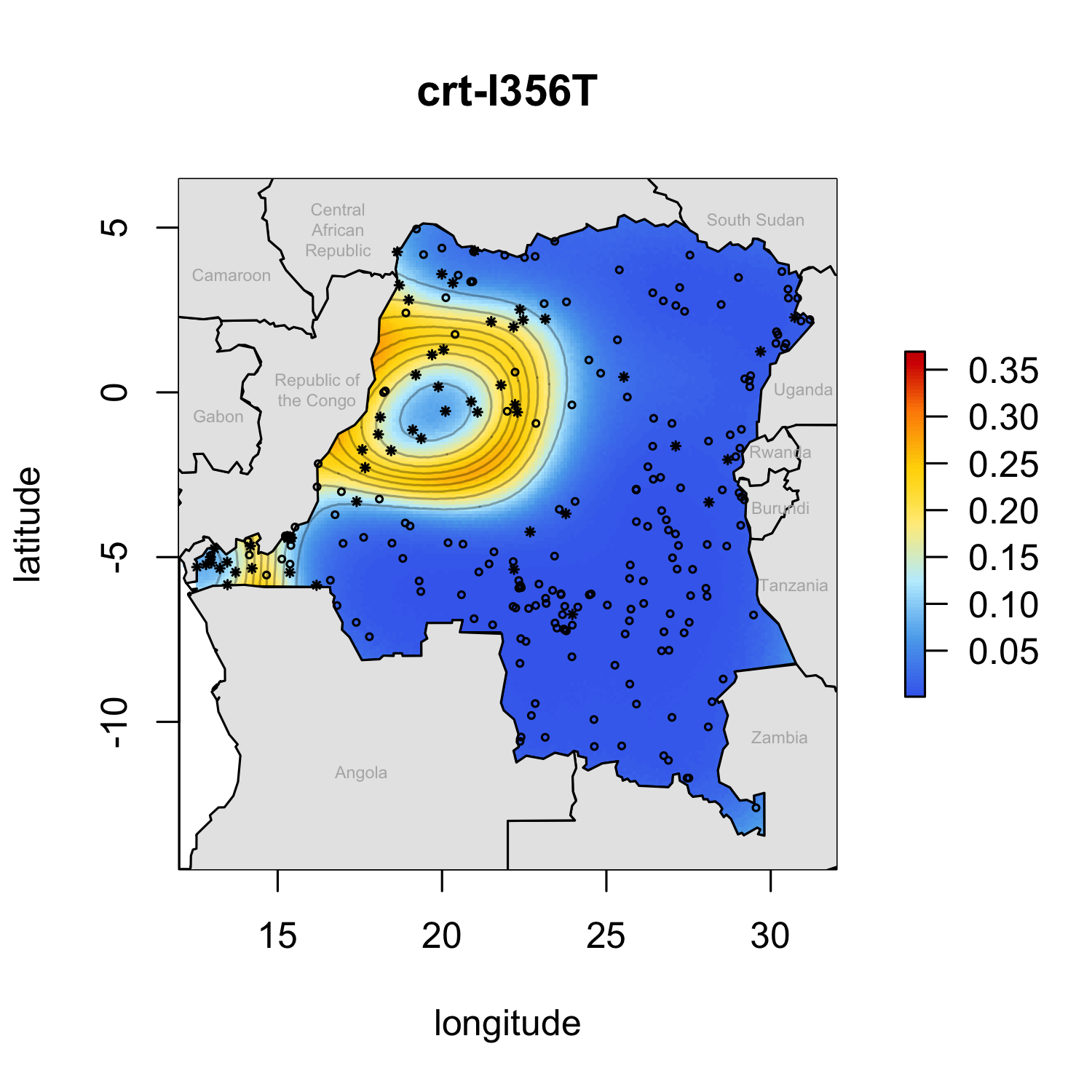 | 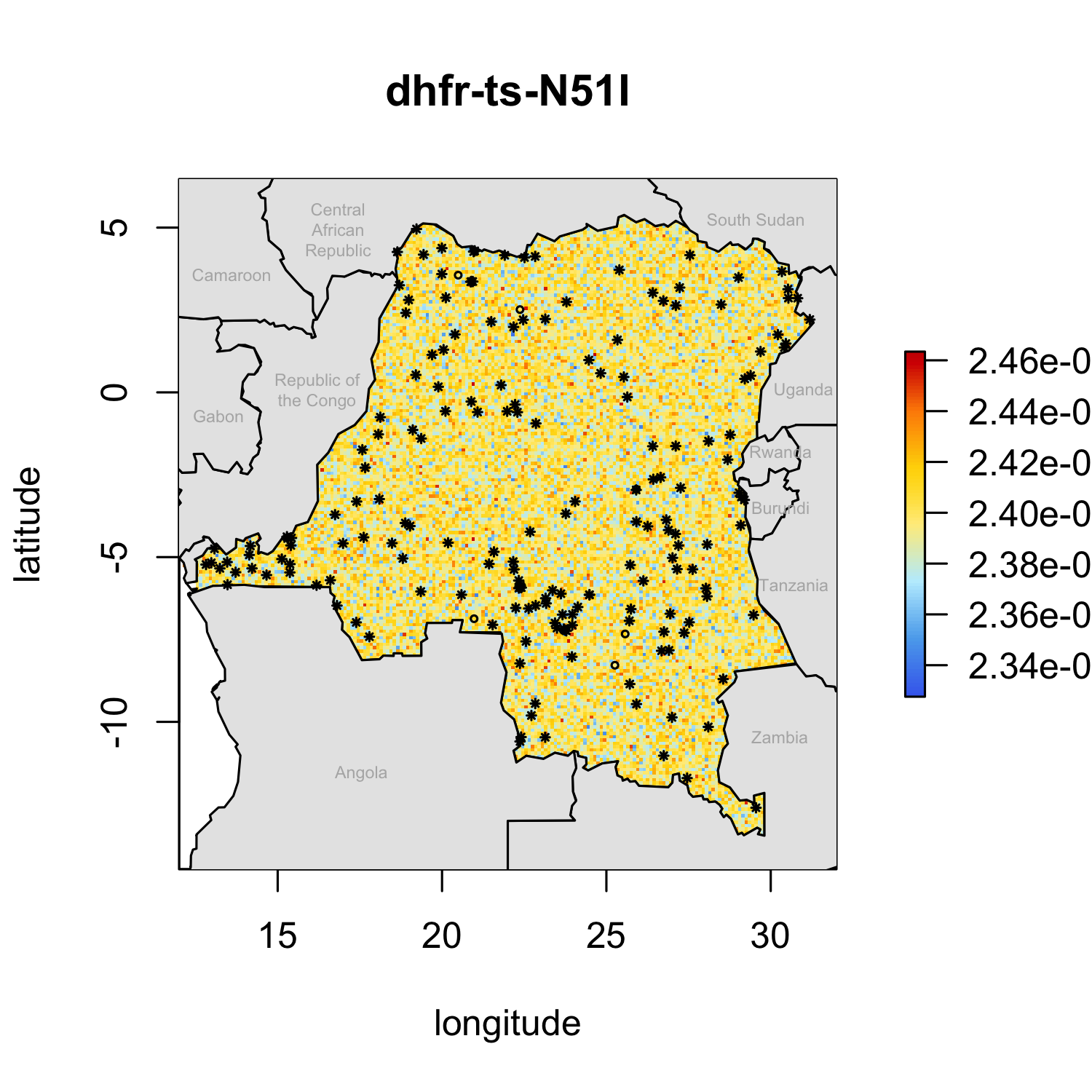 |
| 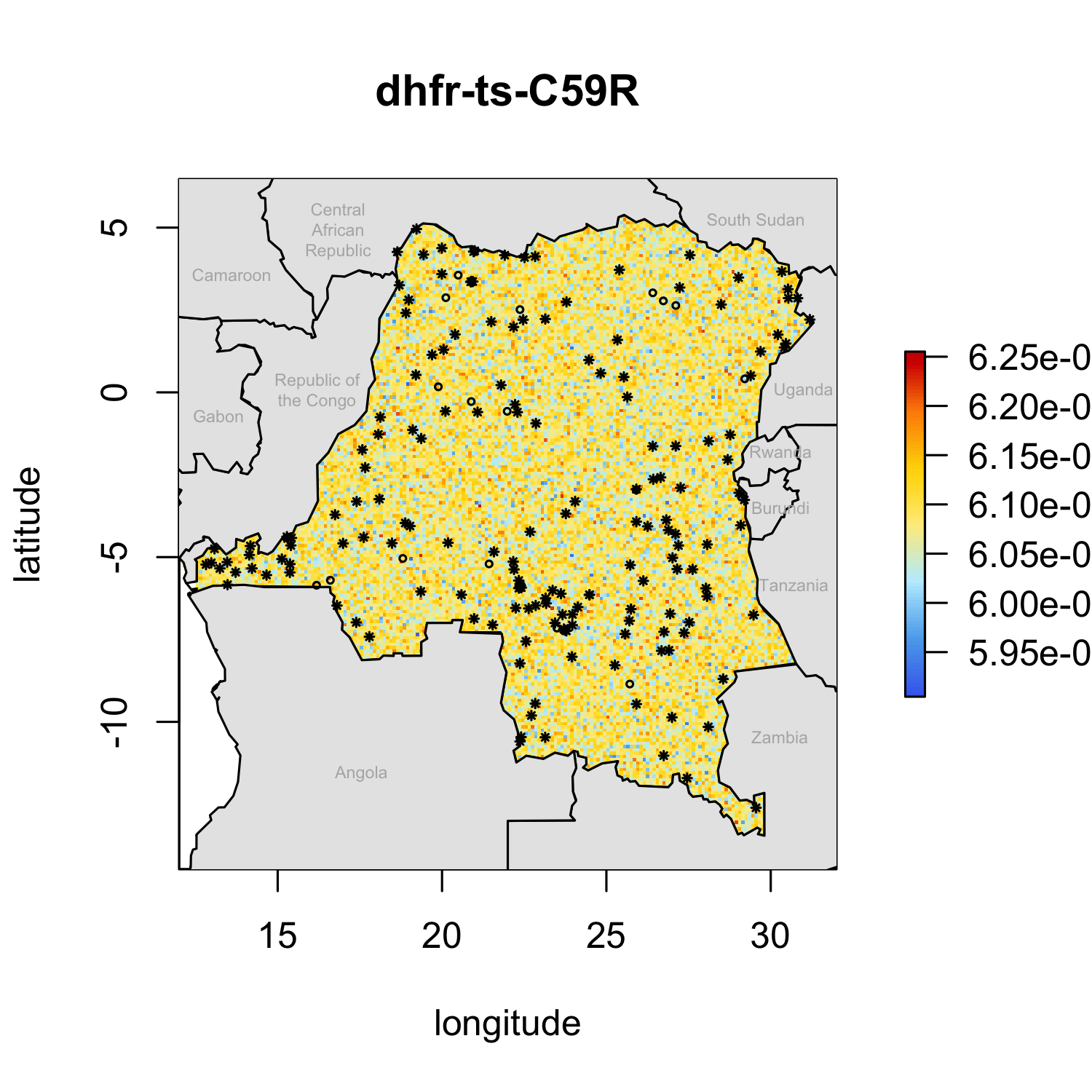 | 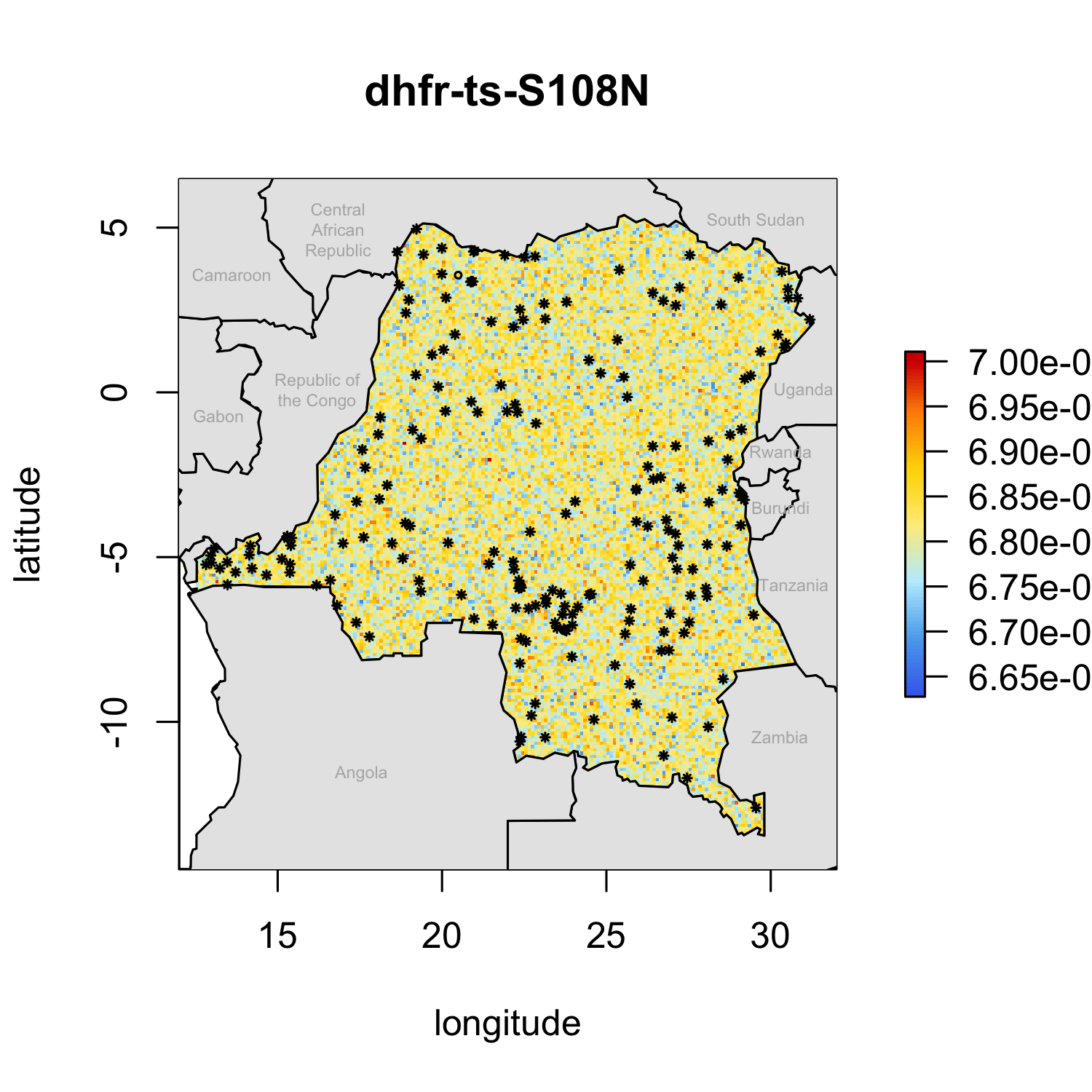 | 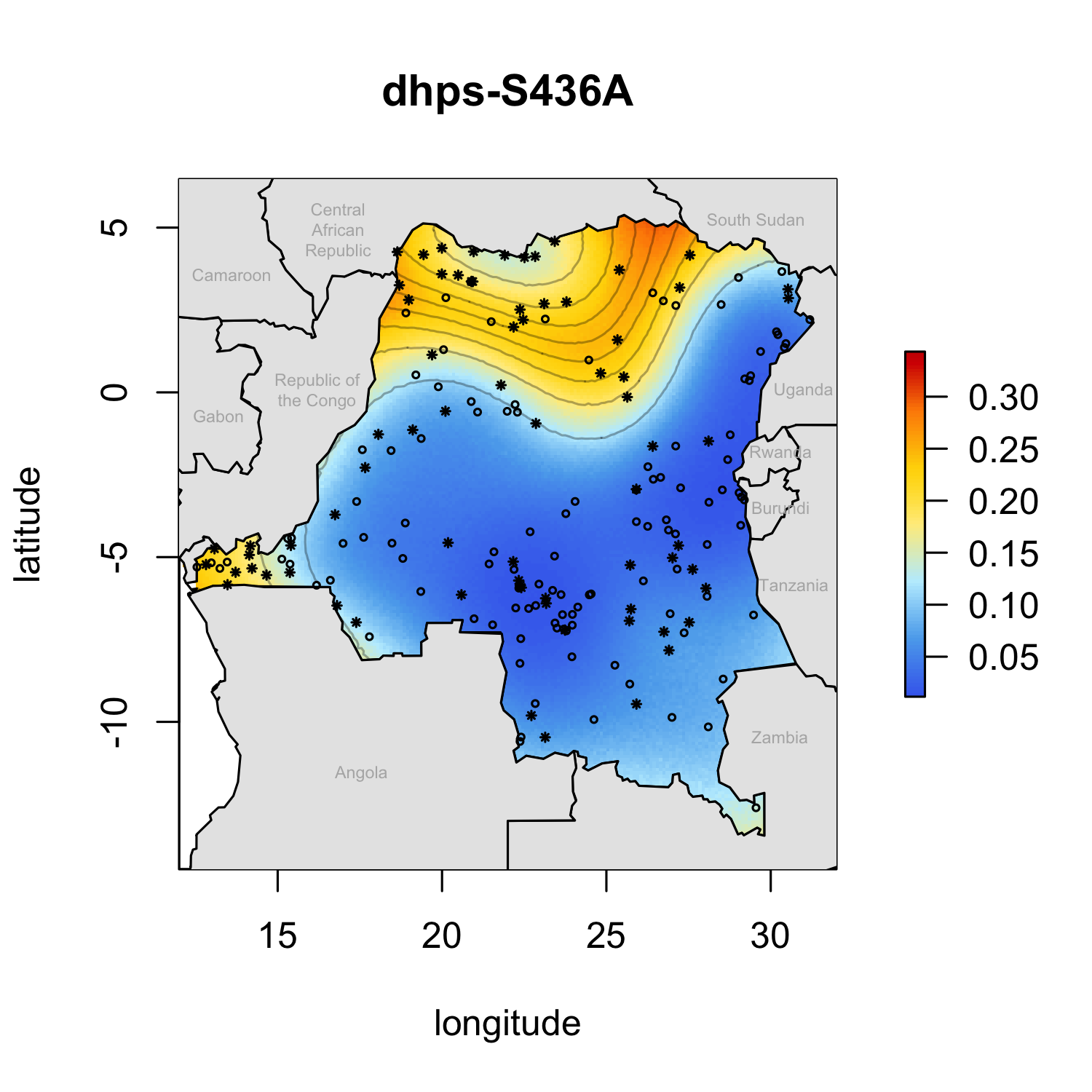 | 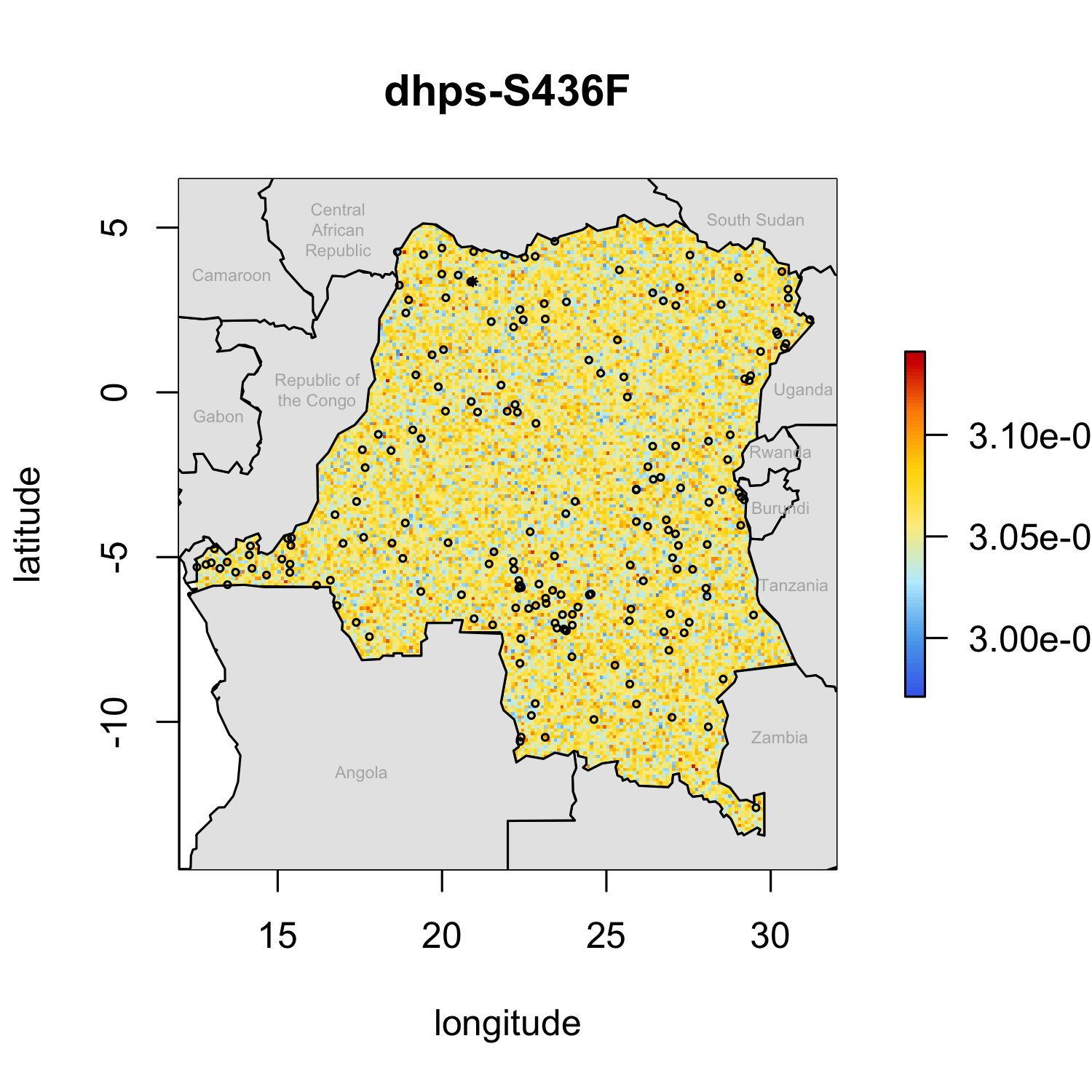 |

| 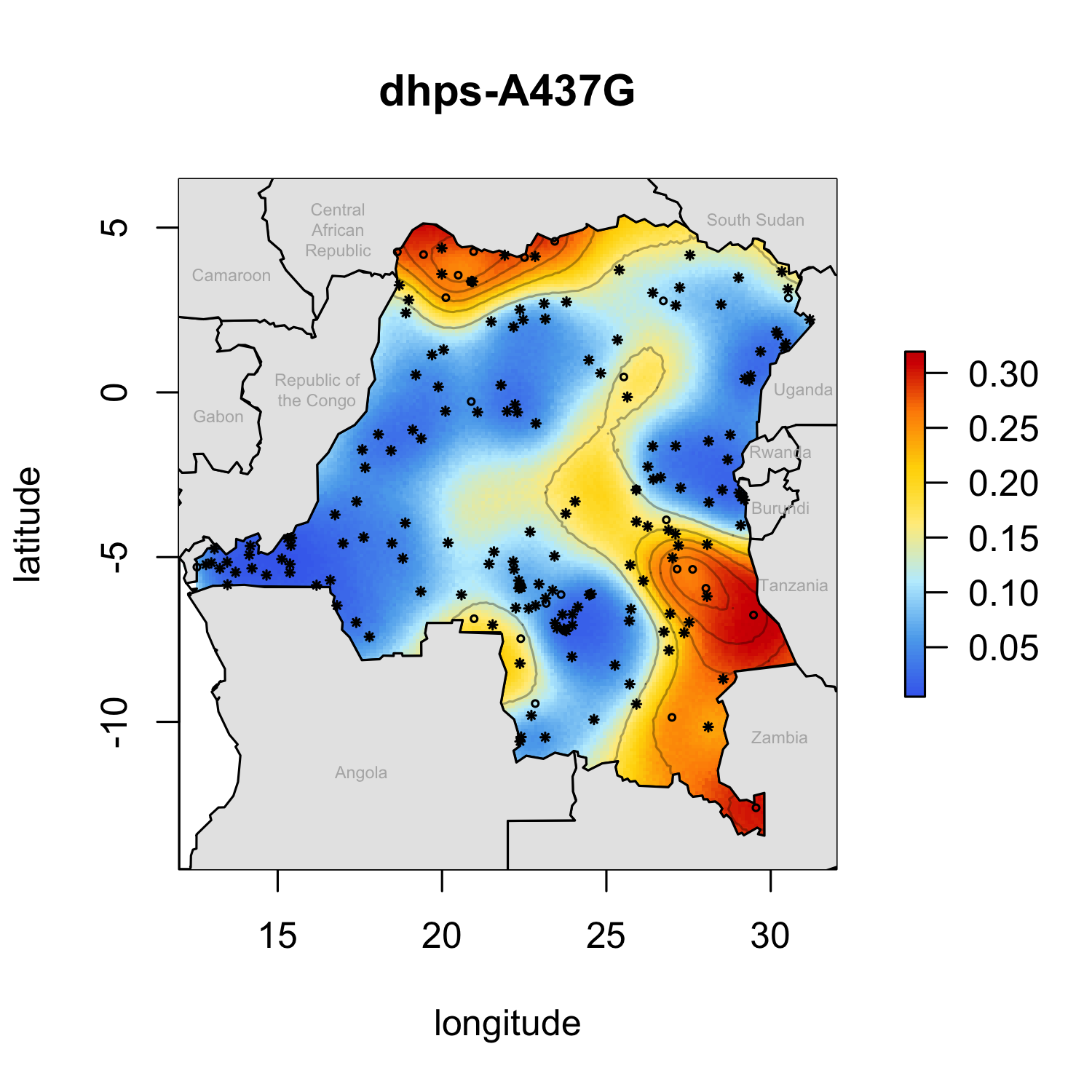 | 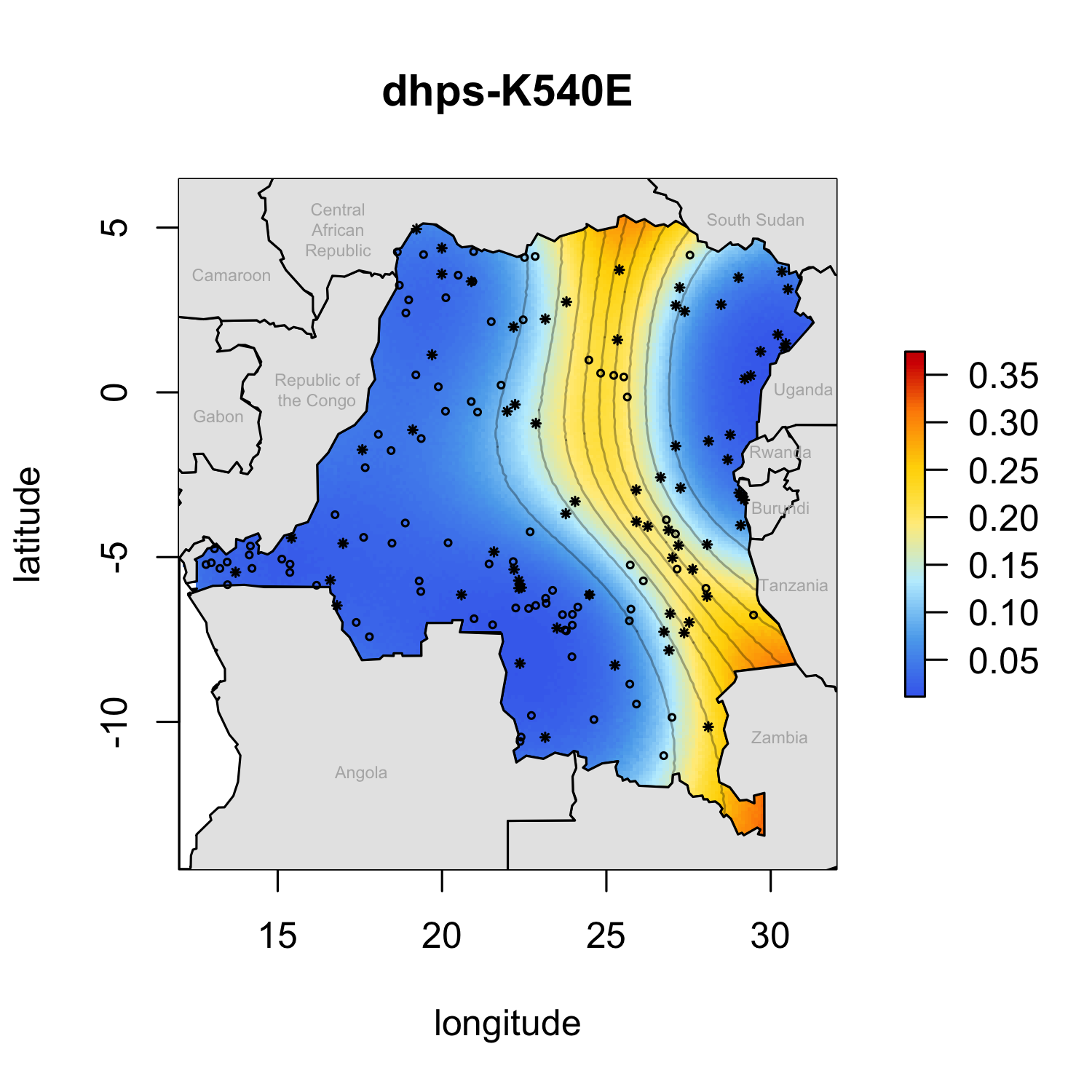 | 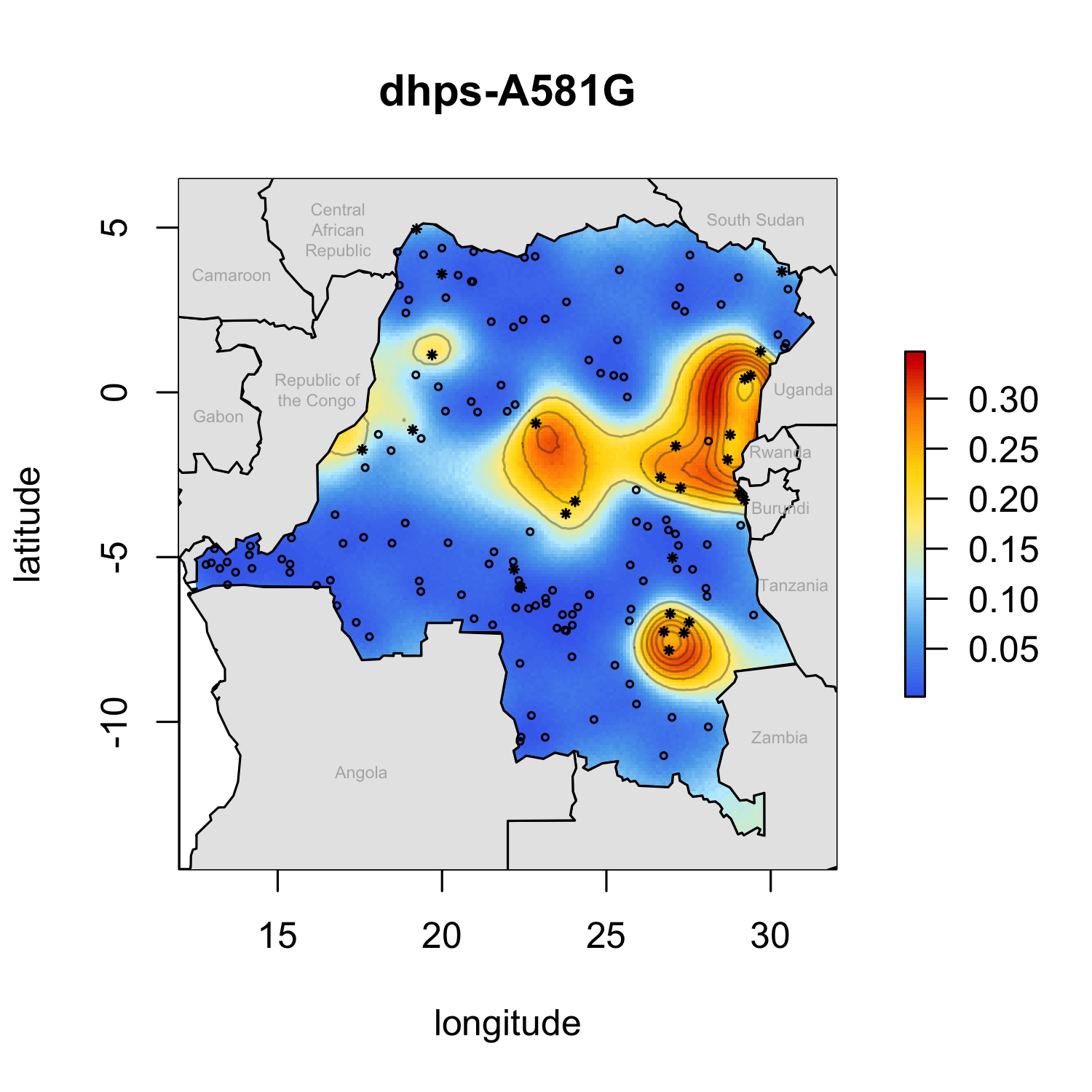 | 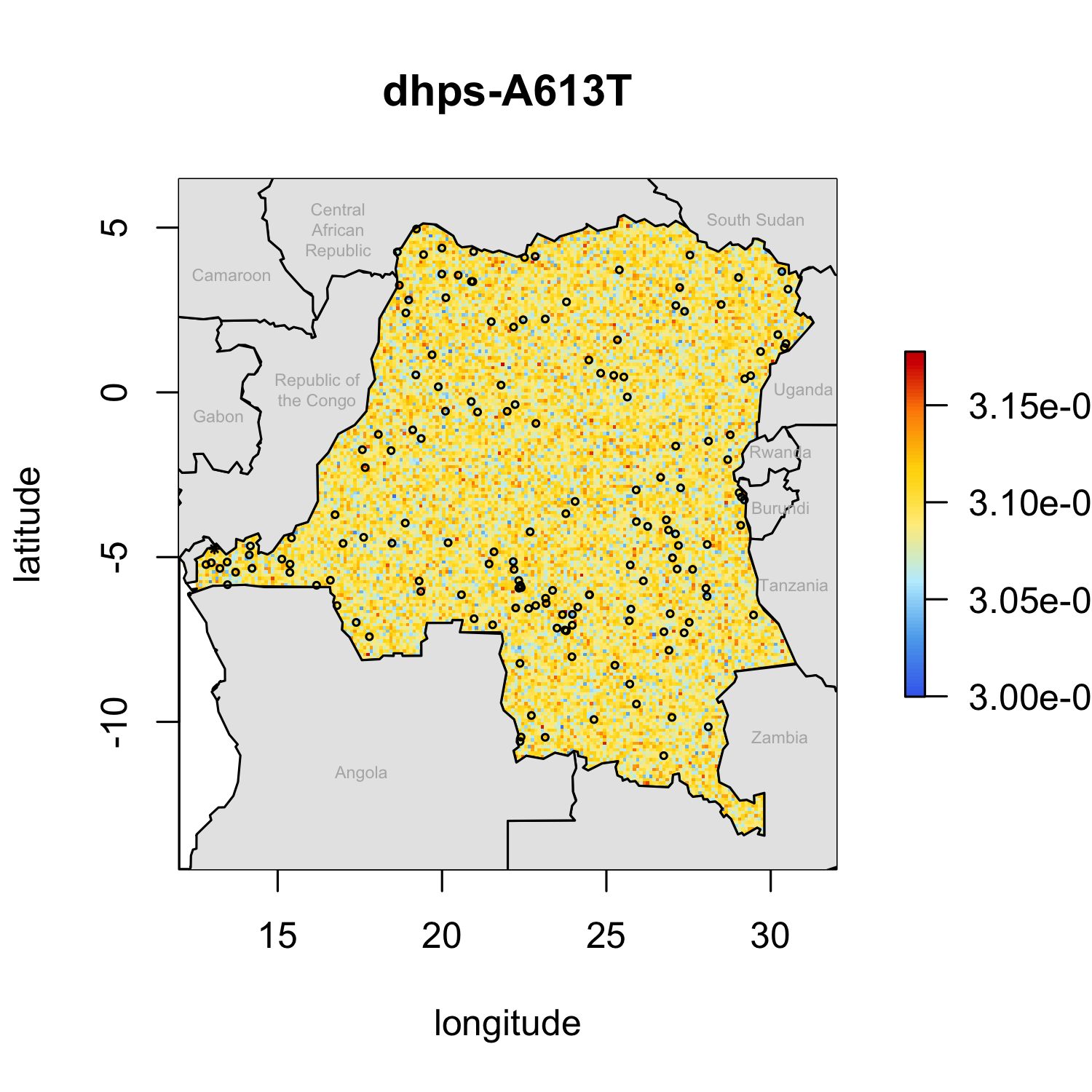 |
| --- | --- | --- | --- |
| 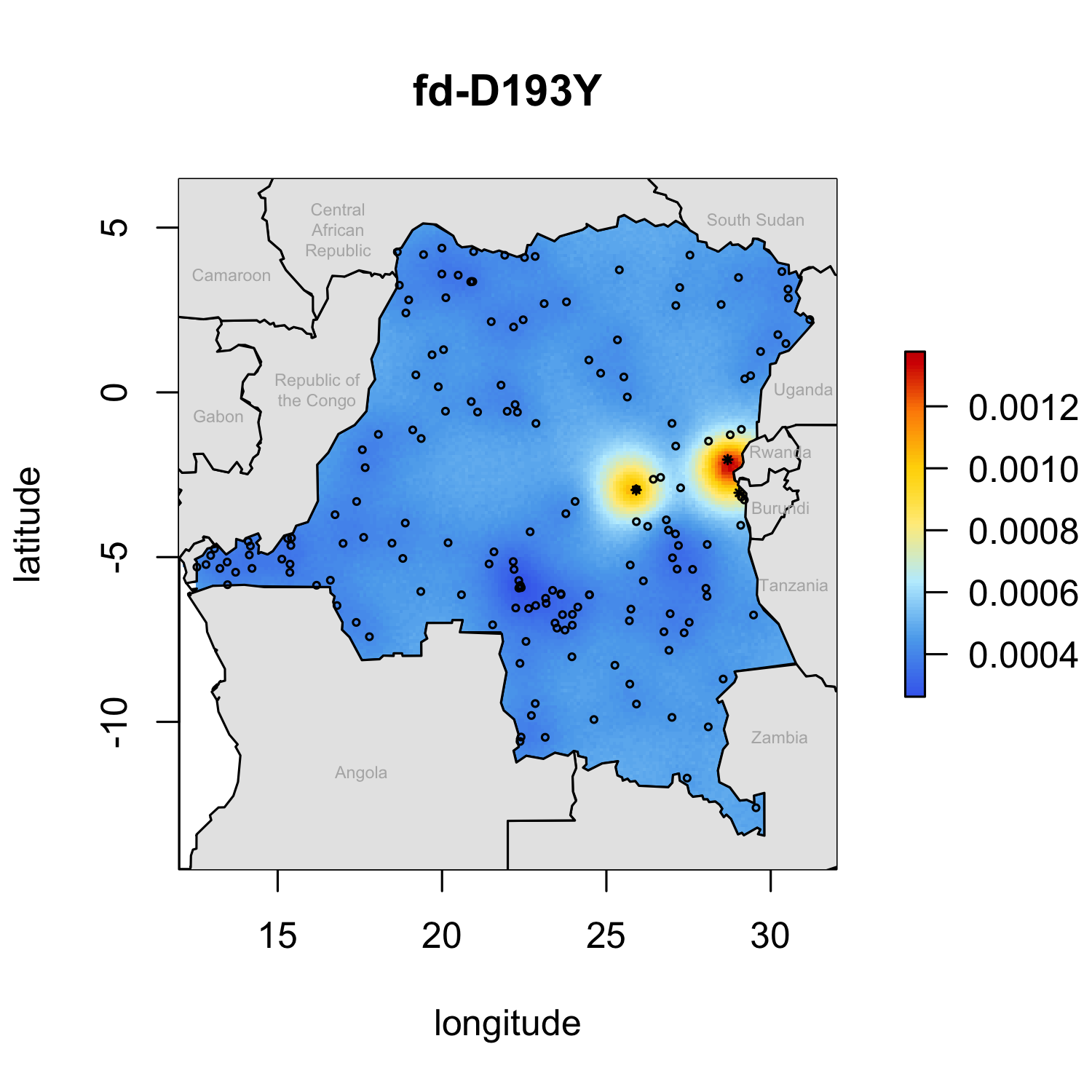 | 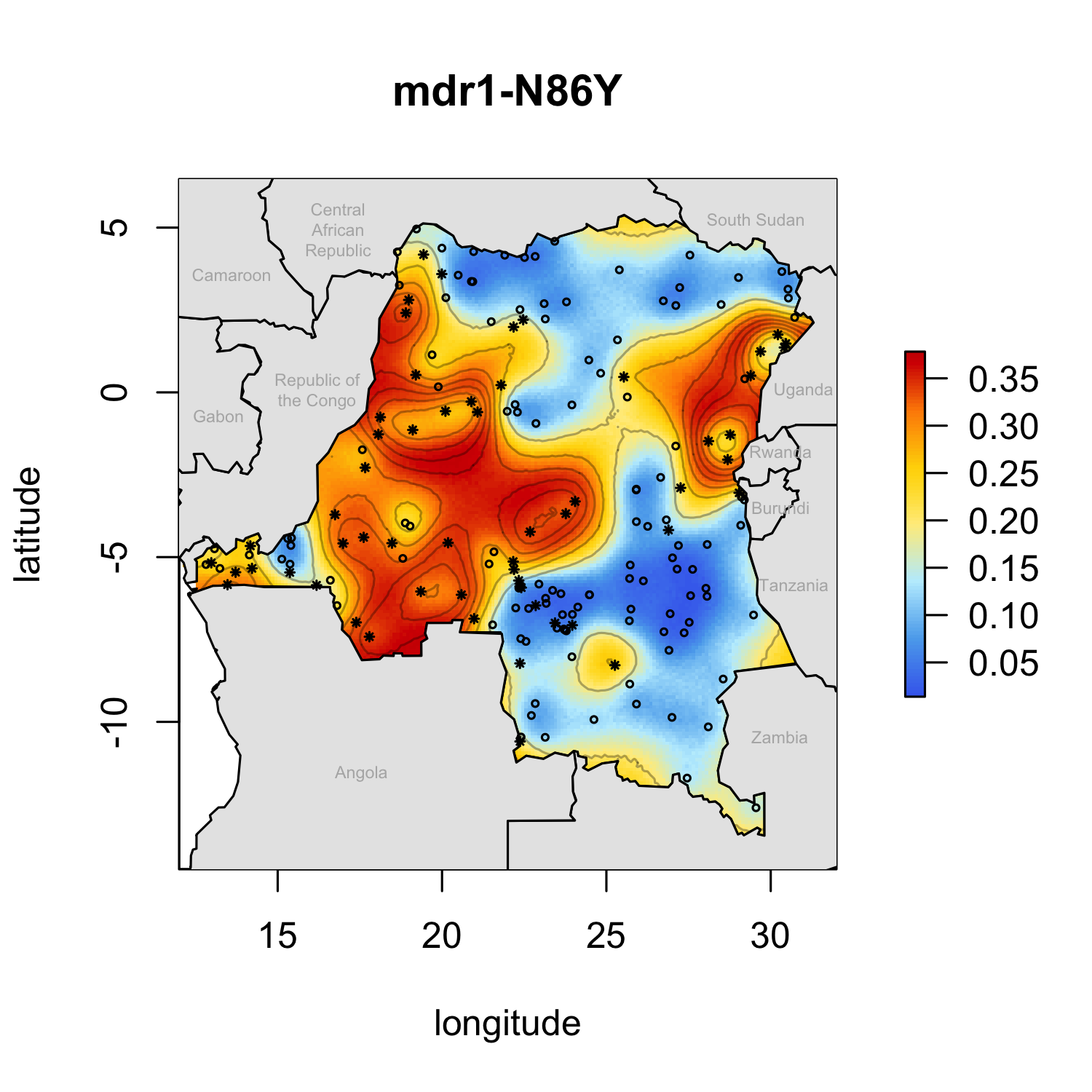 | 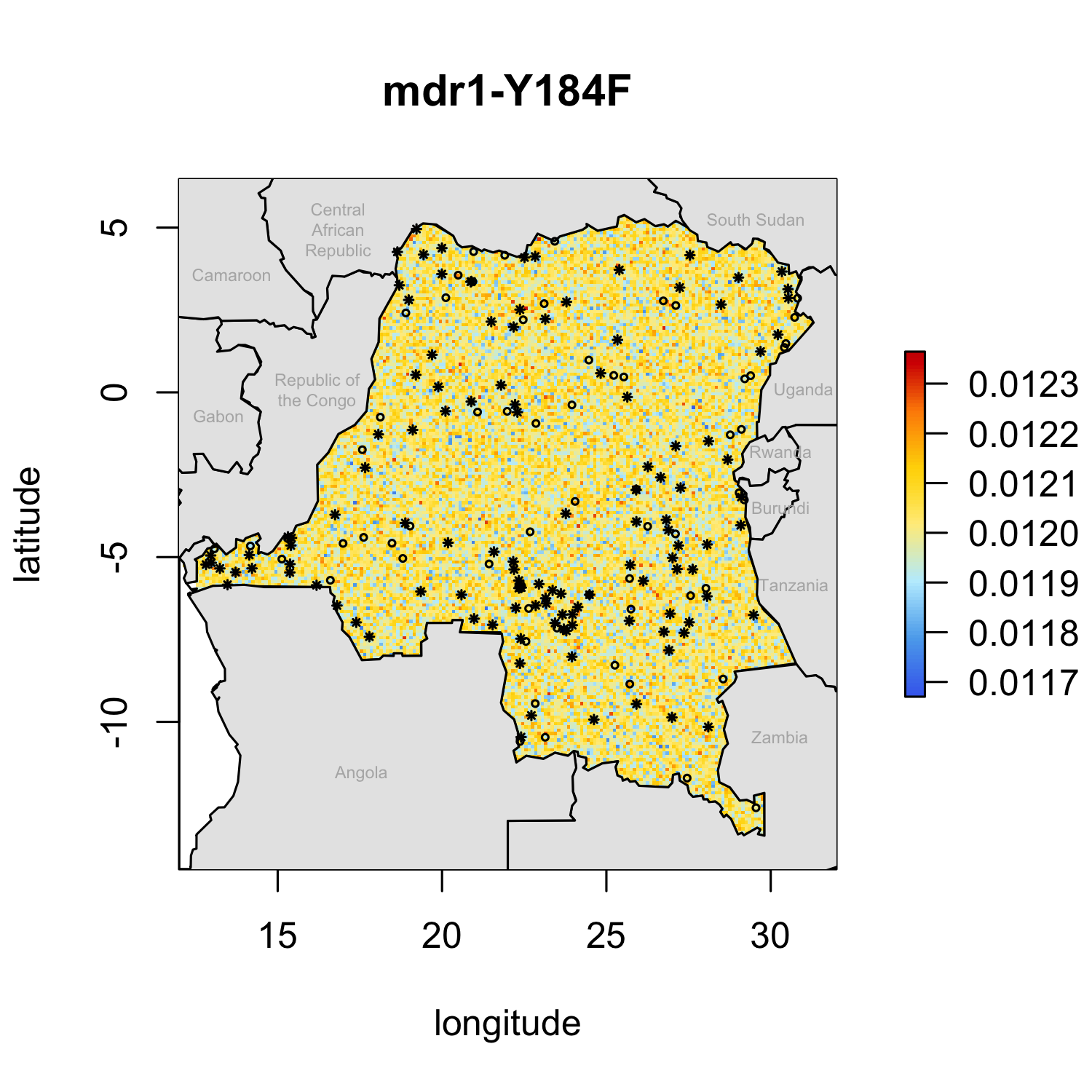 | 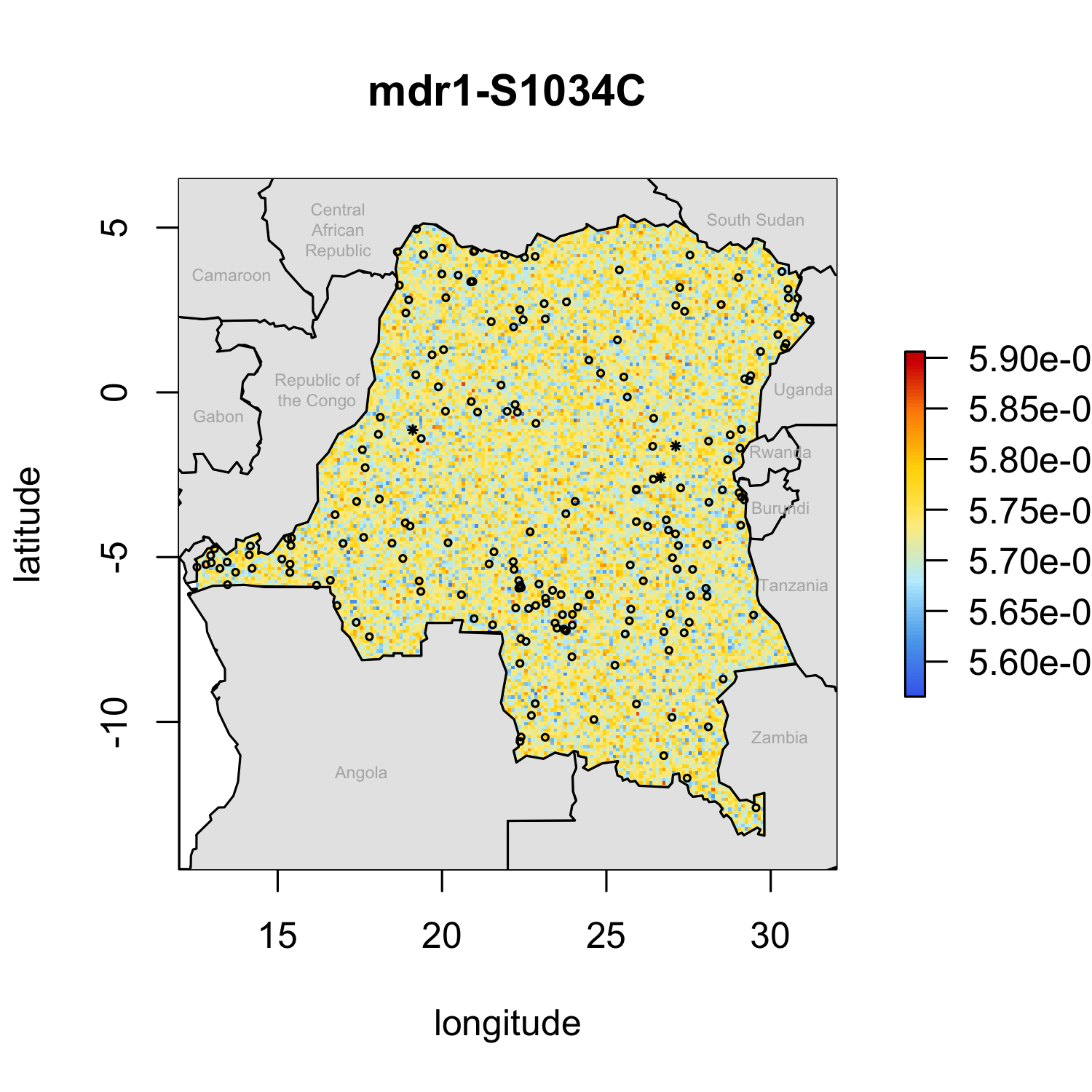 |
| 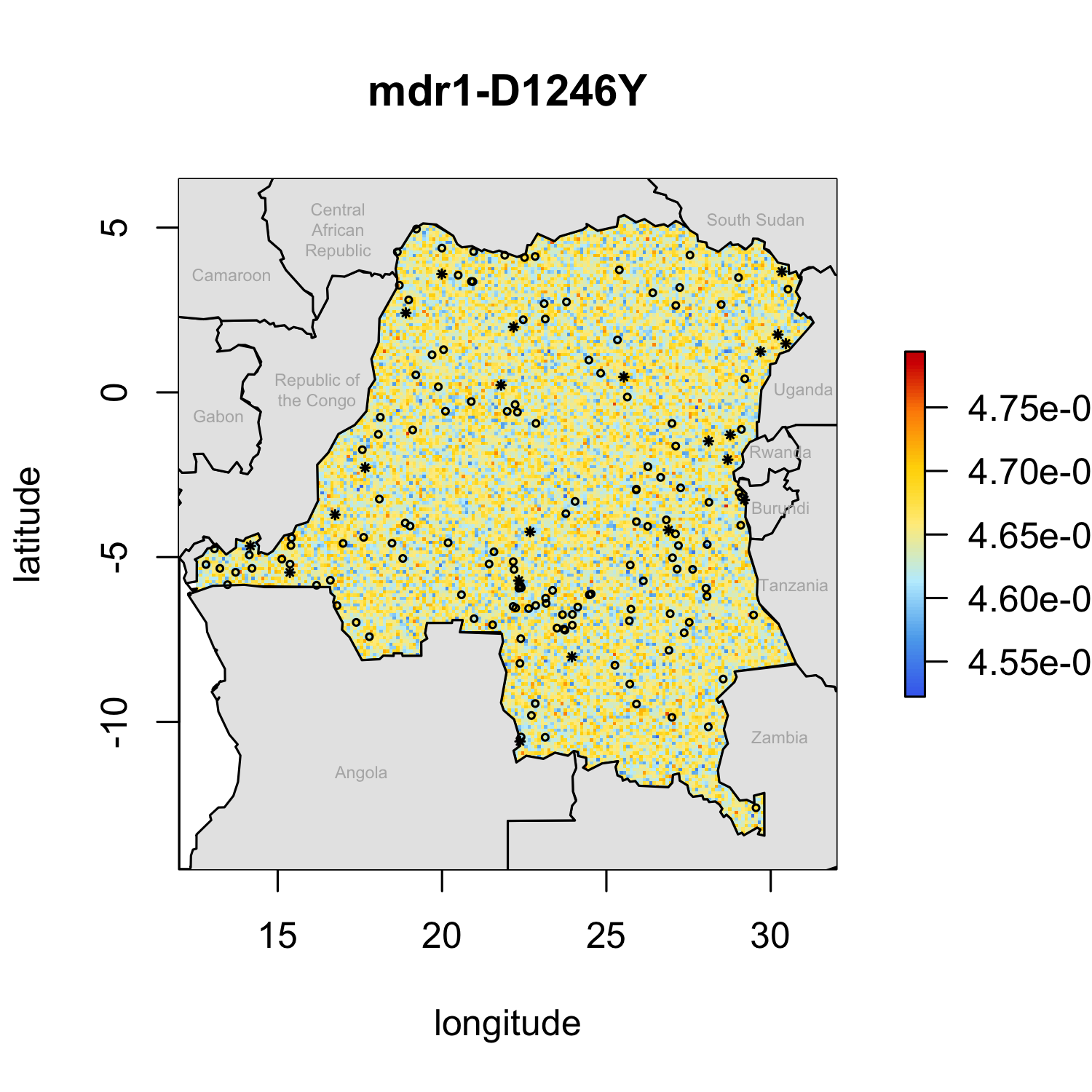 |  |  |  |
|  |  |  |  |

### ***Supplementary Figure 12. Standard error of predicted prevalence across the DRC of the drug resistance mutations***

For each known or candidate drug resistance locus, the associated standard error of prediction for the prevalence in [**Supplementary Figure 11**](https://docs.google.com/document/d/1sDEeFDTjTuoNVswYfyZofs1HwSmiI6H24MAdDdQ0pmI/edit#heading=h.e7p92oz44leb). Note that in some cases the maximum likelihood model parameters represent no spatial structure, in which case the mean prediction is uniform and the predictive standard errors are very low, however, the standard errors of model parameters (not shown) may still be high.
