# Supplementary material for: Drug-Resistance and Population Structure of Plasmodium falciparum Across the Democratic Republic of Congo Using High-Throughput Molecular Inversion Probes
Source: J Infect Dis. 2018 Apr 28;218(6):946–55. doi: 10.1093/infdis/jiy223 (PMC6093412; doi:10.1093/infdis/jiy223)
Supplement: Supplementary Methods [file jiy223_suppl_supplementary_methods.docx]

## **Supplementary Methods**

### ***P. falciparum MIP design***

A primer3 target template DNA sequence consisting of the target region and a variable length of flanking sequence depending on target region was created for each target. Publicly available SNP data was used to lowercase mask the template DNA at positions where SNP frequency was 0.01 or higher to avoid arm designs on variable regions. All possible arms flanking the target were designed using primer3 software [1]. Arm sequences were mapped to the reference 3d7 genome using bowtie2 software [2] and arms mapping to multiple locations were removed. All possible combinations of extension and ligation arms that satisfy the size criteria such that at least one sequencing read of 250 bp from either arm would fully traverse the target (the longest known repeat unit for the MS). Arms were scored for their sequence characteristics and GC content. Highest scoring arm pairs on each strand were selected and combined with an invariant backbone and random nucleotides (unique molecular identifiers, UMIs) to create a full length MIP as depicted in **Figure 1A.**

Based on these designs, oligonucleotides were synthesized as 200 nm ultramers (Integrated DNA Technologies, USA) with equimolar hand-mix option for random bases. Upon receipt, these were pooled at equal concentrations initially to create a MIP panel with desired probes. Following initial experiments, probes with lower efficiencies were spiked into the panel at different ratios, (**Supplementary Table 3**). MIPs were 5’ phosphorylated using 1 µl (10 units) T4 Polynucleotide Kinase (NEB, catalog # M0201) for every nanomole of probe, in 1X T4 DNA ligase buffer (NEB, catalog # B0202S) in a maximum of 50 µl reaction (bigger reactions were split). Phosphorylation reactions were incubated in a thermocycler at 37°C for 45 min followed by heat inactivation at 65°C for 20 min. Probes were aliquoted and kept at -20°C. Probes were diluted 1:8 in TE buffer to bring them to 1 µM working solution.

### ***MIP capture, amplification, and sequencing***

Capture reactions were carried out with modifications to published capture protocols [3] that improve capture of *P. falciparum* including using a different polymerase, decreasing reaction volume and shortening capture time (**Supplementary Figures 2-4**). Briefly, 10 µl capture reactions each sample and probe set containing Ampligase Buffer (1X), Phusion DNA polymerase (0.0008 units/µl), Ampligase (0.04 units/µl), pooled MIPs (40 nM, total), dNTP (4 µM), template DNA (up to 30 ng) were incubated in a preheated thermocycler with the following steps 95°C (10 min), 60°C (1 hr), 4°C hold. Next, 2 µl of exonuclease mix containing 1X Ampligase buffer, 10 units Exonuclease I and 50 units Exonuclease III were added to reactions following MIP captures. Reactions were performed in a thermocycler with the following steps 37°C (1 hr), 95°C (2 min), 4°C hold.

The entire capture reaction (12 µl) was amplified in a 25 µl PCR reaction containing following components: 1X Phusion Polymerase Buffer, 1X Macromolecular Crowding (MMC) solution, 200 nM dNTP, 0.02 units/µl Phusion DNA polymerase, forward and reverse primers (these primers contain Illumina sequencing adapters as well as 8 nucleotide long sample barcodes, **Supplementary Figure 1**, **Supplementary Table 4**), 500 nM each. PCR was performed using a preheated thermocycler with the following steps 98°C 30 s, 21 cycles (98°C 10 s, 63°C 30 s, 68°C 30 s), 68°C 2 min, 4°C hold. 50 ml 5X MMC was prepared by mixing the following components in water and filter-sterilized using 0.2 µ nylon syringe filter: 3.75 g Ficoll 70 (GE Healthcare, catalog # 17-0310-10), 1.25 g Ficoll 400 (Sigma catalog # F2637-5G), 0.125 g Polyvinylpyrrolidone (PVP360, Sigma catalog # PVP360-100g) [4].

Next, pools were created combining 5-10 µl of each PCR reaction in a single tube and cleaned up and concentrated using Ampure XP beads (Beckman Coulter, Catalog #A63881) at 0.8x bead:DNA ratio using manufacturer’s protocol. This generally removed the unwanted adapter/primer dimers ~ 200 bp. If dimers remained after bead clean up, the eluted DNA was loaded on a 1.5% agarose gel and relevant band between 500-1000 bp was extracted from the gel using Monarch DNA extraction kit (NEB, catalog # T1020S). Pools were sent to UMass Medical School Deep Sequencing Core and sequenced on Illumina MiSeq instrument using 250 bp paired end sequencing with dual indexing using MiSeq Reagent Kit v2 and custom primers **(Supplementary Table 4).**

### ***Comparison of MIP data with capillary electrophoresis (CE) data***

MIP MS lengths extracted from MIPWrangler output were converted to capillary electrophoresis (CE) PCR lengths for compatibility with control and previous studies. Reference PCR product sizes for CE experiments were calculated by mapping the CE PCR primers **(Supplementary Table 5)** to the Pf3D7 reference genome.

### ***MIP Microsatellite in silico and in vitro validation and probe selection***

MIP captures on 21 *Plasmodium falciparum* laboratory strains with available whole genome sequences were simulated using MIPWrangler software to obtain the expected captured sequences in order to calculate the expected MS lengths for each laboratory strain **(Supplementary Table 7)**. 4 selected laboratory strains (3D7, HB3, 7G8 and DD2) were experimentally typed using the MIP panel. Experimentally observed MS lengths for each MIP were compared to expected lengths from the simulation to determine accuracy of each MIP and probes for 8 MS loci were removed from the panel due to either producing low levels of false positive results in control experiments or having SNPs in hybrizing probe arms and consequently failing according to our simulations **(Supplementary Table 7)**. Sensitivity of the assays were determined from the microsatellites covered for each control sample. A microsatellite was considered covered if there were at least one sequence of expected length for one of the control strains. Abundance of a strain in a sample was determined for each MS only if that strain was uniquely identifiable by that MS according to the simulation. Strain frequencies for each sample were determined by averaging the frequencies of each MS. Lastly, AS4 MS was removed from further experiments due to lack of variation in this MS.

### ***References***

1. [Untergasser A, Cutcutache I, Koressaar T, et al. Primer3--new capabilities and interfaces. Nucleic Acids Res. **2012**; 40(15):e115.](http://paperpile.com/b/I8NBbx/RyB11)

2. [Langmead B, Salzberg SL. Fast gapped-read alignment with Bowtie 2. Nat Methods. **2012**; 9(4):357–359.](http://paperpile.com/b/I8NBbx/Y1W80)

3. [O’Roak BJ, Vives L, Fu W, et al. Multiplex targeted sequencing identifies recurrently mutated genes in autism spectrum disorders. Science. **2012**; 338(6114):1619–1622.](http://paperpile.com/b/I8NBbx/nj5Bm)

4. [Harve KS, Lareu R, Rajagopalan R, Raghunath M. Understanding how the crowded interior of cells stabilizes DNA/DNA and DNA/RNA hybrids-in silico predictions and in vitro evidence. Nucleic Acids Res. **2010**; 38(1):172–181.](http://paperpile.com/b/I8NBbx/pF5uG)
